# The circRNA circSEPT9 mediated by E2F1 and EIF4A3 facilitates the carcinogenesis and development of triple-negative breast cancer

**DOI:** 10.1186/s12943-020-01183-9

**Published:** 2020-04-07

**Authors:** Xiaying Zheng, Mengge Huang, Lei Xing, Rui Yang, Xiaosong Wang, Rong Jiang, Luyu Zhang, Junxia Chen

**Affiliations:** 1 Department of Cell Biology and Genetics, Chongqing Medical University, #1 Yixueyuan Road, Chongqing, 400016 China; 2 Department of Pathology, Sun Yat-Sen Memorial Hospital, Sun Yat-Sen University, #107 Yanjiang West Road, Guangzhou, 510120 China; 3 Department of Endocrine and breast surgery, The First Affiliated Hospital of Chongqing Medical University, #1 Yixueyuan Road, Chongqing, 400016 China; 4 Laboratory of Stem Cells and Tissue Engineering, Chongqing Medical University, #1 Yixueyuan Road, Chongqing, 400016 China; 5 Molecular Medicine and Cancer Research Center, Chongqing Medical University, #1 Yixueyuan Road, Chongqing, 400016 China

**Keywords:** circSEPT9, E2F1, EIF4A3, miR-637, Triple-negative breast cancer

## Abstract

**Background:**

Increasing studies have shown that circRNA is closely related to the carcinogenesis and development of many cancers. However, biological functions and the underlying molecular mechanism of circRNAs in triple-negative breast cancer (TNBC) remain largely unclear so far.

**Methods:**

Here, we investigated the expression pattern of circRNAs in four pairs of TNBC tissues and paracancerous normal tissues using RNA-sequencing. The expression and prognostic significance of circSEPT9 were evaluated with qRT-PCR and in situ hybridization in two TNBC cohorts. The survival curves were drawn by the Kaplan-Meier method, and statistical significance was estimated with the log-rank test. A series of in vitro and in vivo functional experiments were executed to investigate the role of circSEPT9 in the carcinogenesis and development of TNBC. Mechanistically, we explored the potential regulatory effects of E2F1 and EIF4A3 on biogenesis of circSEPT9 with chromatin immunoprecipitation (ChIP), luciferase reporter and RNA immunoprecipitation (RIP) assays. Furthermore, fluorescent in situ hybridization (FISH), luciferase reporter and biotin-coupled RNA pull-down assays were implemented to verify the relationship between the circSEPT9 and miR-637 in TNBC.

**Results:**

Increased expression of circSEPT9 was found in TNBC tissues, which was positively correlated with advanced clinical stage and poor prognosis. Knockdown of circSEPT9 significantly suppressed the proliferation, migration and invasion of TNBC cells, induced apoptosis and autophagy in TNBC cells as well as inhibited tumor growth and metastasis in vivo. Whereas up-regulation of circSEPT9 exerted opposite effects. Further mechanism research demonstrated that circSEPT9 could regulate the expression of Leukemia Inhibitory Factor (LIF) via sponging miR-637 and activate LIF/Stat3 signaling pathway involved in progression of TNBC. More importantly, we discovered that E2F1 and EIF4A3 might promote the biogenesis of circSEPT9.

**Conclusions:**

Our data reveal that the circSEPT9 mediated by E2F1 and EIF4A3 facilitates the carcinogenesis and development of triple-negative breast cancer through circSEPT9/miR-637/LIF axis. Therefore, circSEPT9 could be used as a potential prognostic marker and therapeutical target for TNBC.

## Background

Breast cancer is the most common cause of cancer-related mortality in women around the world. Triple-negative breast cancer (TNBC) is a subgroup of breast cancer, which is defined as lack of estrogen receptor (ER), progesterone receptor (PR) and human epidermal growth factor receptor 2 (HER2). This subgroup comprises around 15~20% of all breast cancers [1]. TNBC is characterized by high rates of cell proliferation and metastasis and poor prognosis. The main therapeutic strategies are conventional chemotherapy and radiation. To date, no targeted therapy for TNBC has been approved by Food and Drug Administration (FDA) [2, 3]. Therefore, it is urgent to find feasible molecular target for TNBC.

CircRNAs are covalently closed single stranded circular transcripts formed by precursor mRNA back-splicing or skipping events with no 5′ caps and 3′ poly(A) tail. High-throughput sequencing and bioinformatics analysis have revealed the widespread expression of circRNAs across various species. Because of high stability, abundance and evolutionary conservation, circRNAs have obvious advantages as biomarkers of clinical diseases [4]. It has been found that circRNAs have a series of important biological functions including microRNA sponges, RNA-binding protein (RBP) sponges, protein/peptide translation templates, gene transcription and RNA splicing regulators [5]. Mounting evidence shows that circRNAs play vital roles in carcinogenesis and cancer progression. Li et al. demonstrated that circDDX17 could act as a tumor suppressor in colorectal cancer, circDDX17 was significantly down-regulated in CRC tissues and correlated with adverse clinical pathological parameters [6]. In addition, circHMGCS1 could promote the proliferation of hepatoblastoma cells by regulating the IGF signaling pathway and glutaminolysis and circHMGCS1 might be a potential prognostic marker and therapeutic target for hepatoblastoma [7]. It has been reported that circANKS1B mediated by splicing factor ESRP1 promotes breast cancer metastasis. circANKS1B acts as a prognostic factor and mediator to regulate the progression of triple-negative breast cancer [8]. Moreover, circRNAs are enriched and stable in exosomes, which lays a foundation for the development of circRNAs as new type of tumor biomarkers [9]. It has been demonstrated that circRNAs are produced by a non-canonical alternative splicing, and RNA-binding proteins (RBPs) are essential for the generation of circRNAs [10]. However, to date, the potential functions, biogenesis processes and underlying mechanisms of circRNAs in TNBC remain elusive.

MicroRNAs (miRNAs) are highly conserved small non-coding RNA with 18–25 nucleotides and can regulate gene expression at post-transcription level by inhibiting protein translation or degrading target mRNAs. As we all know, microRNAs can serve as either an oncogene or a tumor suppressor in the occurrence and development of various cancers, including breast cancer [11, 12]. However, little is known about the upstream regulatory mechanism of microRNA. In recent years, a novel regulatory mechanism has been proposed, which is called the competing endogenous RNA (ceRNA) hypothesis. In this hypothesis, RNA transcripts can communicate and regulate each other’s expression through competing shared microRNAs response elements (MREs) [13]. Studies have indicated that more than 80% of circRNAs come from exons and share the same sequences with the corresponding linear RNAs. Therefore, circRNAs, as a new member of ceRNA family and miRNA activity regulator, may play a significant role in gene expression regulation in cancers and other diseases [14, 15]. Emerging studies have revealed that dysregulated expression of key circRNAs could disturb the balance of ceRNA network, leading to the tumorigenesis and development of cancer. For example, circular RNA circUBXN7 inhibits cell growth and invasion via sponging miR-1247-3p to increase B4GALT3 expression in bladder cancer [16]. Moreover, circTADA2As suppress development and metastasis of breast cancer by targeting miR-203a-3p/SOCS3 axis [17]. Besides, circSETD3 serves as a ceRNA for microRNA-421 to inhibit hepatocellular carcinoma tumorigenesis [18].

Here, we utilized RNA-seq to analyze the expression profile of circRNAs in TNBC tissues, and identified a new circRNA termed circSEPT9 (circBase ID: hsa_circ_0005320). We determined that circSEPT9 was highly expressed in TNBC tissues/cells and positively related to advanced clinical stage and poor prognosis. More importantly, we found that E2F1 and EIF4A3 might mediate the biogenesis of circSEPT9. In vitro and in vivo experiments showed that circSEPT9 could promote cell growth and metastasis. We further demonstrated that circSEPT9 up-regulated the expression of LIF via sponging miR-637, which could contribute to development of TNBC through activating LIF/Stat3 signaling pathway. Our data suggest that circSEPT9 might be an independent prognostic marker and promising therapy target for TNBC.

## Materials and methods

### Human TNBC tissue specimens

The TNBC tissues and paracancerous tissues used in this study were provided by TNBC patients who had undergone surgical resection at the First Affiliated Hospital of Chongqing Medical University (Chongqing, China). The tissue specimens were immediately immersed in liquid nitrogen until use. All patients received written informed consent. The study was authorized by the Ethics Committee of Chongqing Medical University.

### Cell lines and culture conditions

Five human TNBC cell lines, MDA-MB-231, BT-549, MDA-MB-468, MDA-MB-453 and SUM-159, as well as normal breast epithelial cell line MCF-10A were purchased from ATCC. 293 T and 293 cell lines were conserved in our laboratory. MDA-MB-231, MDA-MB-468, MDA-MB-453, SUM-159, 293 T and 293 cells were cultured in DMEM medium (Gibco, Carlsbad, CA, USA), BT-549 cells were grown in RPMI 1640 medium (Gibco, Carlsbad, CA, USA). MCF-10A cells were maintained in mammary epithelial cell growth medium (MEGM) BulletKit (Lonza, Basel, Switzerland). The cell media contained 10% fetal bovine serum (FBS, HyClone, Invitrogen), 100 U/ml penicillin and 100 mg/ml, cells were maintained in a humidified incubator at 37 °C with 5% CO_2_.

### Plasmid construction, RNAi and cell transfection

To construct circSEPT9 overexpression vector, the full-length of human circSEPT9 was inserted into the pLCDH-ciR vector (Geenseed Biotech, Guangzhou, China), which contained a front and back circular frame, whereas the mock vector with no circSEPT9 sequence was used as a control. To knock down circSEPT9, siRNAs targeting back splice junction of circSEPT9 (si1-circ, si2-circ, si3-circ) and a siRNA-NC were synthesized (Geenseed Biotech, Guangzhou, China). The most effective siRNA si1-circ detected by qRT-PCR was subcloned into the lentivirus vector (pHBLV-U6-MCS-CMV-ZsGreen-PGK-Puro) to construct sh-circSEPT9 vector, while sh-NC was used as the negative control. The lentiviral vector carrying circSEPT9 or sh-NC and two assistant vectors were transiently transfected into HEK293T cells. Viral supernatants were collected 48 h later, clarified and concentrated for animal studies. After that, the stably transfected or infected TNBC cells were screened with puromycin. The pcDNA3.1-EIF4A3 and pcDNA3.1-E2F1 vectors and shRNAs aimed at EIF4A3 were purchased from Sangon Biotech (Shanghai, China). The vectors above were verified by sequencing. E2F1 siRNAs, hsa-mir-637 mimics and inhibitor were bought from GenePharma (Shanghai, China). MiR-NC and inh-NC were used as controls. All transfections were executed with Lipofectamine 2000 (Invitrogen, Carlsbad, CA, USA) following the manufacturer’s instructions. The sequences of siRNAs and shRNAs in this study were listed in Additional file 1: Table S1.

### RNA sequencing of circRNA extracted from human TNBC tissues

The total RNA was extracted from 4 pairs of fresh frozen TNBC tissues and para-carcinoma tissues using TRIzol reagent (Takara, Dalian, China). Next, RNA was purified by rRNA depletion, followed by cDNA synthesis and RNA amplification. The RNA-seq libraries were constructed and sequenced utilizing the Illumina HiSeq2500 platform (Illumina, San Diego, USA) according to the manufacturer’s protocol.

### RNA extraction, nuclear-cytoplasmic fractionation, RNase R and actinomycin D treatment and qRT-PCR assays

Isolation of total RNA from tissues or cell lines was performed by using TRIzol reagent (Takara, Dalian, China). RNAs from nucleus and cytoplasm of TNBC cells were separated by the PARIS™ Kit (Life Technologies, Austin, Texas, USA) following the manufacturer’s instructions. RNase R treatment was executed at 37 °C with 4 U/μg of RNase R (Epicentre Biotechnologies, Madison, WI, USA) for 10 min, 20 min, 30 min and 40 min, respectively. In addition, total RNA from TNBC cells was treated with 100 ng/ml actinomycin D (Cell Signaling Technology, Beverly, MA, USA) against new RNA synthesis for 12 h and 24 h. RNA was reversed transcribed into cDNAs with the PrimeScript RT Reagent Kit (Takara, Dalian, China) according to the manufacturer’s instructions. Quantitative real-time PCR (qRT-PCR) analysis was performed by the TB Green Premix Ex Taq (Takara, Dalian, China). Relative expression levels of genes were quantified using the 2^−ΔΔCt^ method. The primer sequences were displayed in Additional file 1: Table S2.

### Fluorescence in situ hybridization (FISH)

Cy3-labeled circSEPT9 (5′-AGATCTTTTCAAGGCCTCCTGGCT-3′) and FITC-labeled hsa-miR-637 probes (5′-ACGCAGAGCCCGAAAGCCCCCAGT-3′) (Geneseed, Guangzhou, China) were used to observe the co-localization of circSEPT9 and hsa-miR-637 in TNBC tissues and cells. FISH analysis was performed using a Fluorescent In Situ Hybridization Kit (Geneseed, Guangzhou, China) according to the manufacturer’s instructions. Cell nuclei were stained with 4,6-diamidino-2-phenylindole (DAPI, Beyotime, China). The images were photographed under the fluorescence microscope (Leica, Wetzlar, Germany).

### Tissue microarray (TMA) and in situ hybridization (ISH)

TMAs, consisting of 80 paraffin-embedded TNBC specimens, were obtained from Outdo Biotech (Shanghai, China). ISH was utilized to determine the relative expression of circSEPT9 in 80 specimens by means of the manufacturer’s instructions. In brief, the TMAs were disposed in the process of dewaxing, rehydration and digestion, followed by hybridizing with the specific circSEPT9 probe (5’DIG-AGATCTTTTCAAGGCCTCCTGGCT-3’DIG) (Geneseed, Guangzhou, China). The samples were incubated with anti-Digoxin-AP (Roche, Basel, Switzerland) and stained by NBT/BCIP (Roche, Basel, Switzerland). Finally, the expression of circSEPT9 were quantified and imaged in TMAs from TNBC. The ISH staining score was calculated by multiplying the value for intensity of positive staining (negative = 0, weak = 1, moderate = 2 and strong = 3) and the proportion of positively stained cells (< 10% = 0, 10–25% = 1, 26–50% = 2, 51–75% = 3, > 75% = 4). The ISH score < 6 indicated low expression, while ≥6 defined high expression.

### Luciferase reporter assay

The sequences of circSEPT9 or LIF 3’UTR containing the wild-type (WT) or mutant (Mut) binding site of hsa-miR-637 were devised and synthesized by GenePharma (Shanghai, China). 293 T or 293 cells were co-transfected with the corresponding plasmids and hsa-miR-637 mimics/miR-NC or hsa-miR-637 inhibitors/inh-NC with Lipofectamine 2000 (Invitrogen, Carlsbad, CA, USA). To construct of a luciferase reporter gene vector containing SEPT9 promoter, the full-length SEPT9 promoter containing wide or mutant type was respectively cloned into pGL3-basic vectors (Genecreate, Wuhan, China), and co-transfected with or without E2F1 overexpression vector later. After 48 h of incubation, the activities of firefly and Renilla luciferase were measured using the Dual Luciferase Reporter Assay Kit (Promega, Madison, WI, USA).

### Cell proliferation, wound healing, migration and invasion assays

CCK-8, EDU, colony formation, wound healing, transwell migration and invasion assays were performed as previously reported [19, 20].

### Cell cycle and apoptosis assays

For cell cycle analysis, cells were harvested and fixed in pre-cold 70% ethanol at 4 °C overnight, then stained with propidium iodide (PI) and measured by the flow cytometry (Becon Dickinson FACS Calibur, NY, USA). The cells were stained with Annexin V-FITC and PI and subsequently the ratio of apoptotic cells was tested by flow cytometry. In addition, cell apoptosis was further detected with TUNEL and Hoechst 33342 staining (Beyotime, Shanghai, China) according to the manufacturer’s protocols. The images were observed under a fluorescence microscope (Leica, Wetzlar, Germany).

### Chromatin immunoprecipitation (ChIP)

The crosslinking reaction was terminated by glycine in cells treated with 1% formaldehyde. Next, the resultant DNA-protein complex was sonicated to produce 200–1000 bp DNA fragments. The anti-E2F1 antibody (Abcam, Burlingame, CA, USA) was added to form the antibody-target protein-DNA complex and protein A-Sepharose beads were used to immunoprecipitate complex. After washing and reversing the cross-links, the enriched DNA was purified and then examined by qRT-PCR. The primer sequences were listed in Additional file 1: Table S2.

### RNA immunoprecipitation (RIP)

RIP assay was carried out by Magna RIP kit (Millipore, Billerica, MA, USA) adhering to the manufacturer’s guidelines. Briefly, the magnetic beads were incubated with anti-EIF4A3 antibodies (Abcam, Burlingame, CA, USA) or IgG negative control antibody (Millipore, Billerica, MA, USA). Subsequently, cells were lysed and incubated with the corresponding antibody-coated beads, and then the coprecipitated RNA was extracted using TRIzol regent (Takara, Dalian, China) and detected by qRT-PCR.

### Biotin-coupled probe RNA pull down assay

To pull down the miRNA by circRNA, biotinylated-circSEPT9 probe (5′-AGATCTTTTCAAGGCCTCCTGGCTCCGGGGTGTAGCCTC-3′) was synthesized by RiboBio (Guangzhou, China), while oligo probe (5′-TATCACGTAGCCGTTGCATTTGCCGTAGCCCTGTGGGCC-3′) was considered as a control. Approximately 1 × 10^7^ MDA-MB-231 cells transfected with miR-637 mimics were lysed and incubated with biotin-labeled circSEPT9 probe. After that, the biotin-coupled RNA complex was pull-downed by streptavidin-coated magnetic beads adsorption. The enriched circSEPT9 and miR-637 were analyzed by qRT-PCR and RT-PCR. To pull down the circRNA by miRNA, biotinylated miRNA mimics or their mutants were synthesized by RiboBio (Guangzhou, China). 1 × 10^7^ cells with circSEPT9 overexpression were transfected with biotinylated miRNA mimics or their mutants respectively and incubated for 48 h, streptavidin-coated magnetic beads were washed with lysis buffer and Trizol (Takara, Dalian, China) was used to purify RNA complex. The abundance of circSEPT9 was detected by qRT-PCR and RT-PCR.

### Western blot analysis

Briefly, the proteins were extracted, quantified and isolated by 10% SDS-PAGE. Next, the separated protein bands were transferred to PVDF membranes (Bio-Rad, CA, USA). The membranes were blocked with 4% skim milk powder and then incubated with primary antibodies against LIF (1:1000 dilution) (R&D Systems, Minneapolis, MN), STAT3 (1:1500 dilution), MDM2 (1:1000 dilution), Cyclin E1 (1:1000 dilution) (Abcam, Burlingame, CA, USA), ID1 (1:500 dilution) (Bioss, Beijing, China), P-STAT3 (1:2000 dilution), P53 (1:1000 dilution), P21 (1:1000 dilution), Bax (1:1000 dilution), Bcl-2 (1:1000 dilution), Caspase-3 cleaved (1:1000 dilution), LC3B (1:1000 dilution), ATG5 (1:1000 dilution), ATG7 (1:1000 dilution), Cyclin D1 (1:1000 dilution), CDK4 (1:1000 dilution) and GAPDH (1:5000 dilution) (Cell Signaling Technology, Beverly, MA, USA) overnight at 4 °C, followed by incubating with a secondary antibodies (1:5000 dilution) (Cell Signaling Technology, Beverly, MA, USA) for 2 h. Finally, the bands were detected by chemiluminescence.

### Animal experiments

The 4-week-old female BALB/c nude mice were chosen for xenografts experiments and maintained in specific pathogen-free conditions. All procedures were approved by Chongqing Medical University Animal Care and Use Committee. The mice were subcutaneously inoculated with MDA-MB-231 cells stably transfected with circSEPT9 overexpression/mock vector or infected with lentiviruses (Hanbio Co.LTD, Shanghai, China) carrying sh-circSEPT9/sh-NC, respectively (2.5 × 10^6^, 200ul). After 28 days, the mice were sacrificed. The tumor volume was calculated in the accordance with the formula (length × width^2^/2) and measured weekly and tumor weight was determined. The tumors and lungs were collected for further study. Metastatic nodules of the lung were counted under microscope. The microvessels were counted on HE-stained slides from the tumors under microscope corresponding to areas with the highest vascular density. For survival analysis, the mice were injected subcutaneously with cells stably transfected with circSEPT9 or mock vector respectively, and were monitored for 60 days as a cutoff. After 2 months, the mice still alive were considered to be censored, the mice were sacrificed and livers were removed for pathology analysis.

### Immunohistochemistry (IHC) and immunofluorescence (IF)

IHC and IF assays were performed as previously reported [19]. For IF experiment, tissues or cells were incubated with primary antibodies against LIF (1:100 dilution) (Bioss, Beijing, China) and LC3B (1:200 dilution) (Cell Signaling Technology, Beverly, MA, USA) at 4 °C overnight, then incubated with fluorescein Alexa-Fluor 488-conjugated secondary antibodies and imaged by using fluorescence microscope (Leica, Wetzlar, Germany). For IHC assay, paraffin sections were incubated with antibodies against LIF (1:100 dilution) (R&D Systems, Minneapolis, MN), ID1 (1:400 dilution) (Bioss, Beijing, China), MDM2 (1:50 dilution) (Abcam, Burlingame, CA, USA), P-STAT3 (1:200 dilution), P53 (1:160 dilution) and P21 (1:50 dilution) (Cell Signaling Technology, Beverly, MA, USA). Images were observed under Olympus multifunction microscope (Olympus BX51, Tokyo, Japan).

### Statistical analysis

Statistical analyses were mainly conducted by SPSS 21.0 (IBM, SPSS, Chicago, IL, USA) and GraphPad Prism 6.0 (GraphPad Software Inc., CA, USA). The differences between groups were analyzed using Student’s t test, one-way ANOVA or chi-square test. The correlation between groups was assessed with Pearson correlation coefficient. The diagnostic value was evaluated by the receiver operating characteristic (ROC) curve. Univariate and multivariate Cox proportional hazards regression models were devoted to assess the impacts of the clinical variables on overall survival of TNBC patients. Kaplan-Meier method was used for survival analysis and log-rank test determined statistical significance.

## Results

### CircSEPT9 is identified and generated from exon 2 of SEPT9 by back-splicing

**Fig. 1:**
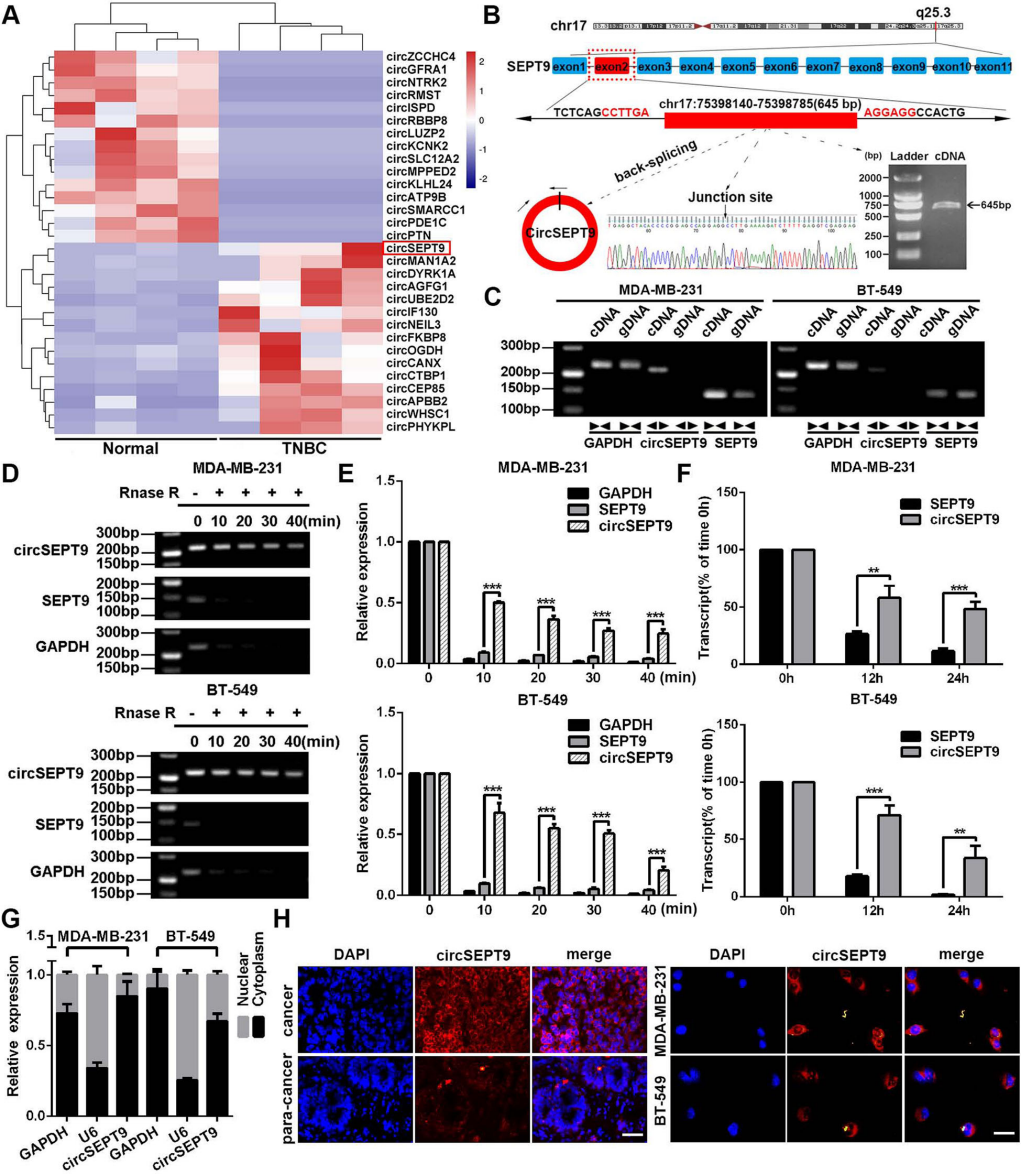
circSEPT9 is validated and characterized in TNBC cells. **a** The cluster heat maps showed the top 15 differentially expressed circRNAs in 4 pairs of human TNBC tissues and adjacent normal tissues. The red and blue strips indicate up-regulated and down-regulated circRNAs, respectively. **b** Schematic illustration of circSEPT9 formation via the circularization of exons 2 in SEPT9 gene. The back-splice junction sequences and RT-PCR product of circSEPT9 were validated by Sanger sequencing and agarose gel electrophoresis, respectively. **c** PCR was performed to detect the existence of circSEPT9 and SEPT9 from cDNA and gDNA in TNBC cells using the divergent and convergent primers, respectively. Divergent primers amplified circSEPT9 in cDNA but not genomic DNA (gDNA). **d** and **e** PCR and qRT-PCR were conducted to determine the abundances of circSEPT9 and linear SEPT9 mRNA in TNBC cells treated with RNase R at the indicated time points. **f** The RNA expressions of circSEPT9 and SEPT9 of TNBC cells were analyzed by qRT-PCR after treatment for 12 h and 24 h with actinomycin D. **g** Nuclear-cytoplasmic fractionation assay indicated that circSEPT9 was mainly localized in the cytoplasm of TNBC cells. GAPDH was considered as a cytoplasmic protein control and U6 was used as a nuclear control. **h** The localization of circSEPT9 was observed in TNBC tissues (magnification, × 100, Scale bar, 50 μm) and cells (magnification, × 200, Scale bar, 50 μm) by FISH. The nuclei were stained with DAPI. The data are presented as the mean ± SD, **P < 0.01, ***P < 0.001

To identify and characterize differentially expressed circRNAs in TNBC, RNA-seq was implemented in 4 pairs of TNBC and adjacent noncancerous tissues. When we set the filter criteria as a fold-change ≥2 and a *P*-value < 0.05, we found 354 differentially expressed circRNAs, of which 47 were up-regulated and 307 were down-regulated in TNBC tissues (Additional file 1: Table S3). Among them, the top 30 dysregulated circRNAs were shown in Fig. 1a. In the top 15 upregulated circRNAs, we focused on a novel circRNA, circSEPT9 (hsa_circ_0005320), which was generated from exon2 of SEPT9 gene by back-splicing (645 bp) on the basis of the annotation of circBase (http://www.circbase.org/), its back splicing junction was validated by Sanger sequencing and the presence of circSEPT9 was proved by RT-PCR (Fig. 1b). To make sure circSEPT9 from the head-to-tail splicing instead of trans-splicing or genomic rearrangements, the divergent and convergent primers were designed to amplify circSEPT9 circular transcripts and SEPT9 linear transcripts, respectively. PCR results showed that circSEPT9 was only detected in cDNA, thus ruling out the existence of circSEPT9 in gDNA, whereas the convergent primers amplified SEPT9 from both cDNA and gDNA (Fig. 1c). In addition, we found that circSEPT9 was more resistant to RNase R digestion than linear SEPT9 and GAPDH transcripts (Fig. 1d and e). To further evaluate the stability of circSEPT9, actinomycin D were used to inhibit the synthesis of new RNA. The results showed that circSEPT9 was more stable than linear SEPT9 after treatment with actinomycin D (Fig. 1f). These results suggest that circSEPT9 is a circular RNA. Subsequently, the subcellular localization of circSEPT9 was detected in TNBC cells and tissues by nuclear-cytoplasmic fractionation and FISH assays. It was found that circSEPT9 was mainly present in the cytoplasm of TNBC cells (Fig. 1g and h).

### E2F1 and EIF4A3 promotes expression of circSEPT9

**Fig. 2:**
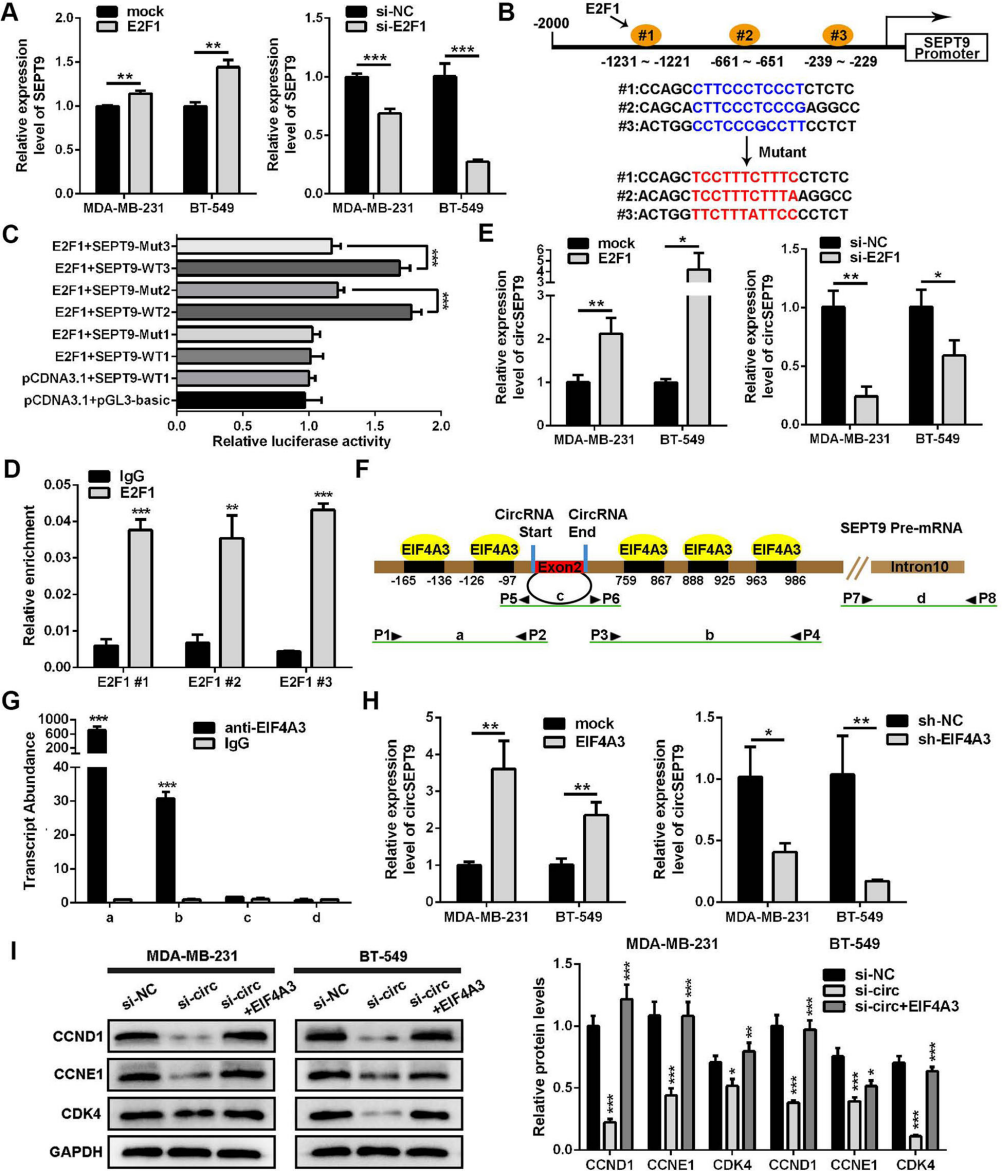
E2F1 and EIF4A3 enhance the expression of circSEPT9 through the binding to the SEPT9 promoter and pre-mRNA, respectively. **a** The expression of SEPT9 was detected in TNBC cells transfected with E2F1 overexpression plasmids or siRNAs targeting E2F1 by qRT-PCR. **b** Schematic illustration of wild type (WT) and mutant (Mut) sequences of three putative binding sites of E2F1 on SEPT9 promoter are shown. **c** The relative luciferase activities were detected in MDA-MB-231 cells co-transfected with luciferase reporter plasmids containing wild type or mutant SEPT9 promoter sequence and overexpression plasmids of E2F1. **d** ChIP-qPCR assays were performed to determine which putative E2F1 binding site the SEPT9 promoter was bound to in MDA-MB-231 cells, IgG was used as a negative control. **e** The circSEPT9 expression was valued in TNBC cells transfected with E2F1 overexpression plasmids or si-E2F1 by qRT-PCR. **f** and **g** The putative binding sites of EIF4A3 in the upstream and downstream region of the circSEPT9 pre-mRNA were predicted with circInteractome database, and the RIP assay confirmed that EIF4A3 could directly bind to the SEPT9 pre-mRNA in MDA-MB-231 cells. The intron 10 of the SEPT9 pre-mRNA and IgG were used as the negative controls. **h** circSEPT9 expression was detected in TNBC cells after EIF4A3 up-regulation or down-regulation by qRT-PCR. **i** The expression of cell cycle-related proteins in TNBC cells co-transfected with EIF4A3 expression vector and si-circSEPT9 was higher than that in TNBC cells transfected with si-circSEPT9 alone by western blot. The data are presented as the mean ± SD, *P < 0.05, **P < 0.01, ***P < 0.001

To explore the effects of E2F1 and EIF4A3 on the expression of circSEPT9 in TNBC, E2F1 and EIF4A3 overexpression or knockdown plasmids or siRNAs were constructed and synthesised respectively, relative expression levels of E2F1 and EIF4A3 were significantly up-regulated or down-regulated in TNBC cells transfected with the corresponding plasmids by qRT-PCR (Additional file 2: Figure S1a and b). E2F1 is a member of the E2F family of transcription factors and it can regulate the expression of target genes at transcriptional level by binding to their promoter. We found 13 putative E2F1-binding sites on SEPT9 promoter through bioinformatics prediction (http://jaspar.genereg.net) and selected the three closest binding sites for further study. Therefore, we speculated that E2F1 might regulate the expression of SEPT9 at the transcription level. The results showed that SEPT9 expression was significantly up-regulated in TNBC cells transfected with the E2F1 expression vectors, whereas si E2F1 led to a decrease in the level of SEPT9 (Fig. 2a). After that, we constructed a series of luciferase reporter vectors containing the full-length of the wild type (WT) or mutant (Mut) SEPT9 promoter (Fig. 2b). Luciferase reporter assays displayed that E2F1 promoted the luciferase activity of wild type SEPT9 promoter but not mutant SEPT9 promoter (Fig. 2c). Besides, ChIP-qPCR analysis demonstrated that E2F1 could bind to the promoter of SEPT9 gene and accelerate its transcription activity (Fig. 2d). To further explore whether the expression of circSEPT9 can be regulated by the binding of E2F1 to the SEPT9 promoter, we carried out qRT-PCR analysis in TNBC cells transfected with E2F1 expression vector and si E2F1. The results revealed that up-regulation of E2F1 significantly enhanced circSEPT9 expression, whereas knockdown of E2F1 suppressed circSEPT9 level (Fig. 2e). These data demonstrated that SEPT9 is the target gene of transcription factor E2F1.

Next, we discovered 5 putative binding sites of EIF4A3 in the upstream and downstream region of the circSEPT9 mRNA transcript (circSEPT9 pre-mRNA) via Circinteractome (https://circinteractome.nia.nih.gov/index.html). EIF4A3 is a core component of the exon junction complex and play an essential role in pre-mRNA splicing. Then, we defined two putative EIF4A3 binding sites in the upstream of circSEPT9 pre-mRNA as a, three putative EIF4A3 binding sites in the downstream of circSEPT9 pre-mRNA as b, the sequence on circSEPT9 as c and the sequence on SEPT9 pre-mRNA intron 10 as d (Fig. 2f). RIP assay demonstrated that EIF4A3 could bind to SEPT9 pre-mRNA through these putative binding sites by anti-EIF4A3 antibody, but not by negative control antibody IgG (Fig. 2g). Furthermore, qRT-PCR assay showed that overexpression of EIF4A3 facilitated the expression of circSEPT9, while knockdown of EIF4A3 inhibited the level of circSEPT9 in TNBC cells (Fig. 2h). In addition, western blot analysis displayed that knockdown of circSEPT9 suppressed the expression of cell cycle-related proteins in TNBC cells and the effects could be reversed by co-transfecting TNBC cells with EIF4A3 expression vector (Fig. 2i). Therefore, EIF4A3 might promote the expression of circSEPT9 to modulate cell cycle.

### circSEPT9 is highly expressed in TNBC and associated with clinical characteristics

**Fig. 3:**
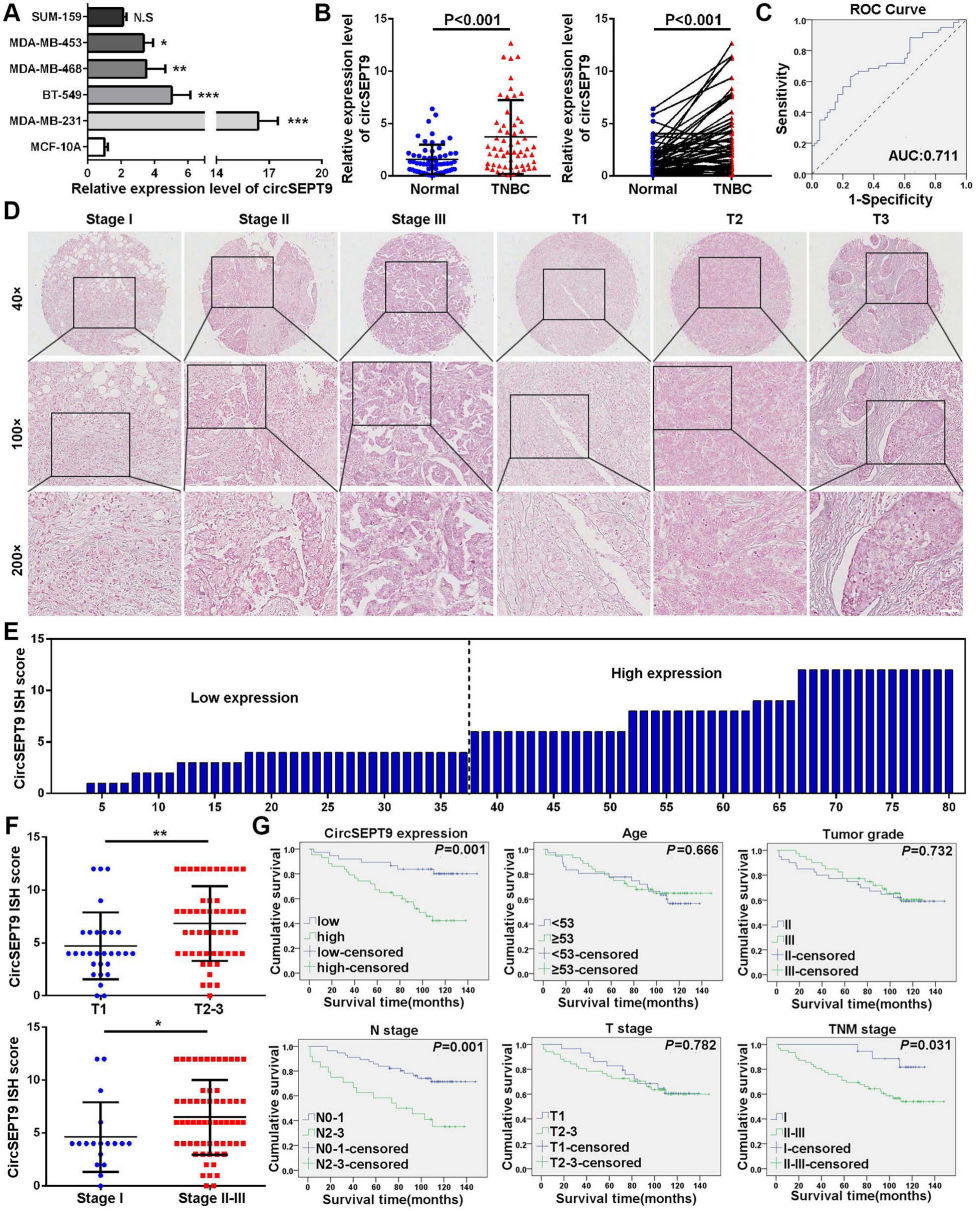
circSEPT9 is up-regulated in TNBC and related with progression and poor prognosis of TNBC patients. **a** and **b** The relative expression of circSEPT9 in TNBC cells and 60 paired TNBC tissues and adjacent normal tissues by qRT-PCR. **c** ROC curve was applied to evaluate the diagnostic value of circSEPT9 for TNBC. **d** Representative images showed the expression of circSEPT9 in 80 TNBC tissues detected by in situ hybridization. Scale bar, 50 μm. **e** The ISH staining scores were calculated in 80 TNBC tissues. ISH staining score < 6 was defined as low expression, while a score ≥ 6 was regards as high expression. **f** Dot distribution graph of circSEPT9 IHC staining scores was shown in 80 TNBC patients of different clinical stages. **g** Kaplan-Meier survival curve analysis showed that the total survival time of TNBC patients with high expression of circSEPT9 was significantly shorter than that of TNBC patients with low expression of circSEPT9. The total survival was significantly correlated with TNM stage and lymph node metastasis, but not with age, histological grade and tumor size. The data are presented as the mean ± SD, *P < 0.05, **P < 0.01, ***P < 0.001

To analyze the expression and clinical value of circSEPT9, qRT-PCR was conducted to determine the expression levels of circSEPT9 in 60 pairs of TNBC and the adjacent normal tissues. The results demonstrated that circSEPT9 was significantly higher expressed in TNBC cell lines (MDA-MB-231, BT-549, MDA-MB-453 and MDA-MB-468) and tissues than normal breast epithelial cells (MCF-10A) and adjacent normal tissues (Fig. 3a and b), which was consistent with the results of RNA-Seq. Receiver operating characteristic (ROC) curve was further utilized to assess the diagnostic value of circSEPT9 for TNBC screening. According to the ROC curve analysis, the area under the curve (AUC) of circSEPT9 was 0.711, and the specificity and sensitivity were 75.0 and 63.3%, respectively, at a cut-off value of 1.971 (Fig. 3c). Subsequently, we evaluated the relationship between the expression of circSEPT9 and clinical pathological characteristics. The results displayed that the expression of circSEPT9 was positively correlated with T (*P* = 0.002), N (*P* = 0.028) and TNM (*P* = 0.001) stages, but not with other clinical pathological parameters including age, menopausal status and grade (Table 1).

**Table 1:** Correlation between circSEPT9 expression and clinicopathological features in 60 TNBC patients (cohort 1)

Characteristic		All cases	circSEPT9		Chi-square	*P* value
			low	high		
All cases		60	30	30		
Age	< 53	28	16	12	1.071	0.301
	≥53	32	14	18		
Menopausal	Premenopausal	20	11	9	0.454	0.500
	Postmenopausal	33	15	18		
Grade	II	29	17	12	1.307	0.253
	III	17	7	10		
T stage	T1	24	18	6	10	0.002**
	T2–3	36	12	24		
N stage	N0	40	24	16	4.8	0.028*
	N1–3	20	6	14		
TNM stage	I	20	16	4	10.8	0.001**
	II/III	40	14	26		

* *P* < 0.05,**P < 0.01

Furthermore, we used another cohort of 80 TNBC tissues on TMAs to detect the expression of circSEPT9 with ISH (Fig. 3d and e). The correlation between circSEPT9 expression and clinicopathological characteristics of the TNBC patients were listed in Table 2. Further analysis indicated that there was also a positive association between circSEPT9 expression and T stage (*P* = 0.009) and TNM (*P* = 0.002) stage (Fig. 3f). Kaplan-Meier survival curves showed that patients with high expression of circSEPT9 (*P* = 0.001), N2–3 stage (*P* = 0.001) and TNM stage II/III (*P* = 0.031) had a shorter overall survival than those with low expression of circSEPT9, N0–1 stage and TNM stage I (Fig. 3g). Cox proportional hazard model was used to evaluate the prognostic value of circSEPT9. It was found that high expression of circSEPT9 was an independent predictor of poor prognosis in TNBC patients (HR = 3.042, *P* = 0.012) (Table 3). In short, circSEPT9 could serve as a tumor promoter and high expression of circSEPT9 might predict a poor prognosis for patients with TNBC.

**Table 2:** Correlation between circSEPT9 expression and clinicopathological features in 80 TNBC patients (cohort 2)

Characteristic		All cases	circSEPT9		Chi-square	*P* value
			low	high		
All cases		80	37	43		
Age	< 53	36	14	22	1.427	0.232
	≥53	44	23	21		
Grade	II	40	20	20	0.453	0.501
	III	40	17	23		
T stage	T1	29	19	10	6.793	0.009**
	T2–3	51	18	33		
N stage	N0–1	56	29	27	2.301	0.129
	N2–3	24	8	16		
TNM stage	I	18	14	4	9.287	0.002**
	II/III	62	23	39		

**P < 0.01

**Table 3:** Univariate and multivariate Cox regression analysis of circSEPT9 and survival in patients with TNBC

Clinical variables	Univariate analysis		P	Multivariate analysis		P
	HR	95% CI		HR	95% CI	
Age (≥53 vs. < 53)	0.854	0.417–1.750	0.667			
Grade (II vs. III)	0.882	0.429–1.812	0.732			
T stage (T1 vs. T2/3)	1.110	0.528–2.334	0.783			
N stage (N0–1 vs. N2–3)	3.110	1.518–6.370	0.002**	2.274	1.062–4.871	0.035*
TNM stage (I vs. II/III)	3.428	1.038–11.317	0.043*	1.787	0.497–6.433	0.374
CircSEPT9 (low vs. high)	3.749	1.599–8.785	0.002**	3.042	1.278–7.240	0.012*

*Abbreviations*: *HR* Hazard ratio, *CI* Confidence interval, * *P* < 0.05,**P < 0.01

### circSEPT9 enhances proliferation, migration and invasion of TNBC cells

**Fig. 4:**
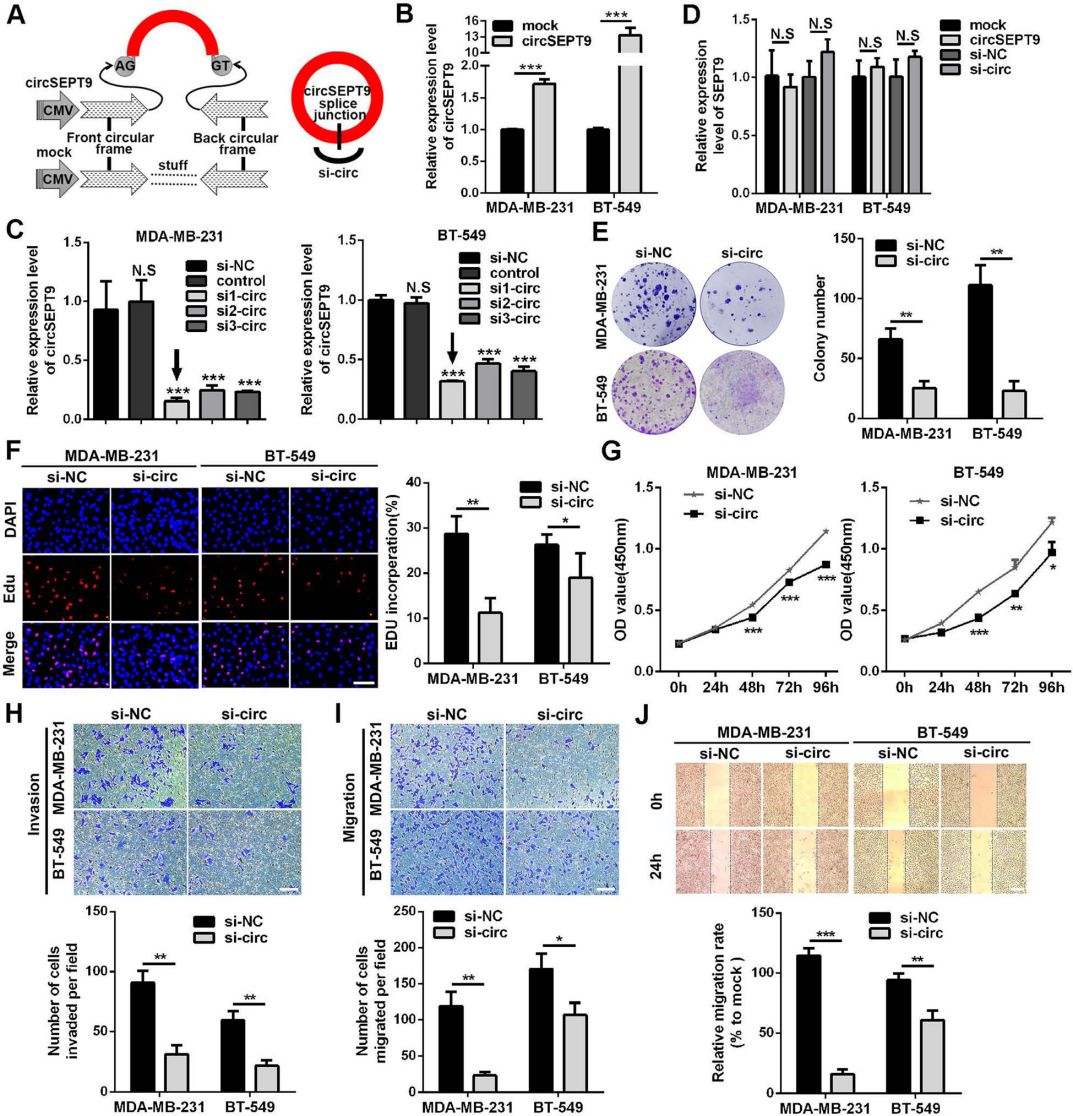
Silencing of circSEPT9 decreases proliferation, migration and invasion of TNBC cells. **a** The schematic illustration of circSEPT9 overexpression vector and si-circSEPT9 was shown. **b**-**d** Relative expression of circSEPT9 and SEPT9 mRNA was measured by qRT-PCR in TNBC cells transfected with circSEPT9 overexpression plasmid and si-circSEPT9. **e** Cell proliferation ability was evaluated by colony formation. **f** EdU assay of TNBC cells was performed to evaluate cell proliferation (magnification, × 100, Scale bar, 100 μm). **g** The growth curves of cells were measured by using CCK-8 assay. **h** Transwell invasion assay were used to assess the invasion abilities of TNBC cells (magnification, × 100, Scale bar, 100 μm). **i** and **j** The migration abilities of TNBC cells were measured by transwell migration (magnification, × 100, Scale bar, 100 μm) and wound healing (magnification, × 50, Scale bar, 100 μm) assays. The data are presented as the mean ± SD, *P < 0.05, **P < 0.01, ***P < 0.001

To probe the potential biological function of circSEPT9 in TNBC, we constructed overexpression vector of circSEPT9 and designed three siRNAs targeting the junction sites of circSEPT9 (Fig. 4a). The qRT-PCR results showed that the expression of circSEPT9 was significantly up-regulated or down-regulated in TNBC cells transfected with the indicated vectors or siRNA segments respectively (Fig. 4b and c). Among the three siRNAs, si1-circ had the highest silencing efficiency in TNBC cells, therefore, it was selected for further study. However, we found that both overexpression and knockdown of circSEPT9 had no effect on the level of linear transcript SEPT9 with specific primers for linear SEPT9 by qRT-PCR (Fig. 4d). The colony formation, CCK-8 and EdU assays were used to detect cell viability. The results showed that knockdown of circSEPT9 notably suppressed the proliferation ability of TNBC cells (Fig. 4e-g), whereas overexpression of circSEPT9 led to an increase in cell viability (Additional file 2: Figure S2a-c). Similarly, we found that down-regulation of circSEPT9 inhibited the migratory and invasive capabilities of TNBC cells by wound healing and transwell assays (Fig. 4h-j), while up-regulation of circSEPT9 revealed an opposite effect (Additional file 2: Figure S2d-f). These data suggest that circSEPT9 exerts an oncogenic role in the TNBC cells.

### Down-regulation of circSEPT9 induces cell cycle arrest, apoptosis and autophagy in TNBC cells

**Fig. 5:**
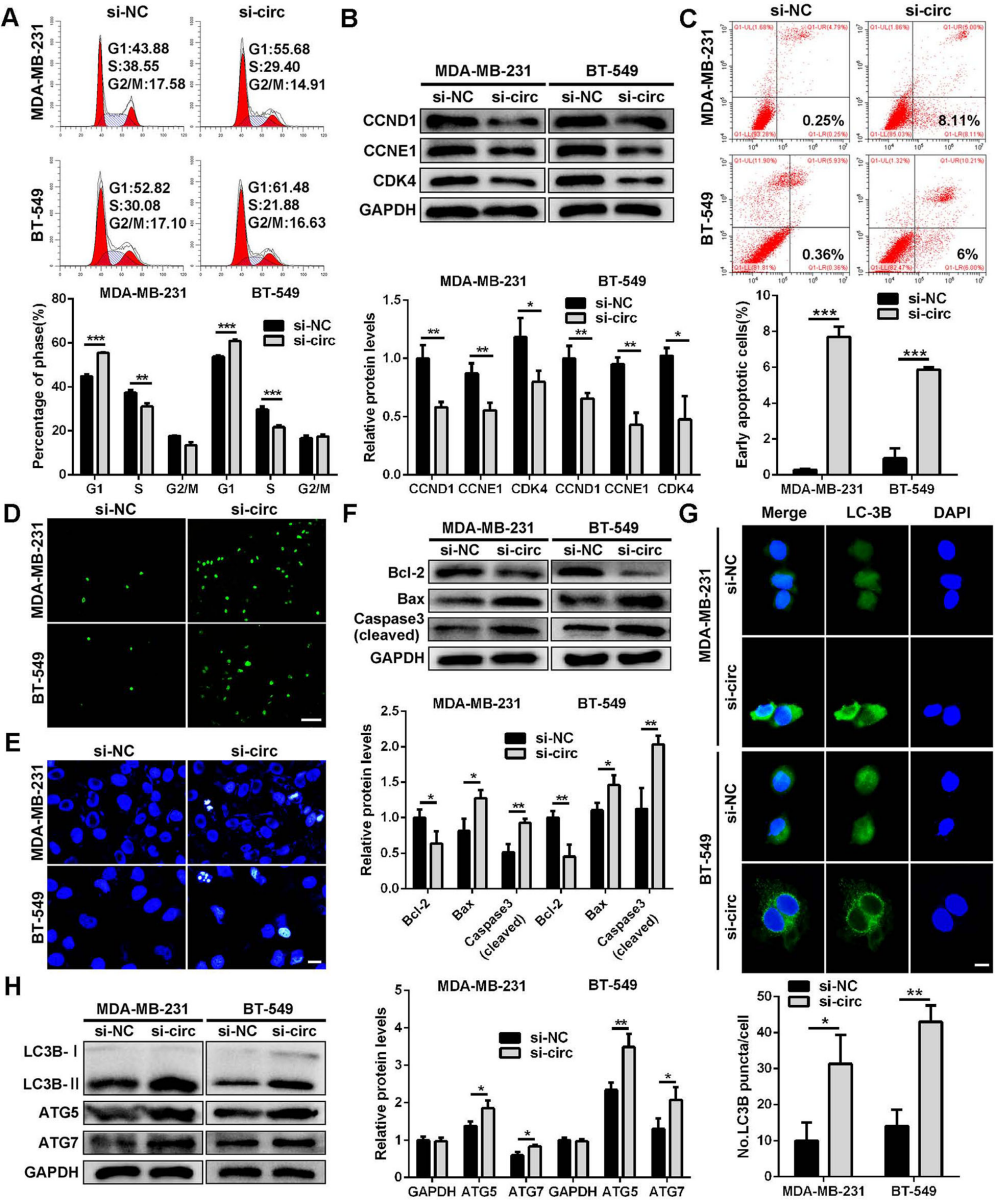
Knockdown of circSEPT9 induces cell cycle arrest, apoptosis and autophagy of TNBC cells. **a** Cell cycle analysis was performed using flow cytometry in TNBC cells transfected with si-circSEPT9. **b** Western blot was conducted to determine the expression of the cell cycle-related proteins in TNBC cells after transfection with si-circSEPT9. **c** Apoptosis rate was tested using flow cytometry after down-regulation of circSEPT9. **d** Apoptotic cells were observed with TUNEL method (magnification, × 100, scale bar, 100 μm). **e** Morphological features of apoptosis were revealed with Hoechst 33342 staining (magnification, × 200, scale bar, 20 μm). **f** Apoptosis-related protein levels were monitored by western blot. **g** The endogenous LC-3B puncta formation was assessed by IF analysis and the total number of LC3B puncta per cell was counted (magnification, × 400, Scale bar, 20 μm). **h** The levels of autophagy-related proteins were detected by western blot. The data are presented as the mean ± SD, *P < 0.05, **P < 0.01, ***P < 0.001

In order to further explore the role of circSEPT9 in TNBC progression, cell cycle, apoptosis and autophagy were investigated after circSEPT9 knockdown in vitro. The cell cycle analysis showed that more TNBC cells were distributed in G1 phase and less in S phase after silencing circSEPT9, which suggested that TNBC cells were arrested at G1 phase by si-circSEPT9 (Fig. 5a). Moreover, western blot assay found that down-regulation of circSEPT9 decreased the expression of cell cycle-related proteins, which could block cell cycle progression of TNBC cells (Fig. 5b). Next, we assessed the effect of circSEPT9 knockdown on apoptosis of TNBC cells. Flow cytometry analysis with Annexin V/PI double staining showed that the apoptotic rates of TNBC cells in si-circSEPT9 group were significantly higher than those of the control group cells (Fig. 5c). Furthermore, TUNEL assay indicated that down-regulation of circSEPT9 significantly increased the number of TUNEL-positive cells compared with control groups (Fig. 5d). Hoechst33342 staining found that TNBC cells in si-circSEPT9 group revealed typical apoptotic morphology characteristics such as nuclear shrinkage, apoptotic body and nuclear fragmentation, whereas no obvious apoptotic features were observed in the control group (Fig. 5e). In addition, the expressions of apoptosis-related proteins was detected by western blotting. The results showed that the expressions of activated (cleaved) caspase-3 and proapoptotic protein Bax were markedly enhanced in TNBC cells transfected with si-circSEPT9, while down-regulation of circSEPT9 led to a decrease of level of Bcl-2 (Fig. 5f). Subsequently, we investigated the effect of circSEPT9 knockdown on autophagy of TNBC cells. Immunofluorescence assay displayed that depletion of circSEPT9 induced LC3-II punctuation and accumulation of autophagosomes. The total number of LC3-II puncta per cell in si-circSEPT9 group was significantly higher than that in control group (Fig. 5g). The autophagy marker LC3 conversion from LC3-I to LC3-II was obviously increased and the levels of autophagy-related proteins ATG5 and ATG7 were significantly enhanced after knockdown of circSEPT9 by western blot (Fig. 5h). Taken together, these results suggest that circSEPT9 might regulate cell cycle, apoptosis and autophagy of TNBC cells.

### circSEPT9 directly binds to miR-637 and suppresses miR-637 activity

**Fig. 6:**
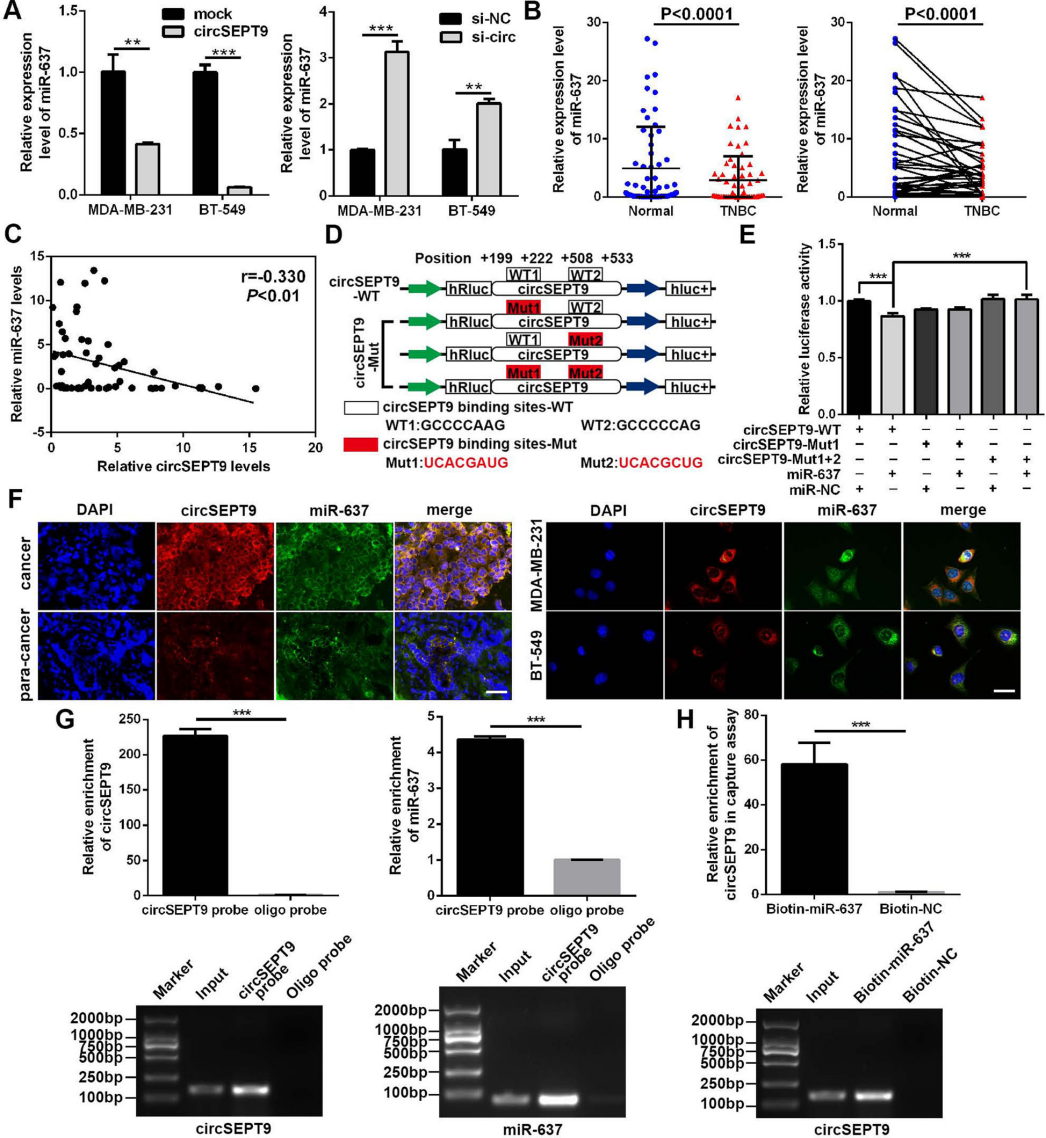
circSEPT9 functions as a sponge for miR-637. **a** The relative expression of miR-637 was determined in TNBC cells after transfection with circSEPT9 expression vector and si-circSEPT9 by qRT-PCR. **b** The relative expression of miR-637 was detected in 60 paired TNBC tissues and adjacent normal tissues by qRT-PCR. **c** An inverse correlation was shown between miR-637 and circSEPT9 expression in 60 TNBC tissues using pearson correlation analysis. **d** Schematic diagram of circSEPT9 luciferase reporter vectors carrying wild-type (WT) or mutant (Mut) miR-637 binding sites was displayed. **e** The relative luciferase activities were measured in 293 T cells co-transfected with circSEPT9-WT or circSEPT9-Mut and miR-637 mimics or miR-NC by luciferase reporter assay. **f** The co-localization of circSEPT9 and miR-637 was observed in TNBC tissues (magnification, × 100, Scale bar, 50 μm) and cells (magnification, × 200, Scale bar, 50 μm) by FISH assay. **g** RNA pull-down with a biotin-labeled circSEPT9 probe was implemented in MDA-MB-231 cells, followed by qRT-PCR and RT-PCR to test the enrichment of circSEPT9 and miR-637. **h** RNA pull-down with a biotin-labeled miR-637 probe was conducted in MDA-MB-231 cells, the content of circSEPT9 was detected by qRT-PCR and RT-PCR. The data are presented as the mean ± SD, **P < 0.01, ***P < 0.001

To probe the underlying molecular mechanism of circSEPT9, firstly, bioinformatic analysis was implemented to predict the possible binding sites of circSEPT9 and miR-637 with TargetScan database (http://www.targetscan.org) and miRanda database (http://www.microrna.org). The data showed that circSEPT9 contains two conserved miR-637 target sites, which suggested that circSEPT9 could act as a miRNA sponge in TNBC. Next, qRT-PCR assays were conducted to investigate the impact of circSEPT9 on the expression of miR-637. The results displayed that overexpression or knockdown of circSEPT9 resulted in down-regulation or up-regulation of miR-637 in TNBC cells, respectively (Fig. 6a). The expression of miR-637 was then determined in 60 TNBC tissues and matched normal tissues. The results showed that the expression of miR-637 in TNBC was notably lower than that in matched adjacent non-tumor tissues (Fig. 6b). Pearson correlation analysis revealed that the expression of circSEPT9 was reversely correlated with the level of miR-637 in TNBC tissues (Fig. 6c). Subsequently, dual-luciferase reporter assays were executed to measure the binding between circSEPT9 and miR-637. The wild and mutant dual-luciferase reporter plasmids of circSEPT9 were constructed (Fig. 6d). The data indicated that miR-637 mimics obviously reduced the luciferase activity of circSEPT9-WT luciferase reporter but not that of mutants (Fig. 6e), which suggest that circSEPT9 might directly combine with miR-637. Furthermore, we investigated the subcellular colocalization of circSEPT9 and miR-637 in TNBC cells and tissues by FISH assay. We found that circSEPT9 was co-localized with miR-637 in the cytoplasm (Fig. 6f). In order to further confirm the binding of circSEPT9 with miR-637, biotinylated circSEPT9 probe and biotin-labeled miR-637 mimics were used to conduct RNA pull down experiment. The results showed that circSEPT9 and miR-637 were abundantly pulled down by biotin-coupled circSEPT9 probe rather than oligo probe in circSEPT9-overexpressing MDA-MB-231 cells (Fig. 6g) and compared with the control group, the enrichment of circSEPT9 was significantly increased in the biotin-labeled miR-637 group (Fig. 6h). Together, these data suggest that circSEPT9 could directly combine with miR-637 and act as a sponge of miR-637.

### MiR-637 reverses the tumor promoting-effects of circSEPT9 in TNBC cells

**Fig. 7:**
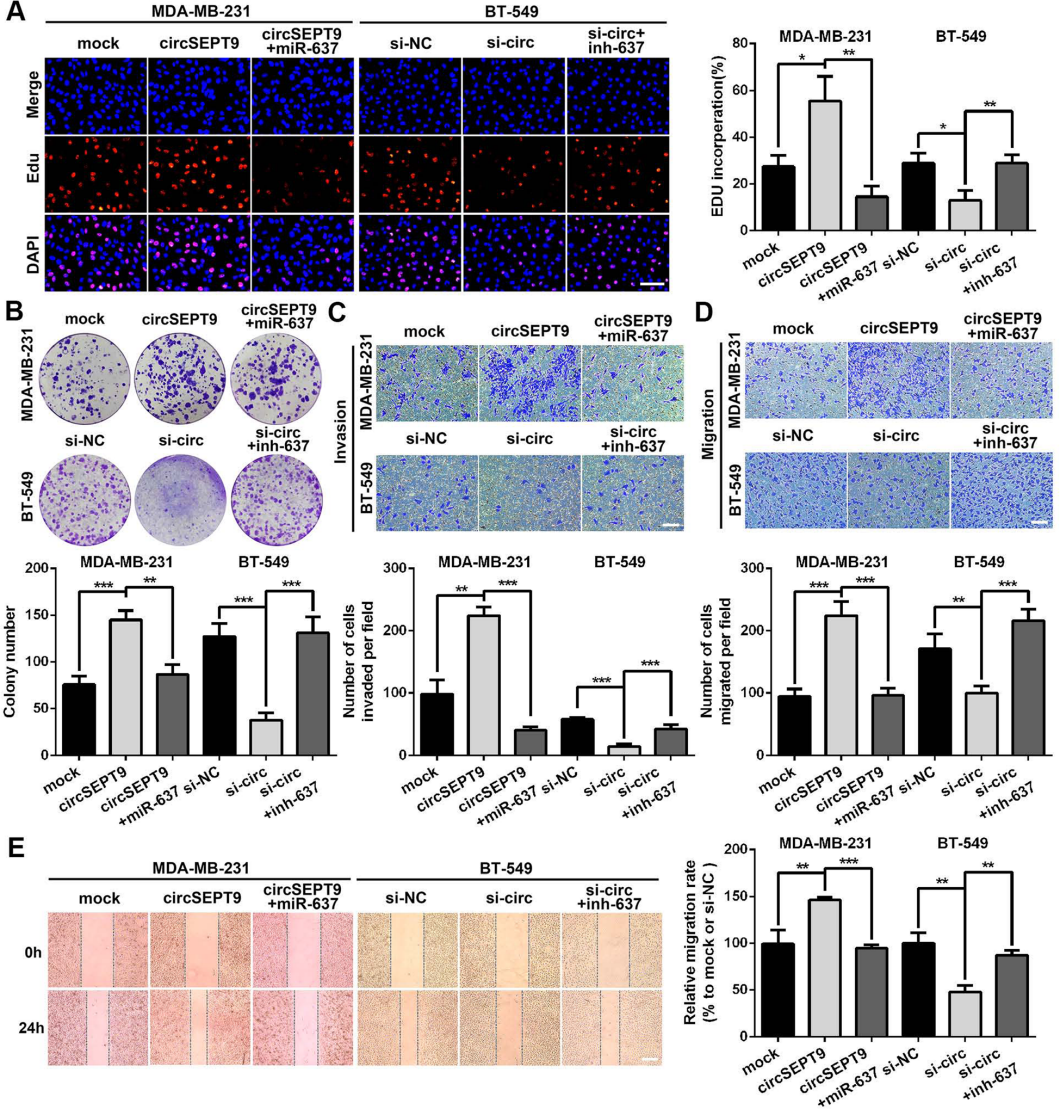
miR-637 could partially reverse the oncogenic effects of circSEPT9 on proliferation invasion and migration in TNBC cells. **a** and **b** The cell viability of TNBC cells were detected after transfection or co-transfection with indicated vectors, si-RNAs, miRNAs or inhibitors by EdU (magnification, × 200, Scale bar, 100 μm) and colony formation assays, respectively. **c**-**e** The invasion and migration abilities of TNBC cells transfected or co-transfected with indicated vectors, siRNAs, miRNA mimics or inhibitors were monitored by transwell invasion (magnification, × 100, Scale bar, 100 μm), transwell migration (magnification, × 100, Scale bar, 100 μm) and wound healing (magnification, × 50, Scale bar, 100 μm) assays, respectively. The data are presented as the mean ± SD, *P < 0.05, **P < 0.01, ***P < 0.001

To further investigate the functional interaction between circSEPT9 and miR-637 in TNBC cells, several rescue experiments were carried out by co-transfection of miR-637 mimics or miR-637 inhibitors and circSEPT9 vector or si-circSEPT9. The results revealed that ectopic expression of miR-637 significantly attenuated the proliferation, migration and invasion-promoting effects induced by up-regulation of circSEPT9 in TNBC cells, while miR-637 inhibitors could counteract the inhibitory roles of circSEPT9 knockdown in proliferation, migration and invasion of TNBC cells by EdU, colony formation, wound healing and transwell assays (Fig. 7a-e). Collectively, these experiments demonstrated that circSEPT9 could serve as a ceRNA to promote the tumorigenesis and development of TNBC.

### LIF is a direct target of miR-637 and activates LIF-STAT3 pathway by through circSEPT9/miR-637/LIF axis

**Fig. 8:**
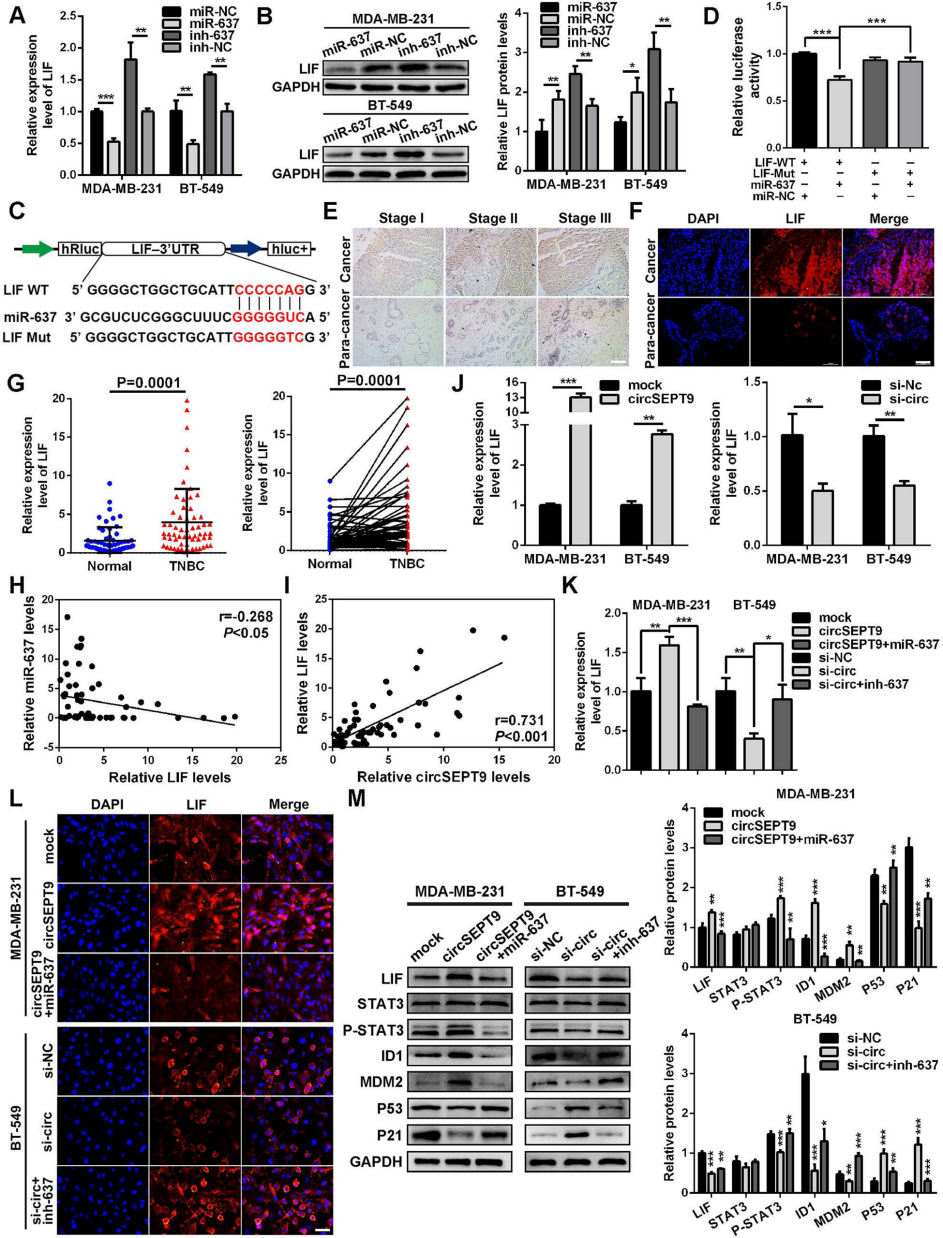
LIF is directly targeted by miR-637 and circSEPT9 stimulates LIF-STAT3 pathway by sponging miR-637 in TNBC. **a** and **b** The relative expression of LIF were detected after transfection with miR-637 and miR-637 inhibitor with qRT-PCR and western blot. **c** Schematic illustration of LIF 3’UTR wide type (WT) and mutant (Mut) luciferase reporter vectors were shown. **d** The luciferase reporter assays showed that miR-637 directly bind to the 3′-UTR of LIF and inhibit luciferase activity. **e** and **f** The relative expression of LIF was assessed by IHC (magnification, × 200, Scale bar, 100 μm) and IF (magnification, × 100, Scale bar, 100 μm) assays. **g** The relative expression of LIF was determined in 60 paired TNBC tissues and adjacent normal tissues with qRT-PCR. **h** and **i** Pearson correlation analyses of LIF expression with miR-637 or circSEPT9 were shown in 60 TNBC tissues. **j** The relative expression of LIF in TNBC cells after transfection with circSEPT9 expression vector and si-circSEPT9 by qRT-PCR. **k** The relative expression of LIF after transfection or co-transfection with indicated vectors, siRNAs, miRNA mimics or inhibitors by qRT-PCR. **l** IF analysis was applied to detect the protein expression of LIF in TNBC cells transfected or co-transfected with indicated vectors, siRNAs, miRNA mimics or inhibitors (magnification, × 200, Scale bar, 50 μm). **m** The relative protein levels of LIF and downstream LIF-STAT3 pathway-related molecules were measured in TNBC cells after transfection or co-transfection with indicated vectors, siRNAs, miRNA mimics or inhibitors by western blot. The data are presented as the mean ± SD, *P < 0.05, **P < 0.01, ***P < 0.001

To further identify the underlying mechanism, we performed bioinformatics analysis using the Targetscan (http://www.targetscan.org), miRanda (http://www.microrna.org) as well as Findtar online programs (http://bio.sz.tsinghua.edu.cn). The data showed that LIF contains conserved target sites of miR-637. The qRT-PCR and western blot analysis exhibited that the expression of LIF was remarkably reduced at both mRNA and protein levels in TNBC cells transfected with miR-637 mimics, while LIF expression was notably enhanced in TNBC cells transfected with miR-637 inhibitors (Fig. 8a and b). Next, wild and mutant dual-luciferase reporter plasmids containing LIF 3′-untranslated regions (UTRs) were constructed (Fig. 8c),we found that transfection of miR-637 mimics could evidently decreased the activity of a luciferase reporter carrying wild-type 3′-UTR of LIF but not that of mutant 3′-UTR of LIF by dual luciferase reporter assay (Fig. 8d). Next, the expression of LIF was evaluated with immunohistochemistry (IHC) and immunofluorescence (IF). The results displayed that LIF was up-regulated in TNBC tissues compared with matched paracancerous tissues and the expression of LIF in stage III TNBC tissues was stronger than that in stage Iand stage IITNBC tissues (Fig. 8e and f). Furthermore, the expression of LIF was examined in the 60 pairs of TNBC and adjacent non-cancerous tissues by qRT-PCR. It was found that LIF was significantly up-regulated in TNBC tissues (Fig. 8g). Pearson correlation analysis showed that the expression of LIF was negatively correlated with the level of miR-637 (Fig. 8h). These data suggest that miR-637 might directly target LIF. In addition, correlation analysis also revealed that the expression of LIF was positively correlated with the expression of circSEPT9 (Fig. 8i). Subsequently, the expression of LIF was further evaluated in TNBC cells after the overexpression or knockdown of circSEPT9 with qRT-PCR, the results showed that up-regulation or down-regulation of circSEPT9 led to an increase or decrease in the expression of LIF (Fig. 8j). Moreover, we detect whether circSEPT9 could regulate the expression of LIF in TNBC cells, the qRT-PCR and IF assays showed that overexpression of circSEPT9 remarkably increased the expressions of LIF and knockdown of circSEPT9 obviously decreased the levels of LIF. Additionally, miR-637 mimics or inhibitors could reverse the increase or decrease of LIF induced by circSEPT9 overexpression or knockdown (Fig. 8k and l). Further investigations showed that overexpression of circSEPT9 increased the expression of LIF, P-STAT3, ID1 and MDM2 and simultaneously decreased P53 and P21, whereas knockdown of circSEPT9 exerted the opposite role. Importantly, these effects could be abolished by miR-637 mimics or inhibitors, respectively (Fig. 8m). In summary, these results demonstrated that circSEPT9 could function as sponge of miR-637 to promote TNBC progression via activating LIF-STAT3 pathway.

### CircSEPT9 accelerates the growth and metastasis of xenograft tumors in vivo

**Fig. 9:**
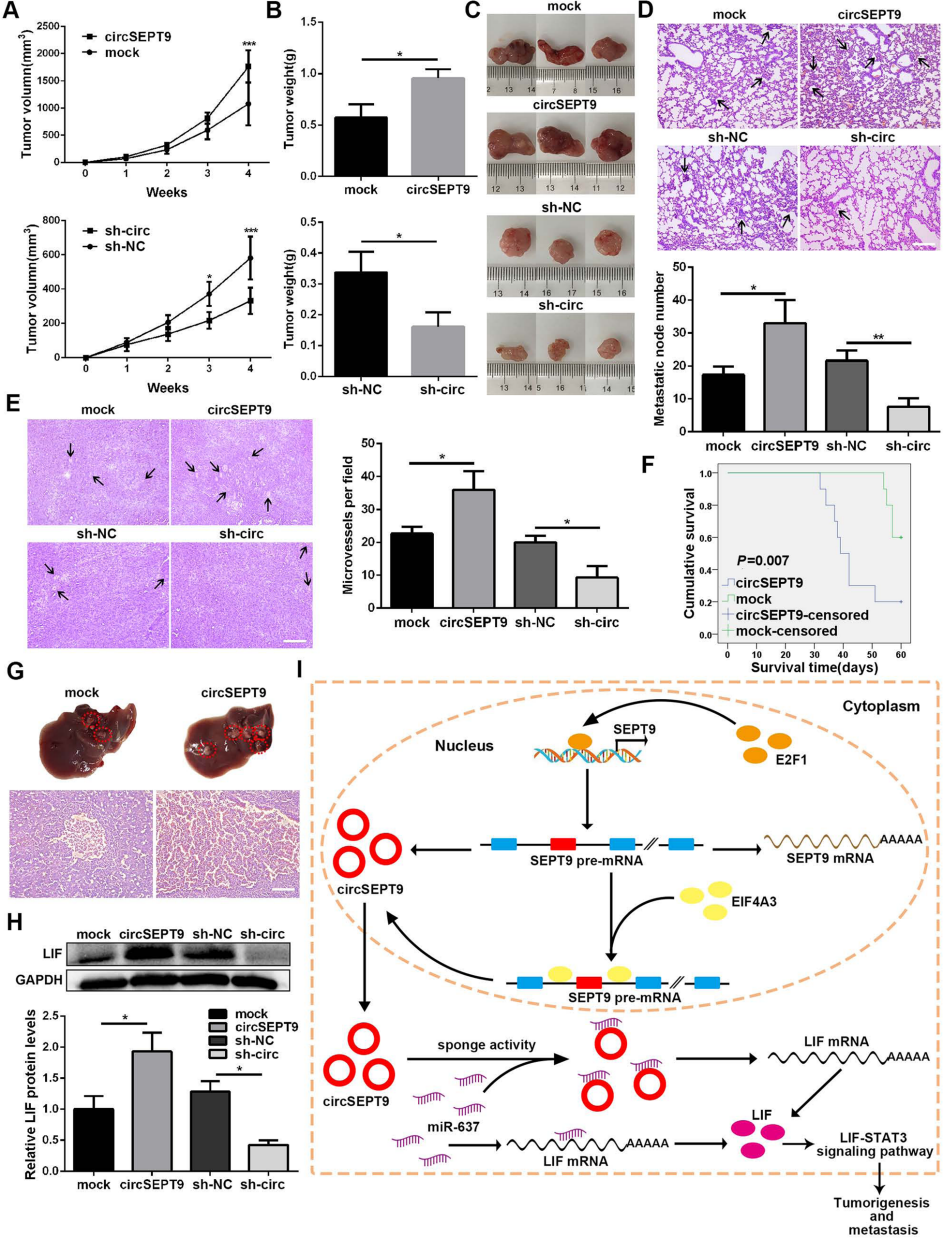
circSEPT9 promotes oncogenesis and metastasis of TNBC cells. **a** The tumor volumes were measured once a week and the growth curves were drawn. **b** Tumor weight of was analyzed. **c** The representative images of xenograft tumor in each group were displayed (*n* = 3). **d** and **e** H&E staining of the lungs (magnification, × 100, Scale bar, 100 μm) and tumors (magnification, × 200, Scale bar, 100 μm) were showed. Metastatic nodules of the lungs and microvessels of the tumors were indicated by arrows. **f** The survival curve was drawn by Kaplan-Meier method for the nude mice injected with MDA-MB-231 cells transfected with circSEPT9 overexpressing or mock vector. **g** The representative images of liver metastasis in mice inoculated with MDA-MB-231 cells for 60 days were taken (magnification, × 200, Scale bar, 100 μm). **h** Western blot analysis was conducted to detect the protein level of LIF in xenograft tumor tissues. **i** Schematic diagram illustrates the mechanism of circSEPT9 mediated by E2F1 and EIF4A3 to promote TNBC tumorigenesis and progression through circSEPT9/miR-637/LIF axis. The data are presented as the mean ± SD, *P < 0.05, **P < 0.01, ***P < 0.001

To evaluate whether circSEPT9 affected in vivo tumor growth and metastasis, the human TNBC xenograft model was established, MDA-MB-231 cells with stable overexpression or knockdown of circSEPT9 and their respective controls were subcutaneously injected into female nude mice. The results showed that the volume and weight of tumors in overexpression circSEPT9 group were strikingly higher than those in the control group, while circSEPT9 knockdown remarkably inhibited tumor growth (Fig. 9a-c). Moreover, the up-regulation of circSEPT9 obviously promoted spontaneous lung metastasis with a higher number of metastatic nodules, while knockdown of circSEPT9 significantly suppressed pulmonary metastasis with fewer invasive tumor cells compared with control group (Fig. 9d). In addition, the overexpression of circSEPT9 could markedly facilitate tumor angiogenesis, whereas circSEPT9 silencing significantly reduced the density of microvessels in the tumors (Fig. 9e). Kaplan-Meier survival curve displayed that the nude mice injected with circSEPT9-overexpressing MDA-MB-231 cells had a lower overall survival compared to control group (Fig. 9f). The number and size of metastatic nodules in the liver of mice from circSEPT9 overexpression group for survival experiment were higher and larger than these from the control group (Fig. 9g). Subsequently, we further explored the impact of circSEPT9 on the expression of target gene LIF and LIF-STAT3 pathway-related proteins in vivo*.* The results of western blotting showed that the expression of LIF was remarkably increased or decreased in circSEPT9 overexpressing or silencing xenograft tumor tissues compared with control groups respectively (Fig. 9h). IHC analysis further revealed that overexpressing circSEPT9 could up-regulate the expressions of LIF, P-STAT3, ID1 and MDM2 as well as decrease of the levels of P53 and P21 in tumor tissues of mice, while circSEPT9 silencing caused the opposite effects (Additional file 2: Figure S3). These results were consistent with assays in vitro, suggesting that circSEPT9 could promote tumorigenesis and metastasis of TNBC through activating LIF-STAT3 pathway.

## Discussion

Although more than 90% of the human genome is actively transcribed, only 1–2% of the genomic sequences encode proteins, while most of the sequences may contribute to the expression of non-coding RNA (ncRNAs) [21]. In the past two decades, the abnormal expression and/or function of noncoding RNAs in tumorigenesis and tumor development has become one of the most important scientific discoveries. Compared with known non-coding RNA microRNA and LncRNA, circRNA is a new hotspot in the field of non-coding RNA research [22]. In recent years, the role of circRNAs in oncogenesis and cancer progression has caused wide attention. Due to cell/tissue-specific and stage-specific expression and unique molecular structure, circRNAs might have regulatory functions in many biological processes and are better diagnostic markers or therapeutic targets for cancer than linear transcripts [23]. However, the expression and role of most circRNAs in TNBC development are still largely unclear.

Here, we investigated the circRNA expression profile in TNBC tissues and paracancerous tissues from four patients using RNA-seq. We focused on the role and potential mechanism of a new circRNA termed circSEPT9, which was remarkably up-regulated in TNBC and was significantly associated with the clinical stage and poor prognosis of TNBC patients. Functionally, we found that knockdown of circSEPT9 could significantly suppress cell proliferation and invasion, induce cell apoptosis and autophagy as well as inhibit oncogenesis and metastasis in vivo, while the over-expression of circSEPT9 displayed the opposite effects. Mechanistically, we demonstrated that E2F1 and EIF4A3 might facilitate the biogenesis of circSEPT9. Furthermore, circSEPT9 could function as a sponge for miR-637 to relieve the inhibitory effect on LIF, which activated LIF/Stat3 signaling pathway and led to the pathogenesis and development of TNBC. Our data suggest that circSEPT9 could play an oncogenic role in the progression of TNBC and would be a new diagnostic and prognostic marker or therapeutical target for TNBC patients.

Accumulating data indicates that the circRNAs play an important regulatory role in gene expression at the post-transcriptional level. CircRNAs might function as a new member of the ceRNA family to regulate the expression of oncogene or tumor suppressor gene via sponging miRNAs. The balance between shared miRNAs and targeted ceRNAs is critical for ceRNA activity and disruption of the balance between ceRNAs and miRNAs might contribute to tumor development. For example, it was reported that circRNA circ_0000190 inhibited the development of multiple myeloma by regulating miR-767-5p/MAPK4 pathway [24]. In addition, circPRMT5 induced epithelial-mesenchymal transition to promote metastasis of bladder carcinoma via sponging miR-30c [25]. Moreover, Han etal. demonstrated that the expression of circMTO1 was significantly down-regulated in hepatocellular carcinoma tissues and survival time of hepatocellular carcinoma patients with low expression of circMTO1 was shortened. CircMTO1 suppressed hepatocellular carcinoma progression to promote p21 expression by serving as of miR-9 sponge [26]. In the present study, we found that circSEPT9 has two binding sites for miR-637 by bioinformatics analysis. FISH results indicated that circSEPT9 and miR-637 share the same subcellular localization in TNBC cells. Next, dual-luciferase reporter and biotin-coupled probe pull-down assays further verified that circSEPT9 could directly bind to miR-637. These results showed that circSEPT9 might function as a competing endogenous RNA to promote oncogenesis and progression of TNBC.

Based on the ceRNA hypothesis, circRNA serves as a ceRNA to regulate miRNA target gene expression. We found that LIF and circSEPT9 are co-overexpressed in TNBC. Furthermore, bioinformatics analysis showed that LIF is a latent target of miR-637 using miRcode and TargetScan. Subsequently, the dual-luciferase reporter assay verified that miR-637 might directly bind to the 3′-UTR of LIF. Besides, miR-637 mimics led to a significant decrease in both the mRNA and protein levels of LIF, whereas miR-637 inhibitor displayed an opposite effect. We further found that miR-637 was obviously down-regulated in TNBC tissues. It was reported that miR-637 as a tumor suppressor gene could inhibit the growth of breast cancer cells [27]. Que. et al. demonstrated that decreased miRNA-637 was a poor prognostic marker and promoted the growth, migration and invasion of glioma cells by directly targeting AKT1 [28]. MiR-637 directly targets leukemia inhibitory factor (LIF), which suppresses the tumorigenesis of hepatocellular carcinoma by interfering with the transcription factor Stat3 signaling pathway [29]. Our experimental results support these findings. LIF is a cytokine of the interleukin 6 family and regulates many important biological functions. Studies have shown that LIF overexpresses in many tumors including breast cancer and promotes growth and metastasis of tumors [30, 31]. LIF was also found to be an important negative regulator of p53. LIF could negatively regulate the level of p53 protein and function by Stat3/ID1/MDM2 in colon cancer cells. Overexpression of LIF is an important mechanism to weaken the function of p53 [32]. Consistent with these studies, we demonstrated that LIF was markedly up-regulated in TNBC tissues and positively correlated with clinical stage. More importantly, we revealed that up-regulation of circSEPT9 could enhance the expression of LIF and suppress the level of p53 as well as activate LIF/Stat3 signaling pathway, whereas circSEPT9 knockdown showed a contrary effect. Subsequently, these impacts might be reversed by miR-637 mimics or inhibitors, respectively. These results further confirm our hypothesis that circSEPT9 serves as a ceRNA for miR-637 to enhance LIF expression and activate LIF/Stat3 signaling pathway in TNBC progression.

RNA binding protein EIF4A3 is the core component of exon junction complex (EJC), which is considered as an important regulator of post-transcriptional regulation processes including mRNA splicing, transport, translation, and surveillance [33]. In the present study, we discovered that EIF4A3 can combine with the upstream and down stream region of circSEPT9 pre-mRNA and modulate its expression. Thus, we believe that EIF4A3 could mediates the biogenesis of circSEPT9. Some RNA-binding proteins play an important role in the formation of circRNAs. The research found that hundreds of circRNAs were regulated in the process of epithelial-mesenchymal transition (EMT) and more than one-third of the generation of circRNAs was dynamically regulated by the alternative splicing factor Quaking (QKI) [34]. Wang et.al reported that EIF4A3 could bind to the MMP9 mRNA transcript, facilitate circMMP9 cyclization and enhance circMMP9 expression in glioblastoma multiforme [35]. Our result support the finding. Further evidence is needed to prove that EIF4A3 can participate in the biogenesis of circSEPT9. Moreover, the bioinformatics analysis predicted that E2F1 could bind to SEPT9 gene promoter, which was verified by ChIP and luciferase reporter assays. Subsequently, we showed that overexpression of E2F1 could increase the level of circSEPT9 and knockdown of E2F1 inhibited the expression of circSEPT9, suggesting that E2F1 might promote SEPT9 gene transcription. E2F1 is a transcription factor of many important proteins that drive cells through the G1/S transition and S-phase [36]. At present, there are few studies on the regulatory mechanism of circRNA by upstream transcription factors. Meng et al. demonstrated that that transcription factor Twist1 promoted Cul2 transcription through binding to the Cul2 promoter and upregulated Cul2 circRNA expression but represseed Cul2 expression [37]. However, detailed mechanisms in the regulatory process need further elucidation.

## Conclusions

Taken together, we identify a novel circRNA, termed circSEPT9, which could play an oncogenic role in TNBC and is associated with poor prognosis. We found that circSEPT9 might sponge miR-637 to regulate LIF expression and activate LIF/Stat3 signaling pathway, leading to oncogenesis and progression of TNBC. Furthermore, we also demonstrate that E2F1 and EIF4A3 could mediate the biogenesis of circSEPT9, but the detailed mechanism needs further study. Our data suggest that circSEPT9 can act as a promising prognostic biomarker and new therapeutical target for TNBC.

## Supplementary information

**Additional file 1: Table S1.** Sequences of siRNAs and shRNAs used in this study. **Table S2.** Primer sequences used in qRT-PCR and PCR analysis. **Table S3,** RNA-seq data of dysregulated circRNAs in TNBC tissues.

**Additional file 2: Figure S1.** Relative expression levels of E2F1 and EIF4A3 were determined by qRT-PCR. (a) Relative expression of E2F1 expression were evaluated in TNBC cells transfected with E2F1 overexpression plasmids or siRNA. (b) The relative expression of EIF4A3 was detected in TNBC cells transfected with EIF4A3 overexpression or knockdown plasmids. The data are presented as the mean ± SD, **P < 0.01, ***P < 0.001. **Figure S2.** Overexpression of circSEPT9 increased proliferation, migration and invasion of TNBC cells by colony formation, EdU, CCK-8, wound healing and transwell assays. (**a**) Cell proliferation ability was evaluated by colony formation. (**b)** EdU assay of TNBC cells was performed to evaluate cell proliferation (magnification, × 100, Scale bar, 100 μm). (**c**) The growth curves of cells were measured by using CCK-8 assay. (**d**) Transwell invasion assay were used to assess the invasion abilities of TNBC cells (magnification, × 100, Scale bar, 100 μm). (**e and f**) The migration abilities of TNBC cells were measured by transwell migration (magnification, × 100, Scale bar, 100 μm) and wound healing (magnification, × 50, Scale bar, 100 μm) assays. The data are presented as the mean ± SD, *P < 0.05, **P < 0.01, ***P < 0.001. **Figure S3.** The expressions of LIF and LIF-STAT3 signal pathway related molecules were detected in tumor tissues of mice by IHC (magnification, × 200, Scale bar, 100 μm).

## Data Availability

The datasets used and analyzed during the current study are available from the corresponding author on reasonable request.

## Abbreviations

CircRNA: Circular RNA
TNBC: Triple-negative breast cancer
RNA-seq: RNA sequencing
qRT-PCR: Real-time quantitative polymerase chain reaction
ceRNA: competing endogenous RNA
ER: Estrogen receptor
PR: Progesterone receptor
HER2: Human epidermal growth factor receptor 2
miRNA: microRNA
TCGA: The Cancer Genome Atlas
TMA: Tissue microarray
ISH: In situ hybridization
IHC: Immunohistochemistry
IF: Immunofluorescence
FISH: Fluorescence in situ hybridization
3’UTR: 3′ -untranslated region
RIP: RNA immunoprecipitation
ROC curve: Receiver operating characteristic curve
RNAi: RNA interfere
