# Supplementary material for: The circRNA circSEPT9 mediated by E2F1 and EIF4A3 facilitates the carcinogenesis and development of triple-negative breast cancer
Source: Mol Cancer. 2020 Apr 7;19:73. doi: 10.1186/s12943-020-01183-9 (PMC7137343; doi:10.1186/s12943-020-01183-9)
Supplement: Supplementary file 1 — Additional file 1: Table S1. Sequences of siRNAs and shRNAs used in this study. Table S2. Primer sequences used in qRT-PCR and PCR analysis. Table S3, RNA-seq data of dysregulated circRNAs in TNBC tissues. [file 12943_2020_1183_MOESM1_ESM.doc]

**Table S1 Sequences of siRNAs and shRNAs used in this study**

| **Definition** | **sequences** |
| --- | --- |
| si1-circ | 5'- GGAGCCAGGAGGCCTTGAA-3' |
| si2-circ | 5'- CAGGAGGCCTTGAAAAGAT-3' |
| si3-circ | 5'- GGAGGCCTTGAAAAGATCT-3' |
| si-NC | 5'- TTCTCCGAACGTGTCACGT-3' |
| si1-E2F1 | 5’-CCUGGAAACUGACCAUCAGTT-3’ |
| si2-E2F1 | 5’-GCUGGACCACCUGAUGAAUTT-3’ |
| si3-E2F1 | 5’-GGACUCUUCGGAGAACUUUTT-3’ |
| si-NC | 5’-UUCUCCGAACGUGUCACGUTT-3’ |
| sh1-EIF4A3 | 5'- GCAATCCAGCAACGAGCAATC-3' |
| sh2-EIF4A3 | 5'- GTGAAACGTGATGAATTGACT-3' |
| sh3-EIF4A3 | 5'- GCCATTAACTTTGTAAAGAAT-3' |
| sh-NC | 5'- GTTCTCCGAACGTGTCACGT-3' |

**Table S2 Primer sequences used in RT-qPCR and PCR analysis**

| **Gene** | **Primer sequences** |
| --- | --- |
| GAPDH | F: 5'- GAAGGTGAAGGTCGGAGTC-3' |
|  | R: 5'- GAAGATGGTGATGGGATTTC-3' |
| U6 | F: 5'- GCTTCGGCAGCACATATACTAAAAT-3' |
|  | R: 5'- CGCTTCACGAATTTGCGTGTCAT-3' |
|  | RT: 5'- CGCTTCACGAATTTGCGTGTCAT-3' |
| 18S rRNA | F: 5'- ACACGGACAGGATTGACAGA-3' |
|  | R: 5'- GGACATCTAAGGGCATCACA-3' |
| SEPT9 | F: 5'- GAGAAGTTCCGGGAGATGATC-3' |
|  | R: 5'- GCAGGTAGGCAAACTCACAG-3' |
| CircSEPT9 | F: 5'- ttggagaattcagagcctg-3' |
|  | R: 5'- aagatcttttcaaggcctcc-3' |
| CircSEPT9(PCR) | F: 5'- ttggagaattcagagcctg-3' |
|  | R: 5'- gtcctggaatttctgggtggagc-3' |
| hsa-mir-637 | RT: 5'-CTCAACTGGTGTCGTGGAGTCGGCAATTCAGTTGAGACGCAGAG-3' |
|  | F: 5'- ACACTCCAGCTGGGACTGGGGGCTTTCGGGCT-3' |
|  | R: 5'- TGGTGTCGTGGAGTCG-3' |
| LIF | F: 5'-ATACGCCACCCATGTCACAA-3' |
|  | R: 5'-CCCTGGGCTGTGTAATAGAGAA-3' |
| Cyclin D1 | F: 5'-GCTGCGAAGTGGAAACCATC-3' |
|  | R: 5'-CCTCCTTCTGCACACATTTGAA-3' |
| Cyclin E1 | F: 5'-GCCGCAGTATCCCCAGCAAA-3' |
|  | R: 5'-TCGCACCACTGATACCCTGA-3' |
| CDK4 | F: 5'-GTCGGCTTCAGAGTTTCCAC-3' |
|  | R: 5'-TGCAGTCCACATATGCAACA-3' |
| EIF4A3 | F: 5'-CGCGGACTCTGACATATGGCGACCACGGCCACGATG-3' |
|  | R: 5'-TCCCGCAGGCCCATGGTGTCG-3' |
| E2F1 | F: 5'-CCGTGGACTCTTCGGAGAAC-3' |
|  | R: 5'-ATCCCACCTACGGTCTCCTC-3' |

**Table S3 RNA-seq data of dysregulated circRNAs in TNBC tissues**

| circRNA_ID | Gene | hsa_circbase_id | Foldchange | PValue | FDR | 1Ca | 1P | 2Ca | 2P | 3Ca | 3P | 4Ca | 4P |
| --- | --- | --- | --- | --- | --- | --- | --- | --- | --- | --- | --- | --- | --- |
| chr2_228356263_228389631_+ | AGFG1 | hsa_circ_0058514 | 15.0416577 | 0.004483243 | 0.076823035 | 0.98677586 | 0 | 0.233853174 | 0.045140708 | 0.600503441 | 0 | 0.270922064 | 0 |
| chr1_26584088_26586293_+ | CEP85 | hsa_circ_0000033 | 14.65375829 | 0.000316975 | 0.018290686 | 0.84580788 | 0 | 0.701559523 | 0.022570354 | 0.600503441 | 0 | 0.361229418 | 0.024571492 |
| chr17_75398141_75398785_+ | SEPT9 | hsa_circ_0005320 | 12.24548199 | 0.000446117 | 0.019609758 | 0.7048399 | 0 | 0.701559523 | 0.022570354 | 2.251887902 | 0.112115518 | 0.361229418 | 0 |
| chr19_18285850_18286507_+ | IFI30 | hsa_circ_0005571 | 11.49891436 | 0.002587087 | 0.054490519 | 0.28193596 | 0.03770986 | 0.467706348 | 0.045140708 | 0.600503441 | 0 | 1.354610318 | 0 |
| chr4_1902353_1936989_+ | WHSC1 | hsa_circ_0001387 | 11.49544901 | 0.001261199 | 0.036958625 | 0.98677586 | 0 | 0.935412697 | 0.045140708 | 0.600503441 | 0 | 0.180614709 | 0.024571492 |
| chr21_38792601_38794168_+ | DYRK1A | hsa_circ_0001190 | 9.570639836 | 0.005448879 | 0.084992626 | 1.12774384 | 0 | 0.467706348 | 0.022570354 | 0.750629301 | 0.044846207 | 0 | 0 |
| chr4_1219148_1235307_- | CTBP1 | hsa_circ_0001386 | 8.815742269 | 0.000939932 | 0.032212569 | 0.98677586 | 0.03770986 | 1.403119045 | 0.090281415 | 0.45037758 | 0.022423104 | 0.451536773 | 0 |
| chr5_138979957_138994551_+ | UBE2D2 | hsa_circ_0005728 | 8.331292236 | 0.008432622 | 0.104605905 | 0.7048399 | 0 | 0.233853174 | 0.022570354 | 0.45037758 | 0.044846207 | 0.361229418 | 0 |
| chr4_178274462_178281831_+ | NEIL3 | hsa_circ_0001460 | 8.239315007 | 0.016203101 | 0.150287476 | 0.7048399 | 0 | 0.233853174 | 0.112851769 | 1.050881021 | 0.067269311 | 1.354610318 | 0 |
| chr19_18650181_18650530_- | FKBP8 | hsa_circ_0000915 | 8.11448268 | 0.007478826 | 0.099416403 | 0.14096798 | 0.03770986 | 1.169265871 | 0 | 0.45037758 | 0 | 0.632151482 | 0.073714477 |
| chr1_117944808_117957453_+ | MAN1A2 | hsa_circ_0000117 | 8.047950984 | 0.013176227 | 0.132548914 | 0.56387192 | 0 | 0.467706348 | 0.067711061 | 1.201006881 | 0.022423104 | 0 | 0 |
| chr6_mann_hap4_3851080_3999480_- | not_annotated | NA | 7.948703026 | 1.32E-22 | 2.68E-19 | 84.58078804 | 0.603357761 | 0 | 3.724108376 | 67.85688879 | 2.399272092 | 51.20427 | 1.793718928 |
| chr7_44684926_44687358_+ | OGDH | hsa_circ_0003340 | 7.231669883 | 0.026289529 | 0.194715855 | 0.14096798 | 0 | 1.403119045 | 0.022570354 | 0.30025172 | 0.112115518 | 0.451536773 | 0 |
| chr4_41015600_41016415_- | APBB2 | hsa_circ_0005991 | 7.165093495 | 0.048337589 | 0.278457562 | 0.42290394 | 0 | 0.467706348 | 0.157992477 | 0.45037758 | 0 | 0.090307355 | 0 |
| chr5_177649356_177652430_- | PHYKPL | hsa_circ_0075256 | 7.131207919 | 0.044045764 | 0.262800893 | 0.42290394 | 0 | 0.467706348 | 0.067711061 | 0.30025172 | 0 | 0 | 0 |
| chr5_179136874_179147561_+ | CANX | hsa_circ_0002051 | 7.100868419 | 0.014470237 | 0.139994347 | 0.56387192 | 0 | 1.636972219 | 0.022570354 | 0.45037758 | 0.156961726 | 0.541844127 | 0.024571492 |
| chr1_169446393_169446995_- | SLC19A2 | hsa_circ_0015164 | 7.079432004 | 0.011486079 | 0.122236067 | 1.409679801 | 0.07541972 | 0.467706348 | 0.090281415 | 0.600503441 | 0 | 0.090307355 | 0 |
| chr7_716866_751164_- | PRKAR1B | hsa_circ_0008039 | 6.89338563 | 0.001945303 | 0.046275335 | 1.268711821 | 0.11312958 | 1.169265871 | 0.067711061 | 0.600503441 | 0.022423104 | 0.180614709 | 0 |
| chr10_125798031_125806240_- | CHST15 | hsa_circ_0000264 | 6.181419833 | 0.012389877 | 0.12807019 | 0.56387192 | 0 | 0.233853174 | 0.067711061 | 0.900755161 | 0.022423104 | 0.361229418 | 0.024571492 |
| chrX_130883334_130928494_- | FIRRE | hsa_circ_0001944 | 6.058456193 | 0.018926103 | 0.162845023 | 0.42290394 | 0.15083944 | 3.975503961 | 0.135422123 | 0.600503441 | 0.134538622 | 3.61229418 | 0 |
| chr6_4891947_4892613_+ | CDYL | hsa_circ_0008285 | 5.944549203 | 0.03347513 | 0.229446484 | 1.12774384 | 0.11312958 | 0 | 0.067711061 | 1.951636182 | 0.022423104 | 0.270922064 | 0 |
| chr1_67423742_67428843_+ | MIER1 | NA | 5.764414713 | 0.01036844 | 0.115192225 | 0.42290394 | 0.03770986 | 0.935412697 | 0.067711061 | 0.750629301 | 0.022423104 | 0 | 0 |
| chr7_148716084_148718239_- | PDIA4 | hsa_circ_0001766 | 5.73387722 | 0.034516931 | 0.232150474 | 0.28193596 | 0 | 0.233853174 | 0 | 1.351132741 | 0.044846207 | 0.090307355 | 0.049142984 |
| chr19_3623686_3624160_- | CACTIN | hsa_circ_0002661 | 5.711199793 | 0.038747785 | 0.244837567 | 0.84580788 | 0 | 0.233853174 | 0.045140708 | 0 | 0 | 0.632151482 | 0.049142984 |
| chr10_126370176_126370948_- | FAM53B | hsa_circ_0000267 | 5.60718894 | 0.020295209 | 0.169574021 | 2.114519701 | 0 | 0.467706348 | 0.293414599 | 1.050881021 | 0.112115518 | 1.80614709 | 0.049142984 |
| chr19_13039156_13039661_- | FARSA | hsa_circ_0000896 | 5.596712961 | 0.025508681 | 0.192397315 | 1.409679801 | 0.07541972 | 0.233853174 | 0.112851769 | 1.951636182 | 0 | 0.722458836 | 0.098285969 |
| chr19_17212470_17213367_+ | MYO9B | hsa_circ_0000907 | 5.594439798 | 0.001302779 | 0.03714573 | 1.832583741 | 0.15083944 | 0.701559523 | 0.157992477 | 2.251887902 | 0.112115518 | 1.264302963 | 0.024571492 |
| chr19_3660964_3661999_- | PIP5K1C | hsa_circ_0000871 | 5.433418366 | 0.018887649 | 0.162845023 | 0 | 0 | 0.467706348 | 0.045140708 | 0.600503441 | 0.022423104 | 0.541844127 | 0.024571492 |
| chrX_1404671_1409402_+ | CSF2RA | NA | 5.381840831 | 0.031763746 | 0.221469982 | 0.42290394 | 0 | 0.701559523 | 0.18056283 | 0.45037758 | 0 | 0.270922064 | 0.024571492 |
| chr13_43528084_43544806_- | EPSTI1 | hsa_circ_0000479 | 5.326016273 | 0.01185201 | 0.124816482 | 2.114519701 | 0.26396902 | 0 | 0.067711061 | 4.954153385 | 0.067269311 | 1.264302963 | 0.073714477 |
| chr8_25265499_25266456_+ | DOCK5 | hsa_circ_0007618 | 5.252360282 | 0.020799943 | 0.171663202 | 0.42290394 | 0.07541972 | 0.701559523 | 0.090281415 | 0.45037758 | 0 | 0.180614709 | 0 |
| chr8_102570647_102571040_+ | GRHL2 | hsa_circ_0085173 | 5.169109682 | 0.011628941 | 0.123108475 | 0.84580788 | 0.1885493 | 0 | 0 | 1.201006881 | 0.022423104 | 1.715839736 | 0.122857461 |
| chr12_19615444_19626289_+ | AEBP2 | hsa_circ_0006420 | 5.159491567 | 0.03541349 | 0.236323683 | 0.56387192 | 0.3770986 | 0.467706348 | 0.022570354 | 0.750629301 | 0 | 0.632151482 | 0.024571492 |
| chr4_1902353_1932497_+ | WHSC1 | hsa_circ_0002688 | 5.035083517 | 0.01841252 | 0.160749493 | 0.98677586 | 0.03770986 | 0.467706348 | 0.067711061 | 0.30025172 | 0.044846207 | 0.270922064 | 0 |
| chr5_14316622_14336836_+ | TRIO | hsa_circ_0005260 | 5.014824139 | 0.030077957 | 0.215925722 | 0.56387192 | 0.33938874 | 1.169265871 | 0.045140708 | 0.750629301 | 0 | 0.361229418 | 0.024571492 |
| chr16_84773915_84779279_+ | USP10 | hsa_circ_0003026 | 4.941952454 | 0.033776514 | 0.22977712 | 0.28193596 | 0 | 1.169265871 | 0.022570354 | 0.30025172 | 0.089692415 | 0.270922064 | 0.024571492 |
| chr12_12672796_12674397_- | DUSP16 | hsa_circ_0003855 | 4.806814419 | 0.04235112 | 0.259046425 | 0.7048399 | 0.11312958 | 0 | 0.045140708 | 1.201006881 | 0.022423104 | 0.270922064 | 0 |
| chr2_89476005_89544456_- | not_annotated | NA | 4.742810566 | 0.032344361 | 0.22397362 | 0.84580788 | 0.1885493 | 0 | 0 | 1.201006881 | 0.022423104 | 0.270922064 | 0.024571492 |
| chr3_169854207_169896726_- | PHC3 | hsa_circ_0002622 | 4.710801666 | 0.015517578 | 0.146619357 | 0.28193596 | 0 | 0.467706348 | 0.045140708 | 1.050881021 | 0.044846207 | 0.270922064 | 0.049142984 |
| chr1_155691308_155695810_+ | DAP3 | hsa_circ_0014613 | 4.582896519 | 0.042790062 | 0.259046425 | 0.56387192 | 0 | 0 | 0.022570354 | 0.750629301 | 0.022423104 | 0.541844127 | 0.098285969 |
| chr14_102506573_102507010_+ | DYNC1H1 | hsa_circ_0002398 | 4.254396606 | 0.024947973 | 0.19035774 | 0.28193596 | 0 | 0.701559523 | 0.090281415 | 0.750629301 | 0.022423104 | 0.541844127 | 0.098285969 |
| chr16_80718435_80719026_- | CDYL2 | hsa_circ_0004087 | 4.252267945 | 0.042627204 | 0.259046425 | 0.42290394 | 0.15083944 | 0.233853174 | 0 | 0.750629301 | 0.067269311 | 0.270922064 | 0 |
| chr1_171492360_171502100_+ | PRRC2C | hsa_circ_0015211 | 4.231395261 | 0.036204497 | 0.239233636 | 1.12774384 | 0.26396902 | 0 | 0.022570354 | 1.050881021 | 0.022423104 | 0.541844127 | 0.024571492 |
| chr4_6925100_6925838_+ | TBC1D14 | hsa_circ_0001394 | 4.193663674 | 0.037429978 | 0.242727879 | 0.7048399 | 0.03770986 | 0.467706348 | 0.045140708 | 0.30025172 | 0.067269311 | 0.180614709 | 0 |
| chr2_122260743_122287901_- | CLASP1 | hsa_circ_0002374 | 3.926872144 | 0.034106403 | 0.230645978 | 0.42290394 | 0.07541972 | 0.701559523 | 0.135422123 | 0.900755161 | 0.089692415 | 0.632151482 | 0 |
| chr17_20107646_20109225_+ | SPECC1 | hsa_circ_0000745 | 3.282767696 | 0.004056371 | 0.071321585 | 5.497751222 | 1.018166221 | 13.09577776 | 1.060806628 | 4.804027525 | 0.650270006 | 4.154138307 | 0.442286859 |
| chr7_99621042_99621930_+ | ZKSCAN1 | hsa_circ_0001727 | 2.034087504 | 0.033150188 | 0.227992112 | 17.33906155 | 2.601980342 | 5.612476181 | 2.821294225 | 22.66900488 | 2.26473347 | 6.592436879 | 1.449718038 |
| chr4_153332455_153333681_- | FBXW7 | hsa_circ_0001451 | -2.148295602 | 0.04557918 | 0.267910179 | 8.317110824 | 3.318467683 | 1.870825394 | 5.665158803 | 13.36120155 | 5.247006257 | 3.521986826 | 5.061727387 |
| chr1_58971732_59004982_- | OMA1 | hsa_circ_0002316 | -2.704967513 | 0.042181038 | 0.259046425 | 1.409679801 | 2.828239502 | 0.701559523 | 0.902814152 | 2.552139623 | 1.524771049 | 1.083688254 | 1.056574163 |
| chr9_86293356_86301070_- | UBQLN1 | hsa_circ_0087357 | -2.890326811 | 0.048015362 | 0.277391603 | 0.28193596 | 0.49022818 | 0.233853174 | 0.293414599 | 0.30025172 | 0.291500348 | 0.270922064 | 0.270286414 |
| chr10_32197100_32199491_- | ARHGAP12 | hsa_circ_0000231 | -2.9925617 | 0.039345987 | 0.247321646 | 2.396455661 | 2.262591602 | 2.338531742 | 1.218799105 | 3.603020644 | 2.825311062 | 0.361229418 | 3.046865029 |
| chr12_121220458_121222396_- | SPPL3 | hsa_circ_0003472 | -3.075665094 | 0.04447766 | 0.264511261 | 0.84580788 | 0.980456361 | 0.467706348 | 1.399361935 | 0.900755161 | 1.009039665 | 1.354610318 | 1.032002671 |
| chr14_31596991_31602881_- | HECTD1 | hsa_circ_0002301 | -3.238598553 | 0.031223666 | 0.220367329 | 0 | 0.49022818 | 0.701559523 | 0.383696015 | 0.30025172 | 0.381192762 | 0.361229418 | 0.319429398 |
| chr22_46096162_46136418_+ | ATXN10 | hsa_circ_0001246 | -3.290037339 | 0.038281437 | 0.243412153 | 0.28193596 | 0.30167888 | 0.935412697 | 0.586829199 | 0.600503441 | 0.67269311 | 0 | 0.270286414 |
| chr12_69983265_69987393_+ | CCT2 | hsa_circ_0002940 | -3.329570696 | 0.043409874 | 0.260538177 | 0.14096798 | 0.5656479 | 0.467706348 | 0.722251321 | 0.15012586 | 0.403615866 | 0.632151482 | 0.22114343 |
| chr9_86294690_86301070_- | UBQLN1 | hsa_circ_0008207 | -3.330716446 | 0.030855859 | 0.218914202 | 0.42290394 | 0.867326781 | 0 | 0.112851769 | 0.750629301 | 0.515731384 | 0.090307355 | 0.172000445 |
| chr11_33307959_33309057_+ | HIPK3 | hsa_circ_0000284 | -3.419804382 | 3.75E-07 | 0.000189709 | 11.84131033 | 20.21248498 | 19.64366663 | 6.65825437 | 12.76069811 | 14.19382462 | 1.80614709 | 12.70346145 |
| chr11_77330651_77336863_- | CLNS1A | hsa_circ_0023694 | -3.566104353 | 0.045497587 | 0.267910179 | 0.14096798 | 0.22625916 | 0.701559523 | 0.383696015 | 0 | 0.381192762 | 0.180614709 | 0.22114343 |
| chr4_103225474_103236987_- | SLC39A8 | hsa_circ_0002782 | -3.597596086 | 0.029610088 | 0.213827135 | 0.28193596 | 0.26396902 | 0 | 0.270844246 | 0.45037758 | 0.403615866 | 0.180614709 | 0.368572383 |
| chr18_19345733_19399607_+ | MIB1 | hsa_circ_0000836 | -3.606914726 | 0.03011427 | 0.215925722 | 0.56387192 | 0.33938874 | 0.233853174 | 0.541688491 | 0.45037758 | 0.448462073 | 0.090307355 | 0.491429843 |
| chr8_141828376_141900868_- | PTK2 | hsa_circ_0005982 | -3.663828408 | 0.022422352 | 0.178496048 | 0.56387192 | 2.111752162 | 1.870825394 | 1.12851769 | 0.45037758 | 0.829654836 | 0.451536773 | 0.56514432 |
| chr15_32819014_32825569_- | WHAMMP1 | NA | -3.66493331 | 0.025442353 | 0.192397315 | 0.42290394 | 0.754197201 | 0 | 0.248273892 | 1.351132741 | 0.829654836 | 0.180614709 | 0.417715367 |
| chr9_5968019_5988545_- | KIAA2026 | NA | -3.719978512 | 0.048866192 | 0.279907761 | 0 | 0.641067621 | 0.233853174 | 0.18056283 | 0.15012586 | 0.448462073 | 1.083688254 | 0.442286859 |
| chr16_47531310_47549512_+ | PHKB | hsa_circ_0000698 | -3.787297187 | 0.010178438 | 0.114337788 | 0.42290394 | 1.621523981 | 3.507797613 | 2.099042903 | 1.050881021 | 1.636886567 | 0.632151482 | 1.081145656 |
| chr8_141856359_141900868_- | PTK2 | hsa_circ_0003221 | -3.80254919 | 0.013020711 | 0.131639385 | 0.42290394 | 0.754197201 | 0.701559523 | 0.677110614 | 0.30025172 | 0.42603897 | 0.270922064 | 0.638858796 |
| chr10_128768966_128926028_+ | DOCK1 | hsa_circ_0020397 | -3.864675034 | 0.036197728 | 0.239233636 | 0.14096798 | 0.11312958 | 0 | 0.789962383 | 0.30025172 | 0.560577592 | 0.632151482 | 0.393143875 |
| chr4_91229395_91234198_+ | CCSER1 | NA | -3.874899436 | 0.023628519 | 0.183757177 | 0 | 0.1885493 | 0.467706348 | 0.293414599 | 0.15012586 | 0.448462073 | 0.361229418 | 0.393143875 |
| chr7_140476712_140508795_- | BRAF | hsa_circ_0007178 | -3.904810306 | 0.047614317 | 0.275862891 | 0 | 0.49022818 | 0.467706348 | 0.293414599 | 0.600503441 | 0.313923451 | 0 | 0.122857461 |
| chr8_17601113_17613470_- | MTUS1 | hsa_circ_0083444 | -3.969041836 | 0.047401458 | 0.275825565 | 0.56387192 | 0.942746501 | 0 | 0.767392029 | 0.45037758 | 0.986616561 | 1.535225027 | 0.614287304 |
| chr5_122881111_122893258_+ | CSNK1G3 | hsa_circ_0001522 | -4.001985031 | 0.001075854 | 0.035086722 | 1.12774384 | 4.072664884 | 2.104678568 | 1.263939813 | 4.503775805 | 3.834350726 | 1.264302963 | 4.496583067 |
| chr16_30740287_30740893_+ | SRCAP | hsa_circ_0006127 | -4.026396635 | 0.02597994 | 0.193296499 | 0.28193596 | 0.30167888 | 0 | 0.406266368 | 0.45037758 | 0.291500348 | 0.090307355 | 0.319429398 |
| chr12_46319925_46322642_- | SCAF11 | hsa_circ_0025967 | -4.077625944 | 0.009373263 | 0.11083472 | 0.14096798 | 0.754197201 | 0.233853174 | 0.225703538 | 0.45037758 | 0.42603897 | 0.180614709 | 0.319429398 |
| chr1_219366424_219414650_+ | LYPLAL1 | hsa_circ_0004417 | -4.117298704 | 0.016005292 | 0.149513903 | 0.28193596 | 0.5656479 | 0 | 0.157992477 | 0.750629301 | 0.448462073 | 0.090307355 | 0.417715367 |
| chr17_60111148_60112969_- | MED13 | hsa_circ_0004273 | -4.158434818 | 0.040044389 | 0.249137707 | 0.56387192 | 0.641067621 | 0 | 0 | 0.600503441 | 0.42603897 | 0 | 0.638858796 |
| chr15_65266940_65275931_- | SPG21 | hsa_circ_0003526 | -4.160182277 | 0.04342303 | 0.260538177 | 0 | 0.603357761 | 0 | 0.270844246 | 0.600503441 | 0.269077244 | 0.180614709 | 0.147428953 |
| chr6_76412361_76412788_+ | SENP6 | hsa_circ_0001614 | -4.31324205 | 0.03790456 | 0.243234194 | 0.84580788 | 0.829616921 | 0 | 0.022570354 | 0.900755161 | 0.896924147 | 0 | 0.516001336 |
| chr5_65284463_65310553_+ | ERBB2IP | hsa_circ_0001493 | -4.314199677 | 0.017737733 | 0.158057516 | 0.28193596 | 0.22625916 | 0.467706348 | 0.338555307 | 0.30025172 | 0.493308281 | 0 | 0.540572828 |
| chr3_67546222_67559327_- | SUCLG2 | hsa_circ_0004276 | -4.390544266 | 0.019979194 | 0.169037575 | 0.84580788 | 0.603357761 | 0.233853174 | 0.406266368 | 0.45037758 | 0.695116214 | 0.090307355 | 0.786287749 |
| chr1_169947226_170001116_- | KIFAP3 | NA | -4.396243977 | 0.012444475 | 0.12807019 | 0.84580788 | 1.885493002 | 0.701559523 | 0.586829199 | 0.900755161 | 1.009039665 | 0.180614709 | 1.007431179 |
| chr15_41961026_41962156_+ | MGA | hsa_circ_0000591 | -4.453436677 | 0.049170857 | 0.280857269 | 0.7048399 | 1.998622582 | 1.636972219 | 0.564258845 | 0.900755161 | 0.829654836 | 0 | 1.228574609 |
| chr14_91947920_91952074_- | SMEK1 | hsa_circ_0003045 | -4.460934119 | 0.038614156 | 0.244758068 | 0 | 0.716487341 | 0.935412697 | 0.383696015 | 0.750629301 | 0.336346555 | 0 | 0.466858351 |
| chr4_139981478_139994721_- | ELF2 | hsa_circ_0007137 | -4.478092205 | 0.042463105 | 0.259046425 | 0.14096798 | 0.15083944 | 0 | 0.135422123 | 0 | 0.112115518 | 0.270922064 | 0.393143875 |
| chr7_80418622_80440017_- | SEMA3C | hsa_circ_0004365 | -4.525308845 | 0.005310993 | 0.084557703 | 1.12774384 | 1.357554961 | 1.169265871 | 2.166753964 | 0.900755161 | 1.457501738 | 0.270922064 | 0.90914521 |
| chr10_32832228_32873232_+ | CCDC7 | hsa_circ_0000233 | -4.562263953 | 0.023858425 | 0.184754903 | 0.28193596 | 1.206715521 | 0.233853174 | 0 | 0.45037758 | 0.515731384 | 0 | 0.319429398 |
| chr14_92264129_92268765_- | TC2N | hsa_circ_0032969 | -4.6020414 | 0.01356212 | 0.134911324 | 0.14096798 | 0.3770986 | 0 | 0.451407076 | 0.750629301 | 0.381192762 | 0.180614709 | 0.516001336 |
| chr10_86198268_86237420_+ | CCSER2 | hsa_circ_0018998 | -4.604675136 | 0.008582209 | 0.105812363 | 0.28193596 | 0.49022818 | 0 | 0.338555307 | 0.30025172 | 0.605423799 | 0.632151482 | 0.761716257 |
| chr1_28785596_28793046_+ | PHACTR4 | hsa_circ_0005406 | -4.627405834 | 0.039507859 | 0.247321646 | 0 | 0.22625916 | 0 | 0.18056283 | 0.15012586 | 0.470885177 | 0.632151482 | 0.270286414 |
| chr10_49609655_49618211_+ | MAPK8 | hsa_circ_0002968 | -4.63161601 | 0.020091631 | 0.169037575 | 0.56387192 | 1.583814121 | 0.467706348 | 0.112851769 | 0.30025172 | 0.493308281 | 0 | 0.466858351 |
| chr20_34304662_34313077_- | RBM39 | hsa_circ_0001147 | -4.658502532 | 0.030694242 | 0.218542914 | 0 | 0.15083944 | 0 | 0.361125661 | 0.30025172 | 0.269077244 | 0.180614709 | 0.172000445 |
| chr2_36623757_36669878_+ | CRIM1 | hsa_circ_0002346 | -4.81440765 | 0.007940089 | 0.101804733 | 0.14096798 | 0.867326781 | 0.701559523 | 0.83510309 | 0.30025172 | 0.852077939 | 0.451536773 | 0.442286859 |
| chr2_159165945_159201830_- | CCDC148 | hsa_circ_0056768 | -4.853933709 | 0.023939557 | 0.184754903 | 0.42290394 | 0.52793804 | 0 | 0.090281415 | 0.30025172 | 0.313923451 | 0 | 0.294857906 |
| chr16_53175091_53191453_+ | CHD9 | NA | -4.85703548 | 0.008259107 | 0.103803167 | 0.14096798 | 0.603357761 | 0 | 0.203133184 | 0.15012586 | 0.470885177 | 0.541844127 | 0.393143875 |
| chr2_168994598_169038600_- | STK39 | hsa_circ_0002029 | -4.866120759 | 0.042363822 | 0.259046425 | 0.14096798 | 0.15083944 | 0.467706348 | 0.248273892 | 0 | 0.224231037 | 0 | 0.22114343 |
| chr15_62299507_62306191_- | VPS13C | hsa_circ_0000607 | -4.879933635 | 0.024912722 | 0.19035774 | 0.28193596 | 0.5656479 | 0.233853174 | 0.067711061 | 0.900755161 | 1.076308976 | 0 | 0.810859242 |
| chr15_22835916_22849121_+ | TUBGCP5 | NA | -4.966861459 | 0.044872931 | 0.266079373 | 0 | 0.1885493 | 0.233853174 | 0.112851769 | 0.15012586 | 0.134538622 | 0 | 0.196571937 |
| chr1_200729967_200784772_+ | CAMSAP2 | hsa_circ_0015839 | -4.987424328 | 0.034962776 | 0.23408852 | 0.28193596 | 0.5656479 | 0 | 0.293414599 | 0.45037758 | 0.112115518 | 0 | 0.442286859 |
| chr3_43341246_43345284_+ | SNRK | hsa_circ_0004089 | -5.02746346 | 0.007320946 | 0.099416403 | 0.42290394 | 0.791907061 | 0 | 0.157992477 | 0.600503441 | 0.560577592 | 0 | 0.245714922 |
| chr22_38917613_38964294_- | DMC1 | hsa_circ_0001231 | -5.036938119 | 0.038012861 | 0.243234194 | 0 | 0.1885493 | 0 | 0.203133184 | 0.15012586 | 0.134538622 | 0.090307355 | 0.098285969 |
| chr5_176636637_176639196_+ | NSD1 | hsa_circ_0075161 | -5.122002209 | 0.013704395 | 0.134911324 | 0.42290394 | 0.641067621 | 0 | 0.248273892 | 0 | 0.224231037 | 0.180614709 | 0.22114343 |
| chr9_6880012_6893232_+ | KDM4C | hsa_circ_0001839 | -5.186610766 | 0.006189921 | 0.090695796 | 0.14096798 | 0.07541972 | 0.233853174 | 0.496547784 | 0.30025172 | 0.515731384 | 0.090307355 | 0.491429843 |
| chr3_56661065_56662642_- | FAM208A | hsa_circ_0001314 | -5.230386496 | 0.039455225 | 0.247321646 | 0.14096798 | 1.319845101 | 0 | 0 | 1.201006881 | 0.695116214 | 0 | 0.098285969 |
| chr2_239090706_239093928_- | ILKAP | hsa_circ_0001116 | -5.262936467 | 0.019980591 | 0.169037575 | 0.28193596 | 0.22625916 | 0 | 0.270844246 | 0.30025172 | 0.42603897 | 0 | 0.196571937 |
| chr18_19371335_19383975_+ | MIB1 | NA | -5.325897175 | 0.026825552 | 0.197960823 | 0 | 0.1885493 | 0 | 0.428836722 | 0.15012586 | 0.134538622 | 0.180614709 | 0.172000445 |
| chr18_60223436_60232329_+ | ZCCHC2 | NA | -5.327671047 | 0.031581454 | 0.220960902 | 0.14096798 | 0.26396902 | 0 | 0.090281415 | 0 | 0.089692415 | 0.090307355 | 0.172000445 |
| chr4_103635595_103647840_- | MANBA | hsa_circ_0001432 | -5.340546462 | 0.029493639 | 0.213749602 | 0.28193596 | 0.1885493 | 0 | 0.270844246 | 0.15012586 | 0.291500348 | 0 | 0.147428953 |
| chr2_30748453_30756180_+ | LCLAT1 | hsa_circ_0000987 | -5.387807258 | 0.032109385 | 0.22311057 | 0.14096798 | 0.45251832 | 0 | 0.18056283 | 0.15012586 | 0.089692415 | 0 | 0.073714477 |
| chr4_107092252_107133992_- | TBCK | hsa_circ_0007540 | -5.461864056 | 0.022175979 | 0.17793583 | 0 | 0.26396902 | 0.233853174 | 0.586829199 | 0 | 0.224231037 | 0.270922064 | 0.122857461 |
| chr8_116599228_116632287_- | TRPS1 | hsa_circ_0085361 | -5.466233775 | 0.033759662 | 0.22977712 | 0.14096798 | 0.11312958 | 0 | 0.383696015 | 0 | 0.134538622 | 0.090307355 | 0.122857461 |
| chr3_175165017_175189546_+ | NAALADL2 | hsa_circ_0068032 | -5.473636244 | 0.042703632 | 0.259046425 | 0.14096798 | 0.678777481 | 0 | 0.112851769 | 0.30025172 | 0.067269311 | 0 | 0.270286414 |
| chr12_120995085_120995485_+ | RNF10 | hsa_circ_0028899 | -5.520518773 | 0.009886137 | 0.112157328 | 0.28193596 | 0.30167888 | 0 | 0.203133184 | 0.15012586 | 0.336346555 | 0.090307355 | 0.368572383 |
| chr15_93467551_93499879_+ | CHD2 | hsa_circ_0036963 | -5.521236318 | 0.048599932 | 0.279173471 | 0 | 0.07541972 | 0 | 0.225703538 | 0 | 0.067269311 | 0.180614709 | 0.147428953 |
| chr17_29550462_29554624_+ | NF1 | hsa_circ_0003586 | -5.528914443 | 0.02258675 | 0.179099641 | 0.28193596 | 0.26396902 | 0 | 0.293414599 | 0.15012586 | 0.269077244 | 0 | 0.122857461 |
| chr8_99718695_99719539_- | STK3 | hsa_circ_0004592 | -5.545581834 | 0.000978073 | 0.032961064 | 1.268711821 | 3.092208523 | 0.701559523 | 1.12851769 | 0.900755161 | 1.569617256 | 0.361229418 | 1.376003562 |
| chr6_131247745_131277639_- | EPB41L2 | hsa_circ_0077837 | -5.620702841 | 0.005778596 | 0.087196424 | 0.14096798 | 0.942746501 | 0 | 0.677110614 | 0.600503441 | 0.515731384 | 0.451536773 | 0.56514432 |
| chr5_43295854_43297268_- | HMGCS1 | hsa_circ_0072391 | -5.777272411 | 0.007906968 | 0.101804733 | 0.14096798 | 1.470684541 | 0 | 0.248273892 | 0.15012586 | 0.089692415 | 0.180614709 | 0.196571937 |
| chr8_116616100_116635985_- | TRPS1 | hsa_circ_0085363 | -5.78849199 | 0.001170261 | 0.035852536 | 0.84580788 | 2.300301462 | 0.233853174 | 0.970525213 | 1.201006881 | 1.614463464 | 0.541844127 | 1.449718038 |
| chr1_32381496_32385259_- | PTP4A2 | hsa_circ_0007364 | -5.822448346 | 0.026002299 | 0.193296499 | 0 | 0.41480846 | 0.467706348 | 1.083376982 | 0.15012586 | 1.031462769 | 1.444917672 | 0.466858351 |
| chr2_95814630_95819004_- | ZNF514 | NA | -5.823453595 | 0.010239643 | 0.114389822 | 0.14096798 | 0.716487341 | 0.233853174 | 0.383696015 | 0 | 0.784808628 | 0.722458836 | 0.442286859 |
| chr1_219366424_219392054_+ | LYPLAL1 | hsa_circ_0004314 | -5.823664387 | 0.018116046 | 0.159263671 | 0 | 0.26396902 | 0 | 0.315984953 | 0.750629301 | 0.291500348 | 0.090307355 | 0.417715367 |
| chr18_51797730_51804225_+ | POLI | hsa_circ_0047719 | -5.840534713 | 0.017032947 | 0.154442233 | 0.28193596 | 0.52793804 | 0 | 0.293414599 | 0 | 0.067269311 | 0.090307355 | 0.172000445 |
| chr2_227729320_227732034_+ | RHBDD1 | hsa_circ_0058493 | -5.915438478 | 0.047286188 | 0.275825565 | 1.550647781 | 0.30167888 | 0 | 0.789962383 | 0 | 0.493308281 | 0.270922064 | 0.56514432 |
| chr2_58449077_58459247_- | FANCL | hsa_circ_0001009 | -5.937642709 | 0.018702483 | 0.162302236 | 0.14096798 | 1.018166221 | 0.233853174 | 0.090281415 | 0.900755161 | 0.583000695 | 0 | 0.933716702 |
| chrX_134679348_134690225_+ | DDX26B | NA | -5.978911644 | 0.003953936 | 0.070354868 | 0.28193596 | 0.49022818 | 0 | 0.18056283 | 0.600503441 | 0.695116214 | 0 | 0.34400089 |
| chr11_95825056_95826681_- | MAML2 | hsa_circ_0024085 | -6.070331162 | 0.007522606 | 0.099416403 | 0.14096798 | 0.905036641 | 0 | 0.789962383 | 0.600503441 | 0.941770354 | 0.812766191 | 0.663430289 |
| chr6_117019867_117026323_+ | KPNA5 | hsa_circ_0001639 | -6.085678788 | 0.021711504 | 0.17630788 | 0.14096798 | 0.1885493 | 0 | 0.135422123 | 0.15012586 | 0.201807933 | 0 | 0.196571937 |
| chr2_68717322_68772444_+ | APLF | hsa_circ_0001023 | -6.090570148 | 0.008250043 | 0.103803167 | 0.14096798 | 1.018166221 | 0 | 1.015665921 | 1.050881021 | 1.121155183 | 0.632151482 | 0.614287304 |
| chr4_151656410_151729550_- | LRBA | hsa_circ_0071174 | -6.133812664 | 0.017822509 | 0.158057516 | 0.14096798 | 0.41480846 | 0 | 0.045140708 | 0.30025172 | 0.291500348 | 0 | 0.245714922 |
| chr11_120916383_120930794_+ | TBCEL | hsa_circ_0003302 | -6.170495513 | 0.002753688 | 0.055473024 | 0.84580788 | 2.187171882 | 0.233853174 | 1.376791582 | 1.201006881 | 1.053885872 | 0.180614709 | 0.860002226 |
| chrX_79962926_79975155_- | BRWD3 | hsa_circ_0001936 | -6.218918078 | 0.003315516 | 0.06324503 | 0 | 0.26396902 | 0.467706348 | 0.496547784 | 0.15012586 | 0.538154488 | 0.090307355 | 0.196571937 |
| chr8_116599228_116635985_- | TRPS1 | hsa_circ_0085362 | -6.237969399 | 7.64E-06 | 0.001286697 | 4.088071422 | 5.882738165 | 0 | 4.897766774 | 3.603020644 | 6.300892129 | 2.79952799 | 5.258299325 |
| chr2_162036125_162061304_+ | TANK | hsa_circ_0005227 | -6.28874052 | 0.011301973 | 0.120913176 | 0 | 0.30167888 | 0 | 0.157992477 | 0.15012586 | 0.42603897 | 0.270922064 | 0.245714922 |
| chr4_128590191_128609022_+ | INTU | hsa_circ_0003390 | -6.294184562 | 0.010487541 | 0.115415926 | 0.42290394 | 0.3770986 | 0 | 0.767392029 | 0 | 0.67269311 | 0.451536773 | 0.466858351 |
| chr3_32483332_32496034_+ | CMTM7 | hsa_circ_0007783 | -6.31411838 | 0.031278647 | 0.220367329 | 0 | 0.11312958 | 0 | 0.112851769 | 0 | 0.112115518 | 0.180614709 | 0.196571937 |
| chr2_160980302_160983112_- | ITGB6 | hsa_circ_0056856 | -6.334103971 | 0.03564919 | 0.237114023 | 0 | 0.11312958 | 0 | 0.519118137 | 0 | 0.24665414 | 0.722458836 | 0.270286414 |
| chr3_123649949_123667979_- | CCDC14 | NA | -6.353929599 | 0.01759637 | 0.158057516 | 0 | 0.30167888 | 0.233853174 | 0.135422123 | 0.15012586 | 0.24665414 | 0 | 0.147428953 |
| chr11_18312989_18313566_- | HPS5 | hsa_circ_0000279 | -6.369645557 | 0.023058908 | 0.181420668 | 0.14096798 | 0.11312958 | 0.467706348 | 0.18056283 | 0 | 0.515731384 | 0 | 0.516001336 |
| chr2_190584298_190585513_+ | ANKAR | NA | -6.371953443 | 0.047471462 | 0.275825565 | 0 | 0.07541972 | 0 | 0.225703538 | 0 | 0.112115518 | 0.090307355 | 0.049142984 |
| chr14_64465632_64489581_+ | SYNE2 | NA | -6.403828004 | 0.007506723 | 0.099416403 | 0 | 0.980456361 | 0.233853174 | 0.022570354 | 0.45037758 | 0.695116214 | 0.090307355 | 0.442286859 |
| chr2_72945232_72960247_- | EXOC6B | hsa_circ_0009043 | -6.408801306 | 0.001604745 | 0.041073343 | 0.7048399 | 2.488850762 | 3.040091265 | 2.053902195 | 0.750629301 | 1.8386945 | 0.090307355 | 1.277717593 |
| chr16_53907698_53968021_+ | FTO | hsa_circ_0039400 | -6.420816337 | 0.020751746 | 0.171663202 | 0 | 0.07541972 | 0 | 0.18056283 | 0.15012586 | 0.403615866 | 0.090307355 | 0.098285969 |
| chr11_77336008_77340944_- | CLNS1A | hsa_circ_0004593 | -6.432684371 | 0.019775743 | 0.168719628 | 0.14096798 | 0.45251832 | 0 | 0.112851769 | 0.15012586 | 0.089692415 | 0 | 0.22114343 |
| chr19_24014399_24016323_+ | not_annotated | NA | -6.443482244 | 0.007955068 | 0.101804733 | 0.14096798 | 0.52793804 | 0 | 0.135422123 | 0.45037758 | 0.403615866 | 0 | 0.22114343 |
| chrX_44935942_44950109_+ | KDM6A | NA | -6.445777192 | 0.042559994 | 0.259046425 | 0 | 0.15083944 | 0 | 0.135422123 | 0.15012586 | 0.112115518 | 0 | 0.049142984 |
| chr18_76856476_76936907_+ | ATP9B | hsa_circ_0048000 | -6.480752289 | 0.028734287 | 0.209749921 | 0 | 0.07541972 | 0 | 0.496547784 | 0 | 0.381192762 | 0.812766191 | 0.319429398 |
| chr4_152403676_152510078_+ | FAM160A1 | NA | -6.53155943 | 0.04406009 | 0.262800893 | 0 | 0.15083944 | 0 | 0.090281415 | 0.15012586 | 0.089692415 | 0 | 0.098285969 |
| chr4_128842679_128861152_- | MFSD8 | hsa_circ_0009133 | -6.54192534 | 0.00293231 | 0.057698654 | 0.56387192 | 1.432974681 | 0 | 0.270844246 | 0.30025172 | 1.076308976 | 0.361229418 | 0.614287304 |
| chr13_28830429_28835595_+ | PAN3 | hsa_circ_0008902 | -6.567473024 | 0.004833365 | 0.079455799 | 0.28193596 | 0.26396902 | 0 | 0.699680968 | 0.30025172 | 0.313923451 | 0 | 0.34400089 |
| chr18_29691717_29704808_+ | RNF138 | hsa_circ_0047378 | -6.585595644 | 0.019708057 | 0.168719628 | 0 | 0.22625916 | 0.233853174 | 0.090281415 | 0 | 0.156961726 | 0.090307355 | 0.319429398 |
| chr14_56078737_56086030_+ | KTN1 | hsa_circ_0032029 | -6.616696248 | 0.042560018 | 0.259046425 | 0 | 0.5656479 | 0 | 0 | 0.900755161 | 0.313923451 | 0 | 0.319429398 |
| chr5_40852276_40854198_+ | CARD6 | hsa_circ_0005895 | -6.647436221 | 0.007778218 | 0.101468111 | 0.14096798 | 0.26396902 | 0 | 0.406266368 | 0.45037758 | 0.336346555 | 0 | 0.34400089 |
| chr8_135824853_135825412_- | not_annotated | NA | -6.676210007 | 0.003462363 | 0.06482313 | 0 | 0.30167888 | 0 | 0.586829199 | 0.15012586 | 0.358769659 | 0.451536773 | 0.393143875 |
| chr14_75513079_75516421_- | MLH3 | hsa_circ_0032649 | -6.701531413 | 0.00213914 | 0.049085903 | 0.7048399 | 1.621523981 | 0.233853174 | 0.812532737 | 0.15012586 | 0.896924147 | 0.361229418 | 0.663430289 |
| chr3_71542577_71551139_- | FOXP1 | NA | -6.734274856 | 0.039787763 | 0.248305114 | 0 | 0.26396902 | 0 | 0.112851769 | 0 | 0.24665414 | 0.180614709 | 0.073714477 |
| chr6_110036281_110064975_+ | FIG4 | hsa_circ_0077607 | -6.74896017 | 0.046679453 | 0.273582187 | 0 | 0.07541972 | 0 | 0.067711061 | 0 | 0.067269311 | 0.090307355 | 0.147428953 |
| chr4_156617908_156643344_+ | GUCY1A3 | NA | -6.775052856 | 0.032845705 | 0.226668994 | 0 | 0.03770986 | 0 | 0.248273892 | 0.45037758 | 0.269077244 | 0 | 0.22114343 |
| chr3_47702784_47719801_- | SMARCC1 | hsa_circ_0003602 | -6.80829241 | 0.043362255 | 0.260538177 | 0 | 0.22625916 | 0 | 0.045140708 | 0 | 0.112115518 | 0.090307355 | 0.073714477 |
| chr7_22999875_23030758_- | FAM126A | hsa_circ_0008951 | -6.887001622 | 0.014370633 | 0.139699131 | 0.28193596 | 2.715109922 | 0.467706348 | 0.18056283 | 0.600503441 | 0.583000695 | 0 | 0.860002226 |
| chr6_22020568_22111149_+ | CASC15 | hsa_circ_0075830 | -6.932536152 | 0.03747024 | 0.242727879 | 0 | 0.15083944 | 0 | 0.157992477 | 0 | 0.044846207 | 0.090307355 | 0.098285969 |
| chr12_29491464_29494749_- | ERGIC2 | NA | -6.950340367 | 0.037424135 | 0.242727879 | 0.14096798 | 0.07541972 | 0 | 0.112851769 | 0 | 0.134538622 | 0 | 0.147428953 |
| chr10_37507908_37508825_+ | ANKRD30A | NA | -6.957165882 | 0.03698684 | 0.242030392 | 0 | 0.5656479 | 0 | 0 | 0.45037758 | 0.291500348 | 0 | 0.122857461 |
| chr15_61194521_61195883_- | not_annotated | NA | -7.02040415 | 0.037693647 | 0.242727879 | 0 | 0.15083944 | 0 | 0.067711061 | 0 | 0.156961726 | 0.090307355 | 0.073714477 |
| chr18_8113484_8143777_+ | PTPRM | hsa_circ_0002872 | -7.037520713 | 0.00608575 | 0.090480784 | 0.28193596 | 0.678777481 | 0.233853174 | 0.65454026 | 0 | 0.067269311 | 0 | 0.098285969 |
| chr2_165548731_165552346_- | COBLL1 | NA | -7.061177241 | 0.004714495 | 0.079439239 | 0 | 0.41480846 | 0 | 0.428836722 | 0.45037758 | 0.336346555 | 0.090307355 | 0.245714922 |
| chr7_23015829_23030758_- | FAM126A | hsa_circ_0005251 | -7.078046413 | 0.007102669 | 0.097697937 | 0 | 0.45251832 | 0.233853174 | 0.248273892 | 0 | 0.358769659 | 0.180614709 | 0.196571937 |
| chr1_35879573_35881315_+ | ZMYM4 | hsa_circ_0004709 | -7.091950846 | 0.036565887 | 0.240834606 | 0.42290394 | 0.22625916 | 0 | 0.157992477 | 0 | 0.224231037 | 0 | 0.196571937 |
| chr3_71733723_71759635_- | EIF4E3 | hsa_circ_0001322 | -7.236456555 | 0.031555528 | 0.220960902 | 0.14096798 | 0.11312958 | 0 | 0.383696015 | 0 | 0.089692415 | 0 | 0.049142984 |
| chr22_41521868_41536261_+ | EP300 | NA | -7.275651992 | 0.029053052 | 0.211313923 | 0 | 0.3770986 | 0 | 0.157992477 | 0 | 0.291500348 | 0.451536773 | 0.196571937 |
| chr7_158672375_158711560_+ | WDR60 | hsa_circ_0083229 | -7.292940079 | 0.030695444 | 0.218542914 | 0 | 0.15083944 | 0 | 0.090281415 | 0.15012586 | 0.089692415 | 0 | 0.147428953 |
| chr9_80409379_80430686_- | GNAQ | hsa_circ_0087264 | -7.296277673 | 0.025595884 | 0.192397315 | 0 | 0.52793804 | 0 | 0.090281415 | 0.30025172 | 0.269077244 | 0 | 0.024571492 |
| chr1_8568686_8617582_- | RERE | hsa_circ_0005829 | -7.309633946 | 0.025773407 | 0.193014184 | 0 | 0.22625916 | 0.233853174 | 0.135422123 | 0 | 0.089692415 | 0 | 0.098285969 |
| chr8_116564140_116635985_- | TRPS1 | NA | -7.326608847 | 0.036907466 | 0.242030392 | 0 | 0.1885493 | 0 | 0.022570354 | 0.30025172 | 0.156961726 | 0 | 0.34400089 |
| chr4_148860976_148887990_+ | ARHGAP10 | hsa_circ_0007265 | -7.450585315 | 0.027076891 | 0.199088992 | 0 | 0.15083944 | 0 | 0.112851769 | 0.15012586 | 0.179384829 | 0 | 0.049142984 |
| chr1_155640111_155649303_- | YY1AP1 | NA | -7.496440608 | 0.025444389 | 0.192397315 | 0 | 0.15083944 | 0 | 0.157992477 | 0.15012586 | 0.156961726 | 0 | 0.049142984 |
| chr2_197777606_197787849_- | PGAP1 | hsa_circ_0004191 | -7.502974755 | 0.028342504 | 0.207639651 | 0 | 0.11312958 | 0 | 0.067711061 | 0.15012586 | 0.201807933 | 0 | 0.073714477 |
| chr20_34302107_34313077_- | RBM39 | hsa_circ_0004870 | -7.514137093 | 0.005537517 | 0.085472202 | 0.98677586 | 1.206715521 | 0.233853174 | 0.315984953 | 0.15012586 | 0.739962421 | 0 | 0.737144765 |
| chr15_69502655_69553668_+ | GLCE | NA | -7.567964653 | 0.022421092 | 0.178496048 | 0 | 0.15083944 | 0.233853174 | 0.135422123 | 0 | 0.156961726 | 0 | 0.098285969 |
| chr6_76412361_76421132_+ | SENP6 | hsa_circ_0077096 | -7.623541302 | 0.00142377 | 0.038903546 | 0.28193596 | 0.754197201 | 0 | 0.248273892 | 0.30025172 | 0.493308281 | 0 | 0.22114343 |
| chr1_8555123_8617582_- | RERE | hsa_circ_0006837 | -7.712450687 | 0.013697674 | 0.134911324 | 0 | 0.11312958 | 0 | 0.293414599 | 0 | 0.358769659 | 0.451536773 | 0.34400089 |
| chr1_67356837_67371058_- | WDR78 | hsa_circ_0006677 | -7.723708253 | 0.00913356 | 0.109278452 | 0.98677586 | 1.508394401 | 0 | 0.406266368 | 1.050881021 | 1.053885872 | 0 | 0.958288195 |
| chr4_56269403_56284152_+ | TMEM165 | NA | -7.738505514 | 0.023599655 | 0.183757177 | 0 | 0.1885493 | 0 | 0.090281415 | 0.15012586 | 0.112115518 | 0 | 0.122857461 |
| chr13_33109906_33111164_- | N4BP2L2 | NA | -7.75693593 | 0.020147406 | 0.169037575 | 0.28193596 | 0.49022818 | 0 | 0.022570354 | 0 | 0.24665414 | 0 | 0.098285969 |
| chr10_126631026_126650977_+ | ZRANB1 | hsa_circ_0004451 | -7.790102743 | 0.022709071 | 0.179366179 | 0.14096798 | 0.30167888 | 0 | 0.090281415 | 0 | 0.089692415 | 0 | 0.073714477 |
| chr2_36623757_36691798_+ | CRIM1 | hsa_circ_0005579 | -7.832318292 | 0.021460218 | 0.174970006 | 0 | 0.22625916 | 0 | 0.225703538 | 0 | 0.067269311 | 0.090307355 | 0.073714477 |
| chr14_61262935_61285565_+ | MNAT1 | hsa_circ_0032116 | -7.862033053 | 0.021031437 | 0.172259554 | 0.14096798 | 0.22625916 | 0 | 0.248273892 | 0 | 0.089692415 | 0 | 0.049142984 |
| chr19_24014399_24015914_+ | not_annotated | NA | -7.868526188 | 0.045578098 | 0.267910179 | 0 | 0.26396902 | 0 | 0 | 0.30025172 | 0.224231037 | 0 | 0.22114343 |
| chr3_44970798_44975476_- | ZDHHC3 | hsa_circ_0002962 | -7.900441916 | 0.023335669 | 0.18288652 | 0 | 0.3770986 | 0 | 0.112851769 | 0 | 0.067269311 | 0.090307355 | 0.073714477 |
| chr16_53907698_53922863_+ | FTO | hsa_circ_0005941 | -7.970042067 | 0.000251838 | 0.015913006 | 0.14096798 | 2.375721182 | 0.701559523 | 1.850769011 | 0.900755161 | 1.367809323 | 0.541844127 | 1.376003562 |
| chr5_145176005_145205763_- | PRELID2 | hsa_circ_0008647 | -8.019214635 | 0.016491621 | 0.150887139 | 0 | 0.03770986 | 0 | 0.361125661 | 0 | 0.24665414 | 0.270922064 | 0.270286414 |
| chr14_88883055_88904247_+ | SPATA7 | NA | -8.033605771 | 0.037687024 | 0.242727879 | 0 | 0.754197201 | 0 | 0.406266368 | 0.30025172 | 0.044846207 | 0 | 0.073714477 |
| chr1_63042933_63052295_- | DOCK7 | hsa_circ_0002032 | -8.045540535 | 0.018444057 | 0.160749493 | 0.14096798 | 0.15083944 | 0 | 0.157992477 | 0 | 0.156961726 | 0 | 0.098285969 |
| chr17_60106902_60112969_- | MED13 | hsa_circ_0045096 | -8.096686049 | 0.022120158 | 0.17793583 | 0 | 0.11312958 | 0 | 0.067711061 | 0.15012586 | 0.156961726 | 0 | 0.147428953 |
| chr16_53188359_53191453_+ | CHD9 | hsa_circ_0000701 | -8.284075041 | 0.034558503 | 0.232150474 | 0 | 0.22625916 | 0.467706348 | 0.090281415 | 0 | 0.291500348 | 0 | 0.245714922 |
| chr7_77200395_77230123_+ | PTPN12 | hsa_circ_0080835 | -8.355293834 | 0.020719776 | 0.171663202 | 0 | 0.52793804 | 0 | 0 | 0.30025172 | 0.403615866 | 0 | 0.049142984 |
| chr10_112723883_112745523_+ | SHOC2 | hsa_circ_0020028 | -8.374536228 | 0.005377707 | 0.084950961 | 0.14096798 | 0.791907061 | 0 | 0.022570354 | 0.30025172 | 0.269077244 | 0 | 0.442286859 |
| chr13_28748409_28771483_+ | PAN3 | hsa_circ_0008760 | -8.431882497 | 0.014925085 | 0.143026169 | 0 | 0.33938874 | 0 | 0.203133184 | 0 | 0.201807933 | 0.180614709 | 0.122857461 |
| chr11_103173822_103229087_+ | DYNC2H1 | hsa_circ_0024144 | -8.493188826 | 0.017782899 | 0.158057516 | 0 | 0.33938874 | 0.233853174 | 0.112851769 | 0 | 0.179384829 | 0 | 0.073714477 |
| chr7_80418622_80435074_- | SEMA3C | hsa_circ_0002714 | -8.650156512 | 0.001486977 | 0.040088901 | 0.84580788 | 1.395264821 | 0.233853174 | 0.677110614 | 0.15012586 | 0.874501043 | 0.090307355 | 0.860002226 |
| chr5_95091100_95099324_+ | RHOBTB3 | hsa_circ_0007444 | -8.761867464 | 0.000381593 | 0.01841364 | 0.7048399 | 1.621523981 | 1.403119045 | 1.78305795 | 0.45037758 | 2.332002781 | 0.090307355 | 1.670861468 |
| chr9_88574708_88611492_+ | NAA35 | NA | -8.775043263 | 0.016407067 | 0.15079586 | 0 | 0.15083944 | 0 | 0.067711061 | 0.15012586 | 0.134538622 | 0 | 0.196571937 |
| chr4_152095822_152108615_- | SH3D19 | hsa_circ_0071261 | -8.786631414 | 0.015382926 | 0.146029463 | 0 | 0.11312958 | 0 | 0.18056283 | 0 | 0.179384829 | 0.090307355 | 0.098285969 |
| chr6_145103058_145124283_+ | UTRN | hsa_circ_0001648 | -8.916609281 | 0.001544926 | 0.04033967 | 0.42290394 | 0.603357761 | 0 | 0.609399552 | 0.15012586 | 1.09873208 | 0.270922064 | 0.835430734 |
| chr2_120684174_120702816_+ | PTPN4 | hsa_circ_0056248 | -8.949009001 | 0.014653123 | 0.141088645 | 0 | 0.22625916 | 0 | 0.293414599 | 0.15012586 | 0.089692415 | 0 | 0.098285969 |
| chr11_18312989_18314523_- | HPS5 | hsa_circ_0000280 | -8.976390502 | 0.00076468 | 0.027610405 | 0.56387192 | 1.885493002 | 0.935412697 | 1.512213704 | 0.750629301 | 1.524771049 | 0 | 1.47428953 |
| chr5_83356119_83362425_- | EDIL3 | hsa_circ_0073244 | -9.033338095 | 0.015681222 | 0.147476426 | 0.14096798 | 0.15083944 | 0 | 0.090281415 | 0 | 0.134538622 | 0 | 0.22114343 |
| chr3_183361268_183382827_+ | KLHL24 | NA | -9.109321327 | 0.009579254 | 0.111385233 | 0 | 0.22625916 | 0.467706348 | 0.315984953 | 0 | 0.24665414 | 0 | 0.172000445 |
| chr13_30801549_30857928_- | KATNAL1 | hsa_circ_0008068 | -9.161891785 | 0.013626123 | 0.134911324 | 0 | 0.3770986 | 0.233853174 | 0.135422123 | 0 | 0.179384829 | 0 | 0.073714477 |
| chr19_22157531_22171711_- | ZNF208 | NA | -9.166852047 | 0.01051821 | 0.115415926 | 0 | 0.15083944 | 0 | 0.541688491 | 0.45037758 | 0.201807933 | 0 | 0.319429398 |
| chr11_118656761_118657227_- | DDX6 | hsa_circ_0004293 | -9.214793399 | 0.001348808 | 0.037879032 | 0.28193596 | 0.45251832 | 0 | 0.338555307 | 0 | 0.358769659 | 0.090307355 | 0.368572383 |
| chr16_47531310_47630442_+ | PHKB | hsa_circ_0039264 | -9.230680293 | 0.01203117 | 0.125397042 | 0 | 0.15083944 | 0.233853174 | 0.225703538 | 0 | 0.179384829 | 0 | 0.098285969 |
| chr11_34093273_34098189_+ | CAPRIN1 | hsa_circ_0005974 | -9.368270162 | 0.002361366 | 0.051690451 | 0.14096798 | 0.33938874 | 0.233853174 | 0.383696015 | 0 | 0.291500348 | 0 | 0.22114343 |
| chr10_34648075_34673182_- | PARD3 | hsa_circ_0005486 | -9.409058932 | 0.01525785 | 0.145525341 | 0 | 0.07541972 | 0 | 0.090281415 | 0 | 0.112115518 | 0.090307355 | 0.22114343 |
| chr10_34620045_34649187_- | PARD3 | hsa_circ_0002487 | -9.63744138 | 0.011996618 | 0.125397042 | 0 | 0.11312958 | 0 | 0.225703538 | 0 | 0.112115518 | 0.090307355 | 0.147428953 |
| chr1_78183552_78191447_- | USP33 | hsa_circ_0000087 | -9.827208041 | 0.003674071 | 0.0681557 | 0.56387192 | 0.678777481 | 0 | 0.248273892 | 0 | 0.403615866 | 0 | 0.22114343 |
| chr5_79745410_79770649_+ | ZFYVE16 | NA | -9.871925536 | 0.012902171 | 0.131096434 | 0 | 0.5656479 | 0 | 0.203133184 | 0 | 0.112115518 | 0.090307355 | 0.049142984 |
| chr1_45553553_45553909_- | ZSWIM5 | NA | -9.876420765 | 0.009640166 | 0.111385233 | 0 | 0.15083944 | 0.233853174 | 0.315984953 | 0 | 0.179384829 | 0 | 0.073714477 |
| chr16_53288350_53308214_+ | CHD9 | hsa_circ_0000702 | -9.906039743 | 0.008903346 | 0.107711677 | 0.28193596 | 0.30167888 | 0 | 0.157992477 | 0 | 0.358769659 | 0 | 0.196571937 |
| chr17_49294695_49302570_- | MBTD1 | hsa_circ_0003871 | -9.968997441 | 0.010559815 | 0.115415926 | 0 | 0.15083944 | 0 | 0.18056283 | 0 | 0.067269311 | 0.090307355 | 0.196571937 |
| chr8_116599228_116617229_- | TRPS1 | hsa_circ_0085360 | -10.05548236 | 0.010731672 | 0.116039786 | 0 | 0.07541972 | 0 | 0.18056283 | 0.15012586 | 0.269077244 | 0 | 0.098285969 |
| chr15_72338065_72338975_- | MYO9A | hsa_circ_0006509 | -10.07822744 | 0.002770908 | 0.055473024 | 0.14096798 | 0.942746501 | 0.467706348 | 0.135422123 | 0 | 0.560577592 | 0 | 0.466858351 |
| chr10_103552596_103570071_- | MGEA5 | hsa_circ_0019607 | -10.12142233 | 0.000321818 | 0.018290686 | 0.14096798 | 0.678777481 | 0.233853174 | 0.270844246 | 0 | 0.24665414 | 0.090307355 | 0.688001781 |
| chr5_145197457_145205763_- | PRELID2 | hsa_circ_0006528 | -10.19584362 | 0.000613664 | 0.024052684 | 0 | 0.942746501 | 0 | 0.519118137 | 0.15012586 | 0.24665414 | 0.270922064 | 0.491429843 |
| chr12_32751431_32764217_+ | FGD4 | hsa_circ_0025843 | -10.25883689 | 0.006881598 | 0.095493555 | 0.28193596 | 0.49022818 | 0 | 0.067711061 | 0 | 0.313923451 | 0 | 0.196571937 |
| chr11_77336008_77336863_- | CLNS1A | hsa_circ_0000343 | -10.27663746 | 0.006485085 | 0.092998873 | 0 | 0.41480846 | 0 | 0.112851769 | 0 | 0.201807933 | 0.180614709 | 0.245714922 |
| chr9_710804_713464_+ | KANK1 | hsa_circ_0005062 | -10.30633363 | 0.009300574 | 0.110622125 | 0 | 0.15083944 | 0 | 0.338555307 | 0 | 0.156961726 | 0.090307355 | 0.098285969 |
| chr2_169018297_169038600_- | STK39 | hsa_circ_0001079 | -10.33188529 | 0.00837147 | 0.104488346 | 0.14096798 | 0.22625916 | 0 | 0.248273892 | 0 | 0.179384829 | 0 | 0.098285969 |
| chr7_129679304_129688984_- | ZC3HC1 | hsa_circ_0004364 | -10.35033892 | 0.000395809 | 0.01841364 | 0 | 0.41480846 | 0.233853174 | 0.428836722 | 0 | 0.313923451 | 0.180614709 | 0.491429843 |
| chr12_83250789_83251359_+ | TMTC2 | hsa_circ_0002886 | -10.47741937 | 0.001784215 | 0.044539295 | 0 | 0.22625916 | 0 | 0.293414599 | 0.15012586 | 0.269077244 | 0.090307355 | 0.368572383 |
| chr3_174241203_174271547_+ | not_annotated | NA | -10.53455296 | 0.006610257 | 0.094069891 | 0 | 0.980456361 | 0 | 0.067711061 | 0.30025172 | 0.291500348 | 0 | 0.098285969 |
| chr1_215342542_215345526_+ | KCNK2 | hsa_circ_0008788 | -10.715815 | 0.00978764 | 0.112157328 | 0.14096798 | 0.15083944 | 0 | 0.112851769 | 0 | 0.403615866 | 0 | 0.122857461 |
| chr10_128768966_128908618_+ | DOCK1 | hsa_circ_0020396 | -10.87478028 | 0.009576935 | 0.111385233 | 0 | 0.07541972 | 0 | 0.090281415 | 0 | 0.134538622 | 0.090307355 | 0.270286414 |
| chr14_32559708_32586493_+ | ARHGAP5 | hsa_circ_0031584 | -10.88865384 | 0.003744264 | 0.068206322 | 0 | 0.30167888 | 0.467706348 | 0.338555307 | 0 | 0.470885177 | 0 | 0.147428953 |
| chr5_108281831_108295048_+ | FER | hsa_circ_0003032 | -11.02323752 | 0.006895183 | 0.095493555 | 0 | 0.33938874 | 0.233853174 | 0.135422123 | 0 | 0.201807933 | 0 | 0.172000445 |
| chr14_21825356_21829372_- | SUPT16H | hsa_circ_0000522 | -11.14917746 | 0.006187325 | 0.090695796 | 0 | 0.22625916 | 0.233853174 | 0.270844246 | 0 | 0.269077244 | 0 | 0.073714477 |
| chr15_56680670_56687032_+ | TEX9 | hsa_circ_0000603 | -11.15876862 | 0.003460566 | 0.06482313 | 0 | 0.33938874 | 0 | 0.112851769 | 0.45037758 | 0.42603897 | 0 | 0.466858351 |
| chr2_24357989_24369956_+ | FAM228B | hsa_circ_0000982 | -11.25579915 | 0.005648418 | 0.085872936 | 0.28193596 | 1.432974681 | 0 | 0.022570354 | 0.900755161 | 0.964193458 | 0 | 1.621718483 |
| chr7_140494108_140508795_- | BRAF | hsa_circ_0006961 | -11.66605034 | 0.005258217 | 0.084381855 | 0 | 0.22625916 | 0.233853174 | 0.203133184 | 0 | 0.269077244 | 0 | 0.147428953 |
| chr10_86177527_86198463_+ | CCSER2 | hsa_circ_0003018 | -12.21704733 | 0.005911807 | 0.088545739 | 0 | 0.30167888 | 0 | 0.067711061 | 0 | 0.112115518 | 0.090307355 | 0.245714922 |
| chr2_148653870_148657467_+ | ACVR2A | hsa_circ_0001073 | -12.37577769 | 0.000997583 | 0.033067432 | 0.56387192 | 1.847783142 | 0 | 0.880243798 | 0.900755161 | 1.233270701 | 0 | 0.933716702 |
| chr13_20638591_20657172_+ | ZMYM2 | hsa_circ_0029638 | -12.58947838 | 0.00478003 | 0.079455799 | 0.14096798 | 0.3770986 | 0 | 0.067711061 | 0 | 0.269077244 | 0 | 0.147428953 |
| chr3_57276884_57282379_+ | APPL1 | hsa_circ_0001317 | -12.61656254 | 0.005464412 | 0.084992626 | 0 | 0.45251832 | 0 | 0.045140708 | 0.15012586 | 0.179384829 | 0 | 0.22114343 |
| chr10_126097111_126100769_- | OAT | hsa_circ_0008898 | -12.62075873 | 0.004248954 | 0.07343064 | 0 | 0.26396902 | 0 | 0.135422123 | 0 | 0.224231037 | 0.090307355 | 0.172000445 |
| chr9_14146688_14179779_- | NFIB | hsa_circ_0086376 | -12.6211732 | 1.32E-05 | 0.001902498 | 0 | 2.488850762 | 0.935412697 | 2.482738918 | 0.750629301 | 1.905963811 | 0.361229418 | 1.720004452 |
| chr1_97981282_98015300_- | DPYD | hsa_circ_0006323 | -12.73753835 | 0.004129971 | 0.071989666 | 0 | 0.33938874 | 0 | 0.18056283 | 0 | 0.112115518 | 0.090307355 | 0.196571937 |
| chr6_76331248_76388643_+ | SENP6 | NA | -12.94600125 | 0.033864284 | 0.22977712 | 0 | 0.07541972 | 0 | 0.045140708 | 0 | 0.044846207 | 0 | 0.049142984 |
| chr15_57730183_57754090_+ | CGNL1 | hsa_circ_0035432 | -12.97350759 | 0.002384902 | 0.051690451 | 0.28193596 | 0.33938874 | 0 | 0.248273892 | 0 | 0.269077244 | 0 | 0.516001336 |
| chr10_128768966_129055693_+ | DOCK1 | hsa_circ_0020399 | -13.36620718 | 0.001679415 | 0.04244722 | 0.28193596 | 0.49022818 | 0 | 0.090281415 | 0 | 0.583000695 | 0 | 0.294857906 |
| chr15_94899366_94945248_+ | MCTP2 | hsa_circ_0000660 | -14.71480845 | 0.038278926 | 0.243412153 | 0 | 0.5656479 | 7.015595226 | 0.270844246 | 0 | 0.448462073 | 0 | 0.540572828 |
| chr3_183361268_183369064_+ | KLHL24 | hsa_circ_0001367 | -14.75009633 | 0.002686317 | 0.055425844 | 0.14096798 | 1.508394401 | 0 | 1.105947336 | 0 | 0.852077939 | 0.903073545 | 0.56514432 |
| chr14_89178615_89181539_- | EML5 | hsa_circ_0032873 | -14.96741781 | 0.024730282 | 0.190131673 | 0 | 0.07541972 | 0 | 0.045140708 | 0 | 0.044846207 | 0 | 0.073714477 |
| chr18_51807037_51813781_+ | POLI | NA | -15.33074225 | 0.021982077 | 0.17779104 | 0 | 0.11312958 | 0 | 0.067711061 | 0 | 0.044846207 | 0 | 0.049142984 |
| chr2_72692289_72802797_- | EXOC6B | NA | -15.63656437 | 0.021042586 | 0.172259554 | 0 | 0.07541972 | 0 | 0.112851769 | 0 | 0.044846207 | 0 | 0.049142984 |
| chr1_100889778_100908552_+ | CDC14A | hsa_circ_0000097 | -16.78296733 | 0.017522602 | 0.158057516 | 0 | 0.11312958 | 0 | 0.067711061 | 0 | 0.067269311 | 0 | 0.049142984 |
| chr1_202418117_202471133_+ | PPP1R12B | NA | -16.85060046 | 0.018007736 | 0.159002802 | 0 | 0.07541972 | 0 | 0.090281415 | 0 | 0.044846207 | 0 | 0.073714477 |
| chr16_53878067_53922863_+ | FTO | hsa_circ_0039398 | -17.04201942 | 0.000754444 | 0.027610405 | 0 | 0.603357761 | 0 | 0.361125661 | 0.15012586 | 0.291500348 | 0 | 0.098285969 |
| chr1_6880241_6948959_+ | CAMTA1 | hsa_circ_0002975 | -17.11452953 | 0.016372551 | 0.15079586 | 0 | 0.11312958 | 0 | 0.112851769 | 0 | 0.044846207 | 0 | 0.049142984 |
| chr13_23927924_23945304_- | SACS | hsa_circ_0003408 | -17.40523392 | 0.016611713 | 0.151301274 | 0 | 0.07541972 | 0 | 0.067711061 | 0 | 0.067269311 | 0 | 0.073714477 |
| chr15_57730183_57734676_+ | CGNL1 | hsa_circ_0035431 | -17.80250011 | 0.000150155 | 0.010250401 | 0 | 0.45251832 | 0 | 0.65454026 | 0 | 0.605423799 | 0.270922064 | 0.516001336 |
| chr21_40665737_40670508_- | BRWD1 | hsa_circ_0061757 | -18.47737451 | 0.013744675 | 0.134911324 | 0 | 0.07541972 | 0 | 0.112851769 | 0 | 0.089692415 | 0 | 0.049142984 |
| chr3_30686239_30715738_+ | TGFBR2 | hsa_circ_0064654 | -18.52442027 | 0.014176432 | 0.138477027 | 0 | 0.11312958 | 0 | 0.045140708 | 0 | 0.112115518 | 0 | 0.049142984 |
| chr19_53077330_53087450_+ | ZNF701 | NA | -18.76555876 | 0.016045755 | 0.149513903 | 0 | 0.07541972 | 0 | 0.045140708 | 0 | 0.044846207 | 0 | 0.122857461 |
| chr9_130206308_130207528_+ | ZNF79 | hsa_circ_0006984 | -19.43833333 | 0.012629661 | 0.128975629 | 0 | 0.11312958 | 0 | 0.067711061 | 0 | 0.044846207 | 0 | 0.098285969 |
| chr2_132310586_132339702_+ | not_annotated | NA | -19.58680536 | 0.011203237 | 0.120494394 | 0 | 0.07541972 | 0 | 0.18056283 | 0 | 0.067269311 | 0 | 0.049142984 |
| chr1_42730786_42789498_- | FOXJ3 | NA | -20.36164307 | 0.01247766 | 0.12807019 | 0 | 0.11312958 | 0 | 0.045140708 | 0 | 0.044846207 | 0 | 0.122857461 |
| chr1_78097535_78107340_- | ZZZ3 | NA | -20.64883201 | 0.000359284 | 0.018290686 | 0 | 0.980456361 | 0.467706348 | 0.293414599 | 0 | 0.583000695 | 0 | 0.688001781 |
| chr3_175181189_175189546_+ | NAALADL2 | NA | -20.73254684 | 0.010717103 | 0.116039786 | 0 | 0.11312958 | 0 | 0.045140708 | 0 | 0.112115518 | 0 | 0.073714477 |
| chr17_60087911_60140662_- | MED13 | NA | -21.28451403 | 0.009892542 | 0.112157328 | 0 | 0.15083944 | 0 | 0.045140708 | 0 | 0.134538622 | 0 | 0.049142984 |
| chr20_46252655_46262380_+ | NCOA3 | hsa_circ_0001165 | -21.31848814 | 0.008707294 | 0.106703924 | 0 | 0.15083944 | 0 | 0.090281415 | 0 | 0.067269311 | 0 | 0.073714477 |
| chr1_65068489_65107652_+ | CACHD1 | NA | -21.48359119 | 0.009638642 | 0.111385233 | 0 | 0.07541972 | 0 | 0.067711061 | 0 | 0.134538622 | 0 | 0.073714477 |
| chr3_183368084_183369064_+ | KLHL24 | hsa_circ_0001369 | -21.64146471 | 5.14E-05 | 0.004519474 | 0 | 0.867326781 | 0 | 0.586829199 | 0 | 0.448462073 | 0.180614709 | 0.442286859 |
| chr2_110919180_110920712_- | NPHP1 | hsa_circ_0056019 | -21.90375034 | 0.000199653 | 0.013022559 | 0 | 0.5656479 | 0 | 0.225703538 | 0 | 0.291500348 | 0.090307355 | 0.34400089 |
| chr12_96076483_96077487_- | NTN4 | hsa_circ_0006419 | -21.93009473 | 0.009928863 | 0.112157328 | 0 | 0.11312958 | 0 | 0.045140708 | 0 | 0.067269311 | 0 | 0.122857461 |
| chr19_11526613_11527733_- | RGL3 | hsa_circ_0006941 | -22.03047604 | 0.007477553 | 0.099416403 | 0 | 0.07541972 | 0 | 0.135422123 | 0 | 0.134538622 | 0 | 0.049142984 |
| chr19_11833769_11836142_- | ZNF823 | NA | -22.06314992 | 0.008949338 | 0.107711677 | 0 | 0.11312958 | 0 | 0.045140708 | 0 | 0.134538622 | 0 | 0.073714477 |
| chr1_52959283_52975384_- | ZCCHC11 | hsa_circ_0003632 | -22.06526553 | 0.000346911 | 0.018290686 | 0 | 0.26396902 | 0 | 0.067711061 | 0.15012586 | 0.583000695 | 0 | 0.393143875 |
| chr5_80911292_80946158_- | SSBP2 | hsa_circ_0073222 | -22.08226411 | 0.008265237 | 0.103803167 | 0 | 0.11312958 | 0 | 0.067711061 | 0 | 0.156961726 | 0 | 0.049142984 |
| chr17_65941525_65962772_+ | BPTF | hsa_circ_0045471 | -22.1265647 | 0.008850555 | 0.107711677 | 0 | 0.22625916 | 0 | 0.045140708 | 0 | 0.067269311 | 0 | 0.073714477 |
| chr10_111883775_111890244_+ | ADD3 | hsa_circ_0003357 | -22.17330599 | 7.71E-05 | 0.006235848 | 0.14096798 | 0.33938874 | 0 | 0.451407076 | 0 | 0.470885177 | 0 | 0.368572383 |
| chr1_153784207_153785928_- | GATAD2B | NA | -22.3718853 | 0.007509684 | 0.099416403 | 0 | 0.1885493 | 0 | 0.067711061 | 0 | 0.112115518 | 0 | 0.049142984 |
| chr3_185335268_185341870_+ | SENP2 | NA | -23.46712155 | 0.007716004 | 0.101310135 | 0 | 0.15083944 | 0 | 0.045140708 | 0 | 0.067269311 | 0 | 0.122857461 |
| chr2_223765392_223799405_+ | ACSL3 | NA | -23.72786597 | 0.005628951 | 0.085872936 | 0 | 0.15083944 | 0 | 0.135422123 | 0 | 0.112115518 | 0 | 0.049142984 |
| chr2_207728120_207746615_+ | not_annotated | NA | -24.28293508 | 0.006699339 | 0.094069891 | 0 | 0.07541972 | 0 | 0.090281415 | 0 | 0.067269311 | 0 | 0.147428953 |
| chr2_128855003_128867320_+ | UGGT1 | hsa_circ_0056401 | -24.29427277 | 0.006419121 | 0.092841847 | 0 | 0.22625916 | 0 | 0.045140708 | 0 | 0.067269311 | 0 | 0.098285969 |
| chr22_30374431_30387659_+ | MTMR3 | hsa_circ_0002954 | -24.86466814 | 0.004571433 | 0.077675952 | 0 | 0.1885493 | 0 | 0.203133184 | 0 | 0.067269311 | 0 | 0.049142984 |
| chr10_20432224_20453496_+ | PLXDC2 | hsa_circ_0017924 | -24.96477544 | 0.004895171 | 0.079822863 | 0 | 0.11312958 | 0 | 0.112851769 | 0 | 0.134538622 | 0 | 0.073714477 |
| chr20_13539655_13568017_- | TASP1 | hsa_circ_0004580 | -25.1944376 | 0.006669619 | 0.094069891 | 0 | 0.11312958 | 0 | 0.067711061 | 0 | 0.044846207 | 0 | 0.172000445 |
| chr17_58342773_58372162_- | USP32 | hsa_circ_0044949 | -25.74356551 | 0.006428219 | 0.092841847 | 0 | 0.15083944 | 0 | 0.045140708 | 0 | 0.044846207 | 0 | 0.172000445 |
| chr18_76953183_76974038_+ | ATP9B | hsa_circ_0003275 | -26.39522899 | 0.0048027 | 0.079455799 | 0 | 0.22625916 | 0 | 0.045140708 | 0 | 0.067269311 | 0 | 0.122857461 |
| chr14_38256673_38266152_+ | not_annotated | NA | -27.08428913 | 0.003789508 | 0.068414147 | 0 | 0.30167888 | 0 | 0.135422123 | 0 | 0.044846207 | 0 | 0.073714477 |
| chr2_68717322_68805135_+ | APLF | NA | -27.30103942 | 0.003966595 | 0.070354868 | 0 | 0.30167888 | 0 | 0.067711061 | 0 | 0.089692415 | 0 | 0.073714477 |
| chr15_70949269_70957153_- | UACA | hsa_circ_0002250 | -28.01744028 | 0.003724228 | 0.068206322 | 0 | 0.30167888 | 0 | 0.067711061 | 0 | 0.067269311 | 0 | 0.098285969 |
| chr4_144336630_144390430_+ | GAB1 | NA | -28.1060128 | 0.003037922 | 0.059064213 | 0 | 0.1885493 | 0 | 0.270844246 | 0 | 0.044846207 | 0 | 0.073714477 |
| chr3_155547477_155551830_- | SLC33A1 | hsa_circ_0001350 | -29.16075296 | 1.75E-05 | 0.00196193 | 0 | 0.5656479 | 0 | 0.18056283 | 0.15012586 | 0.448462073 | 0 | 0.737144765 |
| chr10_20432224_20466338_+ | PLXDC2 | hsa_circ_0017925 | -29.30285578 | 0.00293915 | 0.057698654 | 0 | 0.1885493 | 0 | 0.135422123 | 0 | 0.044846207 | 0 | 0.147428953 |
| chr5_80785069_80946158_- | SSBP2 | hsa_circ_0073219 | -29.40237912 | 0.003162859 | 0.060907619 | 0 | 0.22625916 | 0 | 0.045140708 | 0 | 0.112115518 | 0 | 0.122857461 |
| chr12_18434938_18446949_+ | PIK3C2G | NA | -30.21813853 | 1.49E-05 | 0.00196193 | 0 | 0.49022818 | 0 | 0.157992477 | 0.15012586 | 0.560577592 | 0 | 0.712573273 |
| chr9_114289548_114296633_+ | ZNF483 | NA | -30.92446367 | 2.84E-05 | 0.002867886 | 0 | 1.621523981 | 0 | 0.744821675 | 0 | 0.201807933 | 0.090307355 | 0.270286414 |
| chr17_37373063_37374426_- | STAC2 | NA | -30.9307899 | 0.002542125 | 0.054107114 | 0 | 0.11312958 | 0 | 0.090281415 | 0 | 0.112115518 | 0 | 0.172000445 |
| chr4_62800558_62863983_+ | LPHN3 | NA | -31.20251553 | 0.002729568 | 0.055473024 | 0 | 0.49022818 | 0 | 0.045140708 | 0 | 0.067269311 | 0 | 0.073714477 |
| chr3_183368084_183390272_+ | KLHL24 | hsa_circ_0006667 | -31.21885476 | 1.71E-05 | 0.00196193 | 0 | 0.905036641 | 0 | 0.270844246 | 0 | 0.515731384 | 0.090307355 | 0.442286859 |
| chrX_32563276_32717410_- | DMD | NA | -31.36839194 | 0.00262291 | 0.054675509 | 0 | 0.11312958 | 0 | 0.090281415 | 0 | 0.089692415 | 0 | 0.196571937 |
| chr9_87317074_87342874_+ | NTRK2 | NA | -31.45766742 | 0.002171159 | 0.049085903 | 0 | 0.11312958 | 0 | 0.112851769 | 0 | 0.24665414 | 0 | 0.073714477 |
| chr12_88898936_88939642_- | KITLG | hsa_circ_0002375 | -31.90305931 | 0.002175495 | 0.049085903 | 0 | 0.11312958 | 0 | 0.112851769 | 0 | 0.112115518 | 0 | 0.172000445 |
| chr10_37430646_37431218_+ | ANKRD30A | NA | -31.98322519 | 0.00220652 | 0.049085903 | 0 | 0.30167888 | 0 | 0.045140708 | 0 | 0.179384829 | 0 | 0.073714477 |
| chr17_37840850_37842272_- | PGAP3 | hsa_circ_0007990 | -32.00212942 | 0.002403018 | 0.051690451 | 0 | 0.11312958 | 0 | 0.18056283 | 0 | 0.044846207 | 0 | 0.196571937 |
| chr4_55561678_55575705_+ | KIT | hsa_circ_0069763 | -32.62095577 | 0.001933616 | 0.046275335 | 0 | 0.07541972 | 0 | 0.18056283 | 0 | 0.24665414 | 0 | 0.073714477 |
| chr3_17549966_17628049_- | TBC1D5 | NA | -33.16478317 | 0.001880258 | 0.04580581 | 0 | 0.11312958 | 0 | 0.090281415 | 0 | 0.179384829 | 0 | 0.147428953 |
| chr1_227327277_227335260_- | CDC42BPA | hsa_circ_0003659 | -33.31983675 | 0.001878282 | 0.04580581 | 0 | 0.3770986 | 0 | 0.045140708 | 0 | 0.156961726 | 0 | 0.073714477 |
| chr7_20406635_20420454_+ | ITGB8 | hsa_circ_0079540 | -33.33102576 | 0.002172945 | 0.049085903 | 0 | 0.15083944 | 0 | 0.090281415 | 0 | 0.067269311 | 0 | 0.22114343 |
| chr8_105080740_105161076_+ | not_annotated | hsa_circ_0005114 | -34.42209627 | 0.002209108 | 0.049085903 | 0 | 0.45251832 | 0 | 0.045140708 | 0 | 0.067269311 | 0 | 0.122857461 |
| chr7_16255691_16317851_- | ISPD | NA | -34.6724444 | 0.001169269 | 0.035852536 | 0 | 0.1885493 | 0 | 0.135422123 | 0 | 0.156961726 | 0 | 0.122857461 |
| chr12_102116946_102124414_+ | CHPT1 | NA | -34.74084825 | 0.00155613 | 0.04033967 | 0 | 0.33938874 | 0 | 0.045140708 | 0 | 0.201807933 | 0 | 0.073714477 |
| chr19_52887102_52888995_+ | ZNF880 | NA | -34.96542908 | 0.001383861 | 0.038331053 | 0 | 0.26396902 | 0 | 0.203133184 | 0 | 0.044846207 | 0 | 0.147428953 |
| chr15_65756099_65772737_- | DPP8 | hsa_circ_0035897 | -35.53987248 | 0.001258446 | 0.036958625 | 0 | 0.1885493 | 0 | 0.090281415 | 0 | 0.134538622 | 0 | 0.172000445 |
| chr10_37507908_37531775_+ | ANKRD30A | NA | -36.05156361 | 0.001160829 | 0.035852536 | 0 | 0.1885493 | 0 | 0.18056283 | 0 | 0.224231037 | 0 | 0.073714477 |
| chr9_87317074_87367000_+ | NTRK2 | hsa_circ_0087374 | -36.19427424 | 0.00125594 | 0.036958625 | 0 | 0.26396902 | 0 | 0.112851769 | 0 | 0.224231037 | 0 | 0.073714477 |
| chr9_14146688_14307519_- | NFIB | NA | -36.21043009 | 0.001100189 | 0.035310821 | 0 | 0.15083944 | 0 | 0.248273892 | 0 | 0.134538622 | 0 | 0.122857461 |
| chr9_5954016_5988545_- | KIAA2026 | NA | -37.11291531 | 0.00155159 | 0.04033967 | 0 | 0.07541972 | 0 | 0.067711061 | 0 | 0.291500348 | 0 | 0.147428953 |
| chr19_9763629_9771550_- | ZNF562 | NA | -38.15218642 | 0.001304326 | 0.03714573 | 0 | 0.15083944 | 0 | 0.067711061 | 0 | 0.291500348 | 0 | 0.122857461 |
| chr5_67522118_67522837_+ | PIK3R1 | hsa_circ_0006411 | -39.03518609 | 0.000851381 | 0.029680892 | 0 | 0.30167888 | 0 | 0.135422123 | 0 | 0.156961726 | 0 | 0.122857461 |
| chr2_43969880_43973102_+ | PLEKHH2 | NA | -41.11944782 | 0.000817901 | 0.029013956 | 0 | 0.30167888 | 0 | 0.067711061 | 0 | 0.201807933 | 0 | 0.147428953 |
| chr5_135483521_135489852_+ | SMAD5 | hsa_circ_0007930 | -43.64044671 | 0.000576284 | 0.02378053 | 0 | 0.15083944 | 0 | 0.203133184 | 0 | 0.179384829 | 0 | 0.196571937 |
| chr13_37614542_37625720_- | SUPT20H | hsa_circ_0000475 | -43.75272265 | 0.000733968 | 0.027483021 | 0 | 0.41480846 | 0 | 0.067711061 | 0 | 0.112115518 | 0 | 0.196571937 |
| chr10_133747962_133761295_+ | PPP2R2D | NA | -43.88033914 | 0.000494236 | 0.021262663 | 0 | 0.11312958 | 0 | 0.406266368 | 0 | 0.224231037 | 0 | 0.098285969 |
| chr8_30332295_30361953_+ | RBPMS | hsa_circ_0006539 | -45.03127976 | 0.000568901 | 0.02378053 | 0 | 0.11312958 | 0 | 0.270844246 | 0 | 0.291500348 | 0 | 0.122857461 |
| chr3_136117591_136221621_- | STAG1 | hsa_circ_0067475 | -45.76233091 | 0.000589321 | 0.023832151 | 0 | 0.15083944 | 0 | 0.18056283 | 0 | 0.134538622 | 0 | 0.270286414 |
| chr1_201821232_201828122_+ | IPO9 | NA | -47.03648841 | 0.000665081 | 0.025373482 | 0 | 0.11312958 | 0 | 0.090281415 | 0 | 0.381192762 | 0 | 0.172000445 |
| chr14_91409422_91467503_- | RPS6KA5 | hsa_circ_0032940 | -47.14794649 | 0.000618566 | 0.024052684 | 0 | 0.11312958 | 0 | 0.225703538 | 0 | 0.448462073 | 0 | 0.073714477 |
| chr15_49917310_49926991_+ | DTWD1 | hsa_circ_0035194 | -47.61711246 | 0.000400692 | 0.01841364 | 0 | 0.41480846 | 0 | 0.135422123 | 0 | 0.156961726 | 0 | 0.172000445 |
| chr10_97141442_97170534_- | SORBS1 | NA | -47.83534137 | 0.000334117 | 0.018290686 | 0 | 0.30167888 | 0 | 0.338555307 | 0 | 0.134538622 | 0 | 0.147428953 |
| chr14_50175877_50210523_+ | KLHDC1 | NA | -48.27681495 | 0.000361834 | 0.018290686 | 0 | 0.1885493 | 0 | 0.248273892 | 0 | 0.269077244 | 0 | 0.147428953 |
| chr18_20570900_20581692_+ | RBBP8 | hsa_circ_0047136 | -50.20997259 | 0.000442495 | 0.019609758 | 0 | 0.15083944 | 0 | 0.157992477 | 0 | 0.156961726 | 0 | 0.319429398 |
| chr5_64863340_64868113_+ | PPWD1 | hsa_circ_0072697 | -51.28312787 | 1.65E-05 | 0.00196193 | 0 | 3.243047963 | 0 | 0.225703538 | 0.30025172 | 1.233270701 | 0 | 1.105717148 |
| chr1_214564331_214571344_- | PTPN14 | hsa_circ_0008659 | -52.06386897 | 0.000357891 | 0.018290686 | 0 | 0.3770986 | 0 | 0.315984953 | 0 | 0.044846207 | 0 | 0.245714922 |
| chr3_183368084_183382827_+ | KLHL24 | NA | -53.17947014 | 0.000264551 | 0.016209734 | 0 | 0.30167888 | 0 | 0.090281415 | 0 | 0.179384829 | 0 | 0.294857906 |
| chr5_38523521_38530768_- | LIFR | hsa_circ_0072309 | -53.40553482 | 0.005126493 | 0.082926153 | 0 | 4.261214184 | 3.040091265 | 0.270844246 | 0 | 1.367809323 | 0 | 1.007431179 |
| chr10_123298106_123325218_- | FGFR2 | NA | -56.48359389 | 0.000152083 | 0.010250401 | 0 | 0.41480846 | 0 | 0.383696015 | 0 | 0.24665414 | 0 | 0.098285969 |
| chr17_67124760_67130883_- | ABCA6 | NA | -59.27552602 | 0.000394671 | 0.01841364 | 0 | 0.07541972 | 0 | 0.090281415 | 0 | 0.24665414 | 0 | 0.442286859 |
| chr4_166960491_166999182_+ | TLL1 | NA | -64.04599497 | 9.92E-05 | 0.00742904 | 0 | 0.15083944 | 0 | 0.47397743 | 0 | 0.201807933 | 0 | 0.294857906 |
| chr3_3197903_3215945_- | CRBN | hsa_circ_0003400 | -64.48846363 | 8.90E-05 | 0.006919776 | 0 | 0.45251832 | 0 | 0.18056283 | 0 | 0.336346555 | 0 | 0.196571937 |
| chr7_16298015_16317851_- | ISPD | hsa_circ_0079480 | -65.70981743 | 0.00011581 | 0.008363102 | 0 | 0.5656479 | 0 | 0.090281415 | 0 | 0.24665414 | 0 | 0.270286414 |
| chr4_25334805_25335610_+ | ZCCHC4 | hsa_circ_0001398 | -70.56029164 | 4.95E-05 | 0.004519474 | 0 | 0.52793804 | 0 | 0.406266368 | 0 | 0.112115518 | 0 | 0.294857906 |
| chr7_31912905_31920473_- | PDE1C | hsa_circ_0079753 | -75.76137512 | 6.13E-05 | 0.00516052 | 0 | 0.07541972 | 0 | 0.383696015 | 0 | 0.269077244 | 0 | 0.466858351 |
| chr18_20570900_20576425_+ | RBBP8 | hsa_circ_0047135 | -78.83630255 | 3.04E-05 | 0.002930685 | 0 | 0.41480846 | 0 | 0.135422123 | 0 | 0.448462073 | 0 | 0.319429398 |
| chr3_47651556_47719801_- | SMARCC1 | hsa_circ_0065244 | -81.28412251 | 2.70E-05 | 0.002867886 | 0 | 0.15083944 | 0 | 0.270844246 | 0 | 0.470885177 | 0 | 0.393143875 |
| chr10_117849252_117856275_- | GFRA1 | hsa_circ_0005239 | -84.95400132 | 9.15E-06 | 0.001423484 | 0 | 0.641067621 | 0 | 0.428836722 | 0 | 0.24665414 | 0 | 0.294857906 |
| chr3_183361268_183390272_+ | KLHL24 | hsa_circ_0001368 | -100.8614013 | 2.27E-06 | 0.000417496 | 0 | 0.30167888 | 0 | 0.541688491 | 0 | 0.336346555 | 0 | 0.516001336 |
| chr18_76856476_76914555_+ | ATP9B | hsa_circ_0004057 | -105.5360294 | 1.30E-06 | 0.000349906 | 0 | 0.49022818 | 0 | 0.428836722 | 0 | 0.358769659 | 0 | 0.516001336 |
| chr11_24927537_25004839_+ | LUZP2 | NA | -111.0277828 | 1.22E-06 | 0.000349906 | 0 | 0.1885493 | 0 | 1.105947336 | 0 | 0.538154488 | 0 | 0.294857906 |
| chr5_127474289_127488497_+ | SLC12A2 | hsa_circ_0006034 | -133.2272901 | 1.89E-06 | 0.000384981 | 0 | 0.07541972 | 0 | 0.993095567 | 0 | 0.762385525 | 0 | 0.491429843 |
| chr11_30516843_30602041_- | MPPED2 | hsa_circ_0021550 | -133.6237626 | 1.38E-06 | 0.000349906 | 0 | 0.15083944 | 0 | 0.925384506 | 0 | 0.829654836 | 0 | 0.442286859 |
| chr9_87317074_87325706_+ | NTRK2 | NA | -159.9472351 | 3.74E-07 | 0.000189709 | 0 | 0.905036641 | 0 | 0.925384506 | 0 | 0.605423799 | 0 | 0.540572828 |
| chr7_136935977_136939721_- | PTN | hsa_circ_0003949 | -160.8222046 | 6.82E-07 | 0.000275644 | 0 | 0.26396902 | 0 | 0.65454026 | 0 | 0.717539317 | 0 | 0.90914521 |
| chr1_215259711_215298093_+ | KCNK2 | hsa_circ_0016459 | -163.3085803 | 1.90E-06 | 0.000384981 | 0 | 0.11312958 | 0 | 1.399361935 | 0 | 0.986616561 | 0 | 0.491429843 |
| chr12_97886239_97954825_+ | RMST | NA | -286.583502 | 6.93E-08 | 7.01E-05 | 0 | 1.282135241 | 0 | 1.828198657 | 0 | 0.986616561 | 0 | 1.13028864 |
