# Supplementary figures and images for: The circRNA circSEPT9 mediated by E2F1 and EIF4A3 facilitates the carcinogenesis and development of triple-negative breast cancer
Source: Mol Cancer. 2020 Apr 7;19:73. doi: 10.1186/s12943-020-01183-9 (PMC7137343; doi:10.1186/s12943-020-01183-9)

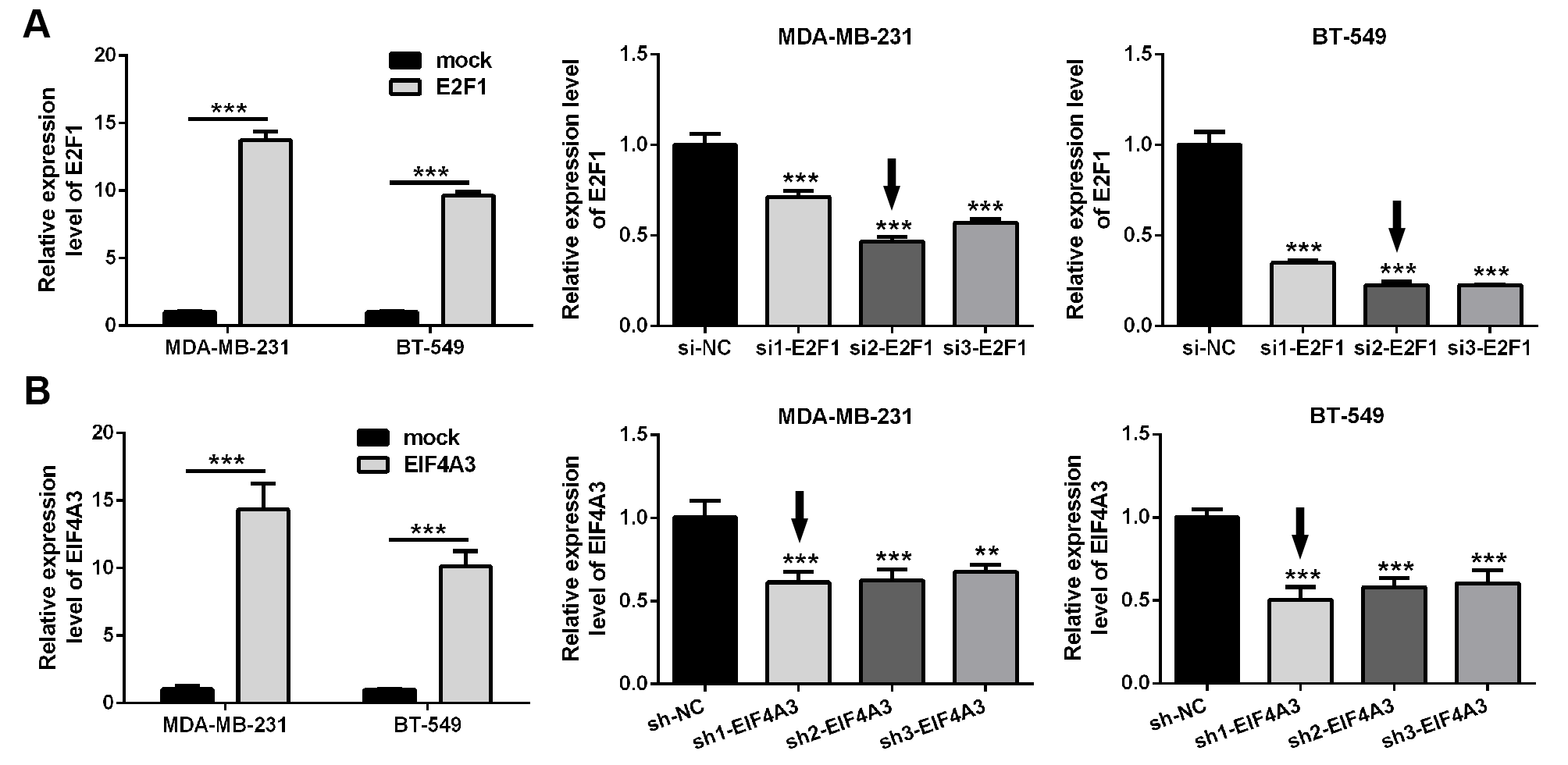

Supplement: Supplementary file 2 — Additional file 2: Figure S1. Relative expression levels of E2F1 and EIF4A3 were determined by qRT-PCR. (a) Relative expression of E2F1 expression were evaluated in TNBC cells transfected with E2F1 overexpression plasmids or siRNA. (b) The relative expression of EIF4A3 was detected in TNBC cells transfected with EIF4A3 overexpression or knockdown plasmids. The data are presented as the mean ± SD, **P < 0.01, ***P < 0.001. Figure S2. Overexpression of circSEPT9 increased proliferation, migration and invasion of TNBC cells by colony formation, EdU, CCK-8, wound healing and transwell assays. (a) Cell proliferation ability was evaluated by colony formation. (b) EdU assay of TNBC cells was performed to evaluate cell proliferation (magnification, × 100, Scale bar, 100 μm). (c) The growth curves of cells were measured by using CCK-8 assay. (d) Transwell invasion assay were used to assess the invasion abilities of TNBC cells (magnification, × 100, Scale bar, 100 μm). (e and f) The migration abilities of TNBC cells were measured by transwell migration (magnification, × 100, Scale bar, 100 μm) and wound healing (magnification, × 50, Scale bar, 100 μm) assays. The data are presented as the mean ± SD, *P < 0.05, **P < 0.01, ***P < 0.001. Figure S3. The expressions of LIF and LIF-STAT3 signal pathway related molecules were detected in tumor tissues of mice by IHC (magnification, × 200, Scale bar, 100 μm). [file 12943_2020_1183_MOESM2_ESM.zip › Additional file 2 Fig. S1.tif]

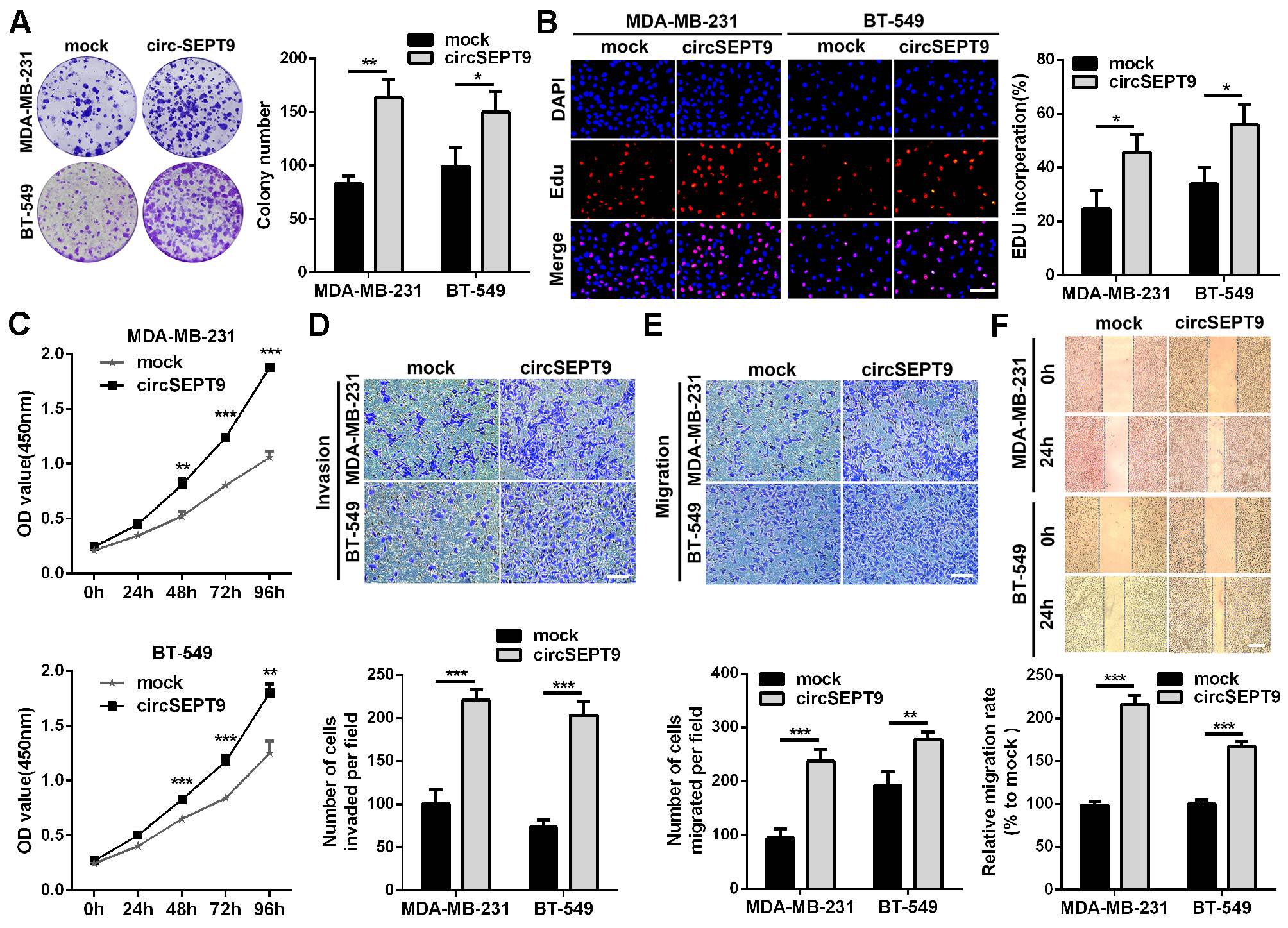

Supplement: Supplementary file 2 — Additional file 2: Figure S1. Relative expression levels of E2F1 and EIF4A3 were determined by qRT-PCR. (a) Relative expression of E2F1 expression were evaluated in TNBC cells transfected with E2F1 overexpression plasmids or siRNA. (b) The relative expression of EIF4A3 was detected in TNBC cells transfected with EIF4A3 overexpression or knockdown plasmids. The data are presented as the mean ± SD, **P < 0.01, ***P < 0.001. Figure S2. Overexpression of circSEPT9 increased proliferation, migration and invasion of TNBC cells by colony formation, EdU, CCK-8, wound healing and transwell assays. (a) Cell proliferation ability was evaluated by colony formation. (b) EdU assay of TNBC cells was performed to evaluate cell proliferation (magnification, × 100, Scale bar, 100 μm). (c) The growth curves of cells were measured by using CCK-8 assay. (d) Transwell invasion assay were used to assess the invasion abilities of TNBC cells (magnification, × 100, Scale bar, 100 μm). (e and f) The migration abilities of TNBC cells were measured by transwell migration (magnification, × 100, Scale bar, 100 μm) and wound healing (magnification, × 50, Scale bar, 100 μm) assays. The data are presented as the mean ± SD, *P < 0.05, **P < 0.01, ***P < 0.001. Figure S3. The expressions of LIF and LIF-STAT3 signal pathway related molecules were detected in tumor tissues of mice by IHC (magnification, × 200, Scale bar, 100 μm). [file 12943_2020_1183_MOESM2_ESM.zip › Additional file 2 Fig. S2.tif]

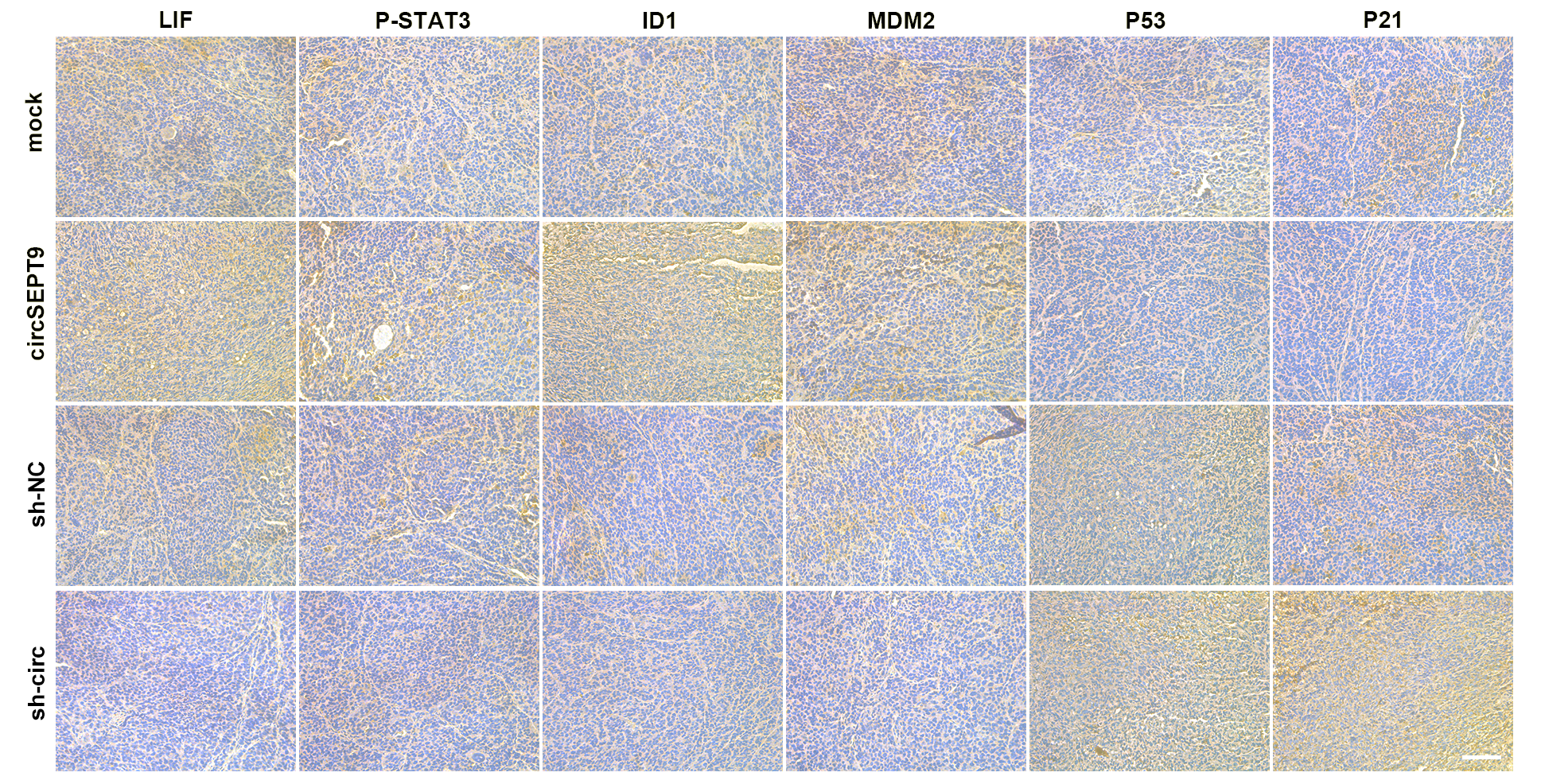

Supplement: Supplementary file 2 — Additional file 2: Figure S1. Relative expression levels of E2F1 and EIF4A3 were determined by qRT-PCR. (a) Relative expression of E2F1 expression were evaluated in TNBC cells transfected with E2F1 overexpression plasmids or siRNA. (b) The relative expression of EIF4A3 was detected in TNBC cells transfected with EIF4A3 overexpression or knockdown plasmids. The data are presented as the mean ± SD, **P < 0.01, ***P < 0.001. Figure S2. Overexpression of circSEPT9 increased proliferation, migration and invasion of TNBC cells by colony formation, EdU, CCK-8, wound healing and transwell assays. (a) Cell proliferation ability was evaluated by colony formation. (b) EdU assay of TNBC cells was performed to evaluate cell proliferation (magnification, × 100, Scale bar, 100 μm). (c) The growth curves of cells were measured by using CCK-8 assay. (d) Transwell invasion assay were used to assess the invasion abilities of TNBC cells (magnification, × 100, Scale bar, 100 μm). (e and f) The migration abilities of TNBC cells were measured by transwell migration (magnification, × 100, Scale bar, 100 μm) and wound healing (magnification, × 50, Scale bar, 100 μm) assays. The data are presented as the mean ± SD, *P < 0.05, **P < 0.01, ***P < 0.001. Figure S3. The expressions of LIF and LIF-STAT3 signal pathway related molecules were detected in tumor tissues of mice by IHC (magnification, × 200, Scale bar, 100 μm). [file 12943_2020_1183_MOESM2_ESM.zip › Additional file 2 Fig. S3.tif]
